# N1-Propargylguanosine Modified mRNA Cap Analogs: Synthesis, Reactivity, and Applications to the Study of Cap-Binding Proteins

**DOI:** 10.3390/molecules24101899

**Published:** 2019-05-17

**Authors:** Michal Kopcial, Blazej A. Wojtczak, Renata Kasprzyk, Joanna Kowalska, Jacek Jemielity

**Affiliations:** 1 College of Inter-Faculty Individual Studies in Mathematics and Natural Sciences, University of Warsaw, S. Banacha 2c, 02-097 Warsaw, Poland; m.kopcial@cent.uw.edu.pl (M.K.); rkasprzyk@biogeo.uw.edu.pl (R.K.); 2 Centre of New Technologies, University of Warsaw; S. Banacha 2c, 02-097 Warsaw, Poland; blazej.wojtczak@cent.uw.edu.pl; 3 Faculty of Physics, University of Warsaw; L. Pasteura 5, 02-093 Warsaw, Poland; jkowalska@fuw.edu.pl

**Keywords:** mRNA cap, 7-methylguanosine, DcpS, eIF4E, fluorescent probes, MST

## Abstract

The mRNA 5′ cap consists of N7-methylguanosine bound by a 5′,5′-triphosphate bridge to the first nucleotide of the transcript. The cap interacts with various specific proteins and participates in all key mRNA-related processes, which may be of therapeutic relevance. There is a growing demand for new biophysical and biochemical methods to study cap–protein interactions and identify the factors which inhibit them. The development of such methods can be aided by the use of properly designed fluorescent molecular probes. Herein, we synthesized a new class of m^7^Gp_3_G cap derivatives modified with an alkyne handle at the N1-position of guanosine and, using alkyne-azide cycloaddition, we functionalized them with fluorescent tags to obtain potential probes. The cap derivatives and probes were evaluated in the context of two cap-binding proteins, eukaryotic translation initiation factor (eIF4E) and decapping scavenger (DcpS). Biochemical and biophysical studies revealed that N1-propargyl moiety did not significantly disturb cap–protein interaction. The fluorescent properties of the probes turned out to be in line with microscale thermophoresis (MST)-based binding assays.

## 1. Introduction

A cap structure is present on 5′ end of all eukaryotic mRNAs. It plays significant roles in gene expression and mRNA metabolism. The cap consists of 7-methylguanosine bound by a 5′,5′-triphosphate bridge to the first nucleoside of the mRNA transcript [1]. The positively charged nucleobase and the negatively charged phosphate bridge are essential for retaining cap functionality in its interactions with cap-recognizing proteins [2,3,4]. In a cell, the cap is targeted by multiple proteins involved in mRNA-related processes such as eukaryotic translation initiation factor 4E (eIF4E), which is involved in translation initiation, and decapping scavenger (DcpS) or protein complex Dcp1/Dcp2, which participate in mRNA degradation. Certain cap-binding proteins have been identified as potential therapeutic targets. Elevated levels of eIF4E may induce tumorigenesis [5] and have been detected in numerous cancer cells [6,7]. Thus, eIF4E is a potential antitumor therapeutic target [8,9,10]. Furthermore, DcpS decapping enzyme, was found to be a molecular target for spinal muscular atrophy (SMA) [11]. Chemically modified cap analogs including fluorescent probes are useful tools for the study of cap-binding proteins. To this end, nucleotides labeled with antibodies [12], fluorescent tags [13,14,15,16], EPR tags [17], and radioisotopes [18] have been developed.

**Figure 1:**
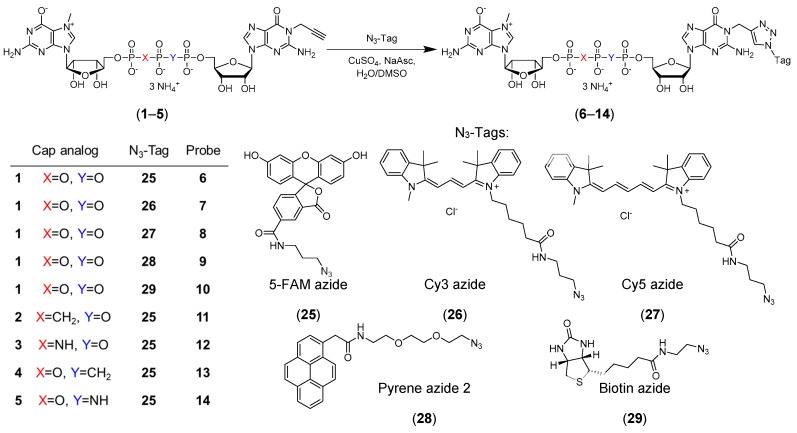
Synthesis of N1 functionalized dinucleotides (**6**–**14**) from N1-propargylated cap precursors (**1**–**5**) via a CuAAC reaction.

*Click chemistry* has been used to obtain these tools rapidly and efficiently [19,20,21,22,23,24,25,26]. However, for successful implementation, the modifications introduced into the cap must not disturb its interaction with the targeted protein(s). Thus, the development of new approaches to mRNA cap functionalization to structurally diversify the range of available probes is still desirable. Here, we designed and synthesized a set of novel m^7^Gp_3_G analogs bearing an alkyne moiety at the N1 position of guanosine (Figure 1, compounds **1**–**5**). To ensure their resistance to hydrolytic enzymes, certain analogs were additionally modified within a triphosphate chain (compounds **2**–**5**). Compounds **1**–**5** were then transformed into N1-labelled probes by a CuAAC reaction (Figure 1, compounds **6**–**14**). Recognition of the probes and their unmodified precursors by eIF4E and DcpS was validated to identify possible applications for these compounds.

## 2. Results

### 2.1. Cap Analogs Synthesis

**Scheme 1:**
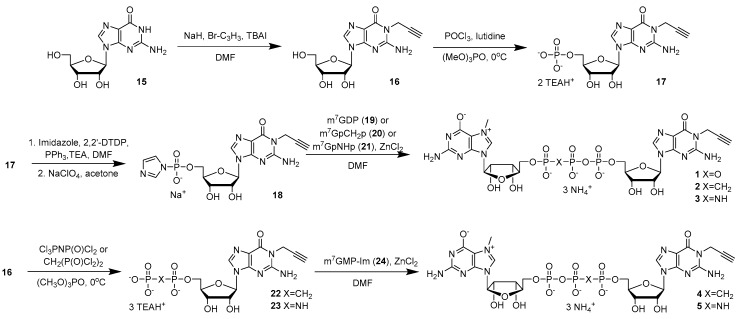
Synthesis of propargyl-modified cap analogs (**1**–**5**).

Cap analogs bearing propargyl moiety on the guanosine at N1 position (**1**–**5**) were derived from N1-propargylguanosine (**16**)**,** which was synthesized from unprotected nucleoside as previously described (Scheme 1) [27]. Guanosine (**15**), sodium hydride (NaH), and tetrabutylammonium iodide (TBAI) were suspended in anhydrous dimethylformamide (DMF) and propargyl bromide was added to generate N1-propargylguanosine (**16**) in 71% yield. Compound **16** was phosphorylated to N1-propargylguanosine 5′-*O*-monophosphate (**17**) with POCl_3_ in trimethyl phosphate [28]. After purification by ion exchange chromatography, compound **17** was converted to compound **18** by reacting it with imidazole in the presence of 2,2′-dithiodipyridine (DTDP), triethylamine (TEA), and triphenylphosphine (PPh_3_) followed by precipitation with sodium perchlorate (NaClO_4_) in acetone [29]. The respective yields were 54% and 95%. A cap analog bearing a propargyl moiety and an unmodified phosphate bridge (**1**) was obtained by coupling compound **18** with N7-methylguanosine 5′-*O*-diphosphate (m^7^GDP) (**19**) in DMF in the presence of ZnCl_2_. Cap analogs modified at the β,γ position (**2, 3**) were obtained in the similar manner, except that the m^7^GDP analog (m^7^GpCH_2_p (**20**) or m^7^GpNHp (**21**), respectively) was used in the reaction. To obtain cap analogs bearing a modification within phosphate bridge at the α,β-position (**4**–**5**), compound **16** was treated with either CH_2_(POCl_2_)_2_ or Cl_3_PNP(O)Cl_2_ in trimethyl phosphate to obtain N1-propargylguanosine 5′-*O*-(1,2-methylenephosphate) (**22**) or N1-propargylguanosine 5′-*O*-(1,2-imidodiphosphate) (**23**) in 75% and 63% yield, respectively [28]. Compounds **22** or **23** were coupled with N7-methylguanosine-5′-*O*-monophosphate-P-imidazolide (m^7^GMP-Im) (**24**) in DMF in the presence of ZnCl_2_ to obtain cap analogs **4** and **5,** respectively. The final products (**1–5**) were purified by ion exchange chromatography and the yields were 36–58%. The products were re-purified by semipreparative RP-HPLC, isolated as ammonium salts, and used for labeling in the CuAAC reaction and biophysical studies. The structures and purities of all new compounds were confirmed by HRMS, ^1^H NMR, and ^31^P NMR (Supplementary Information).

Reactivity of the cap analogs containing N1-propargylguanosine in CuAAC was evaluated. To this end, probes (**6**–**14**) were obtained by click chemistry in the reaction between commercially available fluorescent tags azide analogs (**25**–**28**) or biotin azide (**29**) [14] and cap analogs bearing the propargyl moiety **(1**–**5)** (Figure 1). An aqueous solution of cap analog (**1**–**5**) was mixed with a tag (**25**–**29**) dissolved in dimethyl sulfoxide (DMSO) at room temperature (RT) followed by the addition of aqueous solutions of CuSO_4_ and sodium ascorbate. The concentrations of the tags used for labeling ranged from 100–190 mM. HPLC analysis revealed that under these conditions, the conversion rates were in the range of 51–100%. The reactions were quenched by the addition of sodium ethylenediaminetetraacetate/sodium bicarbonate solution (Na_2_EDTA/NaHCO_3_), and the probes were purified by HPLC and isolated as ammonium salts. Using this approach, nine new probes were synthesized (Figure 1). To assess how substitution at the N1-position of guanosine influences mRNA cap recognition by specific proteins and enzymes, we performed a series of experiments with unlabeled cap analogs (**1**–**5**) and fluorescently labeled probes (**6**–**14**).

### 2.2. Binding Affinities to eIF4E

**Figure 2:**
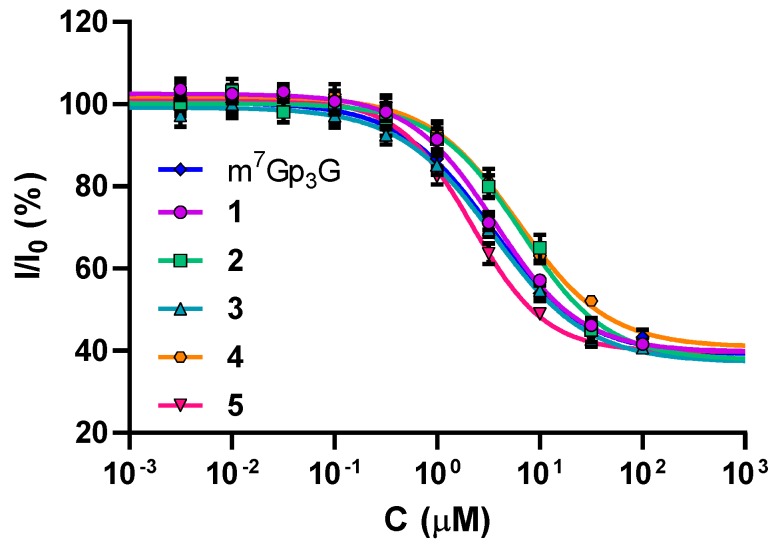
Dose–response curves for compounds **1**–**5** and m^7^Gp_3_G plotted from a competition binding assay for meIF4E [16]. eIF4E (75 nM) and a pyrene-labeled fluorescent probe (10 nM) were incubated at 30 °C with the indicated concentrations of the investigated compounds (**1**–**5** or m^7^Gp_3_G as a reference). IC_50_ and *K*_D_ values from triplicate experiments are shown in Table 1.

The prime role of the mRNA cap is to participate in initiation of translation through binding with eIF4E. Therefore, we investigated how the substitution at the N1 position influences the interaction between the cap analogs and eIF4E. To this end, we performed binding competition experiments with a *Py*-labeled m^7^GTP analog as previously described [16]. Thence, we determined IC_50_ values and calculated binding constants values (*K*_D_) for the cap analog-eIF4E complexes (Table 1, Figure 2). It was found that cap analogs **1**–**5** have affinities similar to that of the native cap structure (m^7^Gp_3_G) (Figure S1, Table S1). Additionally, the differences identified among studied compounds derived from triphosphate chain modifications were in agreement with previously published results [30,31,32].

The propargyl moiety introduced to the N1 position of guanosine had minimal effect on the interaction with eIF4E (Table S1). For this reason, these cap analogs are very promising as tools to study eIF4E.

### 2.3. Enzymatic Degradation Studies

**Figure 3:**
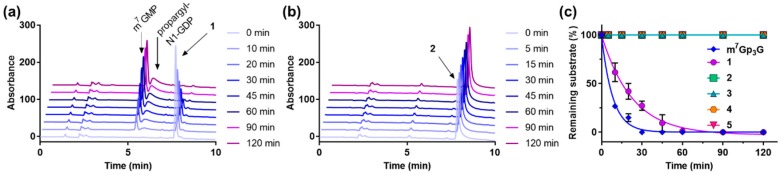
Enzymatic degradation of cap analogs **1**–**5** (30 µM) by human decapping scavenger enzyme (hDcpS) (30 nM) monitored by RP-HPLC. Representative time-dependent RP-HPLC profiles for compounds **1** (**a**) and **2** (**b**). Percentage of residual substrate as a function of time for all compounds (**c**). Data are average of triplicate experiments ± SD.

Susceptibility of the new cap analogs to hydrolysis by human decapping scavenger enzyme (hDcpS) was investigated to assess the influence of N1 substitution on cap analogs recognition by DcpS. Caps **1**–**5** or m^7^Gp_3_G (30 µM each) were incubated with hDcpS (30 nM) at 30 °C. Then, reaction samples were thermally deactivated and analyzed by RP-HPLC. Under these conditions, cap analog **1** was efficiently cleaved by DcpS but more slowly than m^7^Gp_3_G. All analogs modified within the phosphate bridge (**2**–**5**) were resistant to hDcpS under the applied conditions (Figure 3). The propargyl moiety at the N1 position of a second nucleobase slightly decreased the susceptibility of the new cap analogs to hDcpS cleavage but prevented neither the recognition nor the regioselectivity of the cleavage (Table S2).

**Figure 4:**
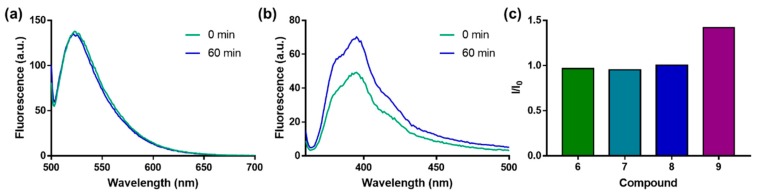
The influence of complete cleavage by hDcpS (30 nM) on the fluorescence properties of probes **6**–**9** (100 nM). Emission spectra before (0 min) and after (60 min) decapping scavenger (DcpS) cleavage for compounds **6** (**a**) and **9** (**b**); (**c**) The ratios of fluorescence intensity after and before cleavage (I/I_0_) for all studied probes.

Fluorescently labeled cap analogs that serve as DcpS substrates can be considered as activity probes. We performed an enzymatic cleavage test with 30 nM hDcpS and 30 µM probe to evaluate whether fluorescently labeled derivatives of cap analog **1** (probes **6**–**9**) can be used to monitor enzymatic activity via changes in fluorescence emission. For probes **6**–**8**, the fluorescence intensity at the emission maximum did not significantly change following complete cleavage by DcpS (Figure 4 and Figure S2). For the Py-labeled probe **9**, the fluorescence intensity at the emission maximum increased by 50% after complete DcpS cleavage (Figure 4c and Figure S3). Nevertheless, certain previously developed probes provided higher sensitivity than the one formulated here [15,16]. Modest changes in fluorescence emission upon the enzymatic cleavage of compounds **6**–**9** were independently confirmed with the nonspecific pyrophosphatase PDE-I (Figure S4).

The lack of fluorescence changes upon enzymatic cleavage suggests that the fluorescent labels attached to the N1 position of guanosine are relatively insensitive to alterations in the local environment. This characteristic is desirable in fluorescence polarization (FP) and microscale thermophoresis (MST) binding assays. Therefore, in the next step, we tested whether compounds **6**–**14** could serve as binding probes for either eIF4E or DcpS.

### 2.4. Evaluation of N1 Fluorescently Labeled Cap Analogs as Probes for Microscale Thermophoresis

First, we performed MST direct binding experiments with fluorescein-labeled probes **6**, **11**, or **12** and murine eIF4E (meIF4E).

**Figure 5:**
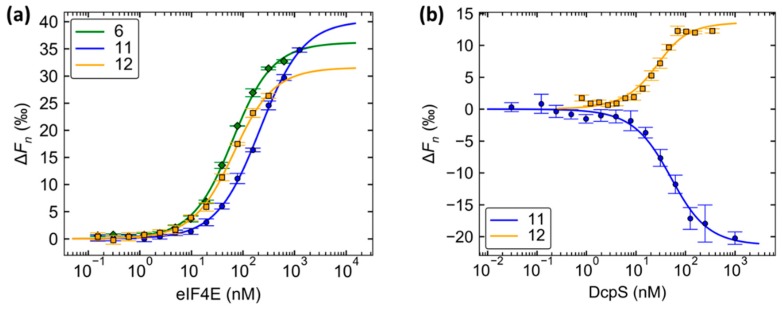
Direct binding microscale thermophoresis (MST) experiments with eIF4E and DcpS. (**a**) MST binding curves for probes **6, 11**, **12,** and meIF4E. Probe **6**, **11**, or **12** (50 nM) was mixed with a half-log dilution series of an meIF4E solution (10 µM–0.30 nM). (**b**) MST binding curves for probes **11** and **12** and DcpS. A solution of probe **11** or **12** (50 nM) was mixed with a dilution series of hDcpS solution (2.0 µM–0.061 nM for **11;** 700–1.6 nM for **12**). MST traces for each experiment are shown in Figures S5 and S6. Data were analyzed in Palmist v. 1.4.4 using 1:1 binding model. The *K*_D_ values were calculated from triplicate experiments and are shown in Table 2.

To this end, fluorescent probes (25 nM) were mixed with increasing concentrations of meIF4E and microscale thermophoresis was measured for each sample (Figure S5). Baseline corrected normalized fluorescences (*ΔF_n_*) determined from the MST traces were plotted as a function of protein concentration. *K*_D_ values could then be calculated from the resulting binding curves (Figure 5). Probes **6** and **12** showed similar affinities for meIF4E, which were higher than the affinity of probe **11** (Table 2). These findings align with those determined for the parent compounds **1**, **2**, and **3** (Table 1). Additionally, this suggests that labeling at the N1 position of guanosine does not interfere with already known effects of phosphate bridge modification.

With these promising results in mind, we decided to use the same approach for studying DcpS enzyme. For this purpose, cleavage-resistant probes **11** and **12** were used. Both probes were high-affinity ligands for DcpS. Notably, probe **12** had 2.5-fold greater affinity for DcpS than probe **11** (*K*_D_ = 14 and 38 nM, respectively). This is in agreement with previously reported data on the influence of triphosphate chain modifications on cap-DcpS interaction [30,31,32].

## 3. Discussion

In the present study, we aimed to develop new mRNA cap analogs amenable to click chemistry and convertible into potential binding or activity probes. We synthesized m^7^Gp_3_G dinucleotides bearing a “clickable” propargyl group handle at the N1 position of guanosine. Certain compounds were further modified within the triphosphate chain to modulate their susceptibility to enzymatic degradation. Utility of the probes in the investigation of cap-binding proteins and cap-specific enzymes was evaluated with eIF4E and DcpS, respectively. The N1-propargyl group did not significantly decrease the binding affinity for eIF4E significantly. Moreover, HPLC-monitored enzymatic degradation experiments showed that the N1 modification only slightly impaired recognition by DcpS. The N1-propargyl compounds were then converted into fluorescent probes with CuAAC. Characterization of the probes by emission spectroscopy revealed that the labels attached to the N1 position are comparatively insensitive to fluorescence intensity changes in response to protein binding or cleavage. Therefore, the labels are useful for experiments monitored by fluorescence intensity changes and for biophysical binding assays based on FP measurements or MST. To demonstrate this, we performed MST binding experiments using both eIF4E and DcpS in which we plotted reproducible binding curves and determined *K*_D_. For most compounds, the *K*_D_ were in the low nanomolar range (Table 2). To the best of our knowledge, the present study is the first example of the application of MST in the analysis of ligand binding to DcpS enzyme. Although compounds **1**–**5** do not possess extraordinary inhibitory properties in vitro experiments, the probe **12** tightly bound DcpS (*K*_D_ = 14 nM) and is, therefore, the preferred candidate for the development of an MST-based competitive binding assay to be used in the discovery and further in vitro evaluation of DcpS inhibitors. Studies on the specificity of N1-propargyl containing analogs for other cap binding proteins and decapping enzymes or possibility to incorporate them into RNA are underway.

## 4. Materials and Methods

### 4.1. Starting Materials and Chemical Reagents

Starting materials were purchased from Sigma-Aldrich Corp. (St. Louis, MO, USA) unless specified otherwise. Tetrabutylammonium iodide was purchased from Fluka Honeywell (Mexico City, Mexico) and imidazole was purchased from EMD Merck Millipore (Billerica, MA, USA). For syntheses under anhydrous conditions, solvents were dried over 4 Å molecular sieves for 24 h. Dichlorophosphorylphosphorimidoyl trichloride was prepared as described previously [34] and used in liquid form for subsequent reactions as problems with its crystallization occurred.

### 4.2. Chromatography and Optical Density Measurements

The nucleotides (**1**–**5**, **17**, **22**, and **23**) were purified by ion-exchange chromatography on a DEAE Sephadex A-25 (HCO_3_^−^ form) column. The column was loaded with reaction mixture and washed with water until the eluate no longer precipitated in the presence of AgNO_3_ solution to elute solvents and salts that do not bind to the column. Nucleotides were eluted with triethylammonium bicarbonate (TEAB) gradients in deionized water: 0–0.7 M for nucleoside monophosphates, 0–1.0 M for nucleoside diphosphates, and 0–1.2 M for nucleoside triphosphates. The collected fractions were analyzed spectrophotometrically at 260 nm and fractions containing the desired nucleotides were analyzed by RP-HPLC and pooled. Yields were calculated on the basis of milliunit optical density measurements (mODU, absorbance of solution multiplied by its volume in ml) of the isolated products and their corresponding starting materials (nucleotides or nucleotide P-imidazolide derivatives). Optical measurements were performed in 0.1 M phosphate buffer pH 7.0. Afterwards, evaporation under reduced pressure with repeated additions of 96% and then 99.8% ethanol was carried out to decompose TEAB and remove residual water. The nucleotides were isolated as triethylammonium (TEAH^+^) salts. The final products were re-purified on semipreparative RP-HPLC. The products, after repeated freeze-drying, were isolated as ammonium salts.

### 4.3. Analytical and Preparative Reversed-Phase (RP) HPLC

Analytical HPLC was performed on an Agilent Tech Series 1200 or Agilent Tech. Series 1260 Infinity using a Supelcosil LC-18-T HPLC column (4.6 mm × 250 mm; flow rate 1.3 mL/min) or a Supelcosil LC-8 HPLC column (4.6 mm × 250 mm flow rate 0.75 mL/min) (Agilent Technologies, Santa Clara, CA, USA).

UV-detection was performed at 254 nm for nucleotides or at the absorption maximum wavelengths of specific dyes.

The HPLC methods used in the present study were as follows:

- Method A–eluant A was 0.05 M ammonium acetate buffer (pH 5.9); eluant B was a 1:1 (*v/v*) eluant A:MeOH mixture; eluant B gradient was 0–100% over 15 min; column was Supelcosil LC-18-T;
- Method B–eluant A was 0.05 M ammonium acetate buffer (pH 5.9); eluant B was 1:1 (*v/v*) eluant A:MeOH; eluant B gradient was 0–50% over 15 min; column was Supelcosil LC-18-T;
- Method C–eluant A was 0.05 M ammonium acetate buffer (pH 5.9); eluant B was acetonitrile; eluant B gradient was 0–100% over 15 min; column was Supelcosil LC-8;
- Method D–eluant A was 0.05 M ammonium acetate buffer (pH 5.9); eluant B is acetonitrile; eluant B gradient was 0–50% over 15 min; column was Supelcosil LC-8;
- Method E–eluant A was 0.1 M phosphate buffer (pH 6.0); eluant B was 1:1 (*v/v*) eluant A:MeOH; eluant B gradient was 0–50% over 15 min; column was Supelcosil LC-18-T.

### 4.4. NMR and MS Analyses

The structure and purity of probes were confirmed by high-resolution mass spectrometry with negative or positive electrospray ionization (HRMS (−) ESI or HRMS (+) ESI). Nucleotide derivative structures were confirmed by high-resolution mass spectrometry with negative or positive electrospray ionization (HRMS (−) ESI or HRMS (+) ESI) and ^1^H NMR, ^13^C NMR, or ^31^P NMR. Mass spectra were recorded on a Thermo Scientific LTQ OrbitrapVelos spectrometer (Thermo Fisher Scientific, Waltham, MA, USA). NMR spectra were recorded on a Varian INOVA 400 MHz or 500 MHz spectrometer fitted with a high-stability temperature unit and a 5 mm 4NUC probe at 25 °C unless stated otherwise and at 399.94/500.61 MHz (^1^H NMR), 100.57/125.80 MHz (^13^C NMR), or 161.90/202.49 MHz (^31^P NMR). The ^1^H NMR, ^13^C NMR, and ^31^P NMR chemical shifts were reported in ppm and referenced to respective internal standards: Sodium 3-(trimethylsilyl)-2,2′,3,3′ tetradeuteropropionate (TSP) and 20% phosphorus acid in D_2_O. Signals in ^1^H NMR dinucleotide spectra were assigned on the basis of their 2D NMR spectra (gDQCOSY, gHSQCAD).

### 4.5. Chemical Syntheses

#### 4.5.1. P1-(N1-Propargylguanosin-5′-yl) P3-(N7-methylguanosin-5′-yl) triphosphate (m^7^Gp_3_G-N1-propargyl) (**1**)

The m^7^GDP (TEAH^+^ salt, 50.0 mg, 0.066 mmol, 1.0 equivalent) (**19**) and compound **18** (Na^+^ salt, 46.7 mg, 0.098 mmol, 1.5 equivalent) were suspended in anhydrous DMF (1.0 mL) followed by addition of anhydrous ZnCl_2_ (89.7 mg, 0.66 mmol, 10 equivalent). The mixture was vigorously shaken until the reagents dissolved. The reaction progress was monitored by RP-HPLC. After completion (24 h), an appropriate amount of EDTA solution (Na_2_EDTA, 246.8 mg, 0.66 mmol, 10 equivalent) was added to the mixture to disassociate the nucleotide-metal complex, then the pH of the mixture was adjusted to 6 with solid NaHCO_3_, followed by purification with DEAE-Sephadex and isolated as TEAH^+^ salts (43.6 mg, 862 mODU, 0.038 mmol, 58%).Triethylammonium salts were then re-purified by semipreparative RP-HPLC and after repeated freeze-drying, were isolated as ammonium salts. HPLC method A t_R_ = 5.539 min. ^1^H NMR (deuterium oxide, 400 MHz) δ 7.98 (1H, s), 5.89 (1H, d, *J =* 3.7 Hz), 5.80 (1H, d, *J =* 6.2 Hz), 4.86 (1H, dd, *J =* 7.0, 2.4 Hz), 4.72 (1H, t, *J =* 6.2, 5.2 Hz), 4.55 (1H, t, *J =* 5.0, 3.7 Hz), 4.49 (2H, dd, *J =* 5.2, 3.3 Hz), 4.43 (1H, t, *J =* 5.0 Hz), 4.38–4.32 (3H, m), 4.29–4.22 (3H, m), 4.05 (3H, s), 2.76 (1H, t, *J =* 2.4 Hz). ^31^P NMR (deuterium oxide, 162 MHz) δ −10.62 (2P, dq, *J =* 19.6, 6.5 Hz), −22.24 (1P, t, *J =* 19.6 Hz). HRMS ESI (−) *m*/*z* Calc. for C_24_H_30_N_10_O_18_P_3_^−^ [M − H]^−^: 839.0958 found: 839.0965.

#### 4.5.2. P1-(N7-Methylguanosin-5′-yl) P3-(N1-propargylguanosin-5′-yl) 1,2-methylenetriphosphate (m^7^GpCH_2_ppG-N1-propargyl) (**2**)

The m^7^GpCH_2_p (28.4 mg, 60.0 µmol) (**20**), compound **18** (39.5 mg, 60.0 µmol, 1 equivalent), and ZnCl_2_ (65.3 mg, 480.1 µmol, 8 equivalent) were suspended in anhydrous DMF (601 µL). The mixture was stirred overnight at RT and quenched by the addition of 8.9 mL of Na_2_EDTA/NaHCO_3_ solution (20 g/L and 10 g/L, respectively). Side product propargyl-N1-GppG-N1-propargyl was observed. The product was purified with ion exchange chromatography on DEAE-Sephadex resin using TEAB buffer gradient (0.0–1.2 M) and isolated as TEAH^+^ salt (703 mODU, 31.1 µmol, 52%). The product was re-purified using semi-preparative RP-HPLC. HPLC method A t_R_ = 5.641 min. NMR: ^1^H NMR (400 MHz, deuterium oxide) δ 9.28 (1H, s), 8.10 (1H, s), 5.95 (1H, d, *J* = 4.2 Hz), 5.84 (1H, d, *J* = 5.5 Hz), 4.86 (2H, dd, *J* = 6.1, 2.5 Hz), 4.70 (1H, t, *J* = 5.5 Hz), 4.63 (1H, t, *J* = 4.2 Hz), 4.51–4.48 (2H, m), 4.38–4.31 (2H, m), 4.31–4.16 (4H, m), 4.07 (3H, s), 2.76 (1H, s), 2.40 (2H, t, *J* = 20.3 Hz); ^31^P NMR (162 MHz, deuterium oxide) δ 17.49–16.82 (1P, m), 7.63–6.82 (1P, m), −11.14 (1P, ddt, *J* = 1026.2, 25.6, 5.5 Hz). HRMS ESI (−) *m*/*z* Calc. for C_25_H_32_N_10_O_17_P_3_^−^ [M − H]^−^: 837.1165 found: 837.1177.

#### 4.5.3. P1-(N7-Methylguanosin-5′-yl) P3-(N1-propargylguanosin-5′-yl) 1,2-imidotriphosphate (m^7^GpNHppG-N1-propargyl) (**3**)

The m^7^GpNHp (TEAH^+^ salt, 50 mg, 0.066 mmol, 1.0 equivalent) (**21**) and compound **18** (Na^+^ salt, 46.7 mg, 0.098 mmol, 1.5 equivalent) were suspended in anhydrous DMF (1.0 mL) followed by addition of anhydrous ZnCl_2_ (89.7 mg, 0.66 mmol, 10 equivalent). The mixture was vigorously shaken until the reagents dissolved. Reaction progress was monitored by RP-HPLC. After completion (24 h), an appropriate amount of EDTA solution (Na_2_EDTA, 246.8 mg, 0.66 mmol, 10 equivalent) was added to disassociate the nucleotide–metal complex, adjusted to pH 6 with solid NaHCO_3_. The product was purified with ion exchange chromatography on DEAE-Sephadex in a TEAB buffer gradient (0.0–1.2 M) and isolated as TEAH^+^ salt (33.2 mg, 657 mODU, 0.029 mmol, 44%). The product was re-purified with semi-preparative RP-HPLC. ^1^H NMR (deuterium oxide, 400 MHz) δ 9.23 (1H, s), 8.11 (1H, d, *J =* 0.8 Hz), 5.94 (1H, d, *J =* 3.7 Hz), 5.84 (1H, d, *J =* 5.7 Hz), 4.87 (2H, dd, *J =* 5.9, 2.5 Hz), 4.72 (1H, t, *J =* 5.7 Hz), 4.60 (1H, t, *J =* 4.7, 3.8 Hz), 4.51–4.46 (2H, m), 4.37 (1H, dd, *J =* 5.2, 2.5 Hz), 4.35–4.32 (1H, m), 4.31–4.13 (4H, m), 4.10–4.07 (3H, m), 3.21 (1H, q, *J =* 7.3 Hz), 2.76 (1H, t, *J =* 2.5 Hz), 1.29 (1H, t, *J =* 7.3 Hz). ^31^P NMR (deuterium oxide, 162 MHz) δ 1.47–1.24 (1P, m), −8.90 (1P, dt, *J =* 20.0, 5.5 Hz), −9.37 (1P, dd, *J =* 20.0, 5.9 Hz). HRMS ESI (−) *m*/*z* Calc. for C_24_H_31_N_11_O_17_P_3_^−^ [M − H]^−^: 838.1112 found: 838.1126.

#### 4.5.4. P1-(N7-Methylguanosin-5′-yl) P3-(N1-propargylguanosin-5′-yl) 2,3-methylenetriphosphate (m^7^GppCH_2_pG-N1-propargyl) (**4**)

Compound **22** (37.3 mg, 54.8 µmol), m^7^GMP-Im (Na^+^ salt, 25.0 mg, 55.7 µmol, 1.0 equivalent) (**24**), and ZnCl_2_ (62.1 mg, 456.6 µmol, 8.3 equivalent) were suspended in anhydrous DMF (547 µL). The reaction mixture was stirred overnight at RT and quenched by the addition of 8.5 mL of Na_2_EDTA/NaHCO_3_ aqueous solution (20 g/L and 10 g/L, respectively). Traces of the side product m^7^G-pp-m^7^G were observed. The product was purified using ion exchange chromatography on DEAE-Sephadex resin in TEAB buffer gradient (0.0–1.2 M) and isolated as TEAH^+^ salt (515 mODU, 22.8 µmol, 42%). The product was repurified with semi-preparative RP-HPLC. HPLC method A t_R_ = 5.741 min. ^1^H NMR (400 MHz, deuterium oxide) δ 9.15 (1H, s), 8.30 (1H, s), 5.95 (1H, d, *J* = 3.5 Hz), 5.86 (1H, d, *J* = 5.4 Hz), 4.86 (2H, dd, *J* = 4.8, 2.5 Hz), 4.72 (1H, t, *J* = 5.4 Hz), 4.60 (1H, dd, *J* = 4.8, 3.5 Hz), 4.50 (1H, dd, *J* = 4.8, 4.3 Hz), 4.47 (1H, t, *J* = 5.4 Hz), 4.37 (1H, t, *J* = 5.4, 2.5 Hz), 4.35–4.33 (1H, m), 4.21 (4H, dddd, *J* = 22.3, 11.2, 6.2, 3.8 Hz), 4.08 (3H, s), 2.76 (1H, t, *J* = 2.5 Hz), 2.40 (2H, t, *J* = 20.4 Hz); ^31^P NMR (162 MHz, deuterium oxide) δ 17.26–16.77 (1P, m), 7.76–7.10 (1P, m), −11.17–−11.50 (1P, m). HRMS ESI (−) *m*/*z* Calc. for C_25_H_32_N_10_O_17_P_3_^−^ [M − H]^−^: 837.1165 found: 837.1174.

#### 4.5.5. P1-(N7-Methylguanosin-5′-yl) P3-(N1-propargylguanosin-5′-yl) 2,3-imidotriphosphate (m^7^GppNHpG-N1-propargyl) (**5**)

Compound **23** (TEAH^+^ salt, 50 mg, 0.064 mmol, 1.0 equivalent) and m^7^GMP-Im (Na^+^ salt, 43.2 mg, 0.096 mmol, 1.5 equivalent) (**24**) were suspended in anhydrous DMF (1.0 mL) followed by the addition of anhydrous ZnCl_2_ (87.0 mg, 10 equivalent, 0.64 mmol). The mixture was vigorously shaken until the reagents dissolved. The reaction progress was monitored by RP-HPLC. After completion (24 h), an appropriate amount of EDTA solution (Na_2_EDTA, 239.4 mg, 0.64 mmol) was added to disassociate the nucleotide–metal complex, adjusted to pH 6 with solid NaHCO_3_. The product was purified with ion exchange chromatography on DEAE-Sephadex in TEAB buffer gradient (0.0–1.2 M) and isolated as TEAH^+^ salt. Yield: (26.3 mg, 520 mOD, 0.023 mmol, 36%). Triethylammonium salts were then repurified by semipreparative RP-HPLC and after repeated freeze-drying, were isolated as ammonium salts. HPLC method A t_R_ = 5.608 min. ^1^H NMR (deuterium oxide, 400 MHz) δ 8.06 (1H, s), 5.93 (1H, d, *J =* 3.5 Hz), 5.82 (1H, d, *J =* 6.1 Hz), 4.87 (1H, d, *J =* 2.6 Hz), 4.85 (1H, d, *J =* 2.6 Hz), 4.74 (1H, t, *J =* 6.1, 5.1 Hz), 4.58 (1H, t, *J =* 4.9, 3.5 Hz), 4.49 (1H, t, *J =* 5.1, 3.6 Hz), 4.45 (1H, t, *J =* 4.9 Hz), 4.38–4.32 (3H, m), 4.29–4.22 (1H, m), 4.21–4.15 (2H, m), 4.07 (3H, s), 2.75 (1H, t, *J =* 2.6 Hz). ^31^P NMR (deuterium oxide, 162 MHz) δ 1.40–0.96 (1P, m), −8.50 to −9.12 (2P, m). HRMS ESI (−) *m*/*z* Calc. for C_24_H_31_N_11_O_17_P_3_^−^ [M − H]^−^: 838.1112 found: 838.1129.

#### 4.5.6. Probe 6 (m^7^Gp_3_G-N1-5FAM)

An aqueous solution of **1** (0.93 mg, 1.04 µmol, 175 mM, 5.96 µL) and DMSO solution of **25** (0.65 mg, 1.6 µmol, 187 mM, 8.33 µL) were mixed together. Then, an aqueous solution of CuSO_4_·5 H_2_O (0.13 mg, 0.50 µmol, 440 mM, 1.19 µL) and an aqueous solution of sodium ascorbate (2.07 mg, 10.40 µmol, 5.0 M, 2.09 µL) were added. The resulting mixture was shaken for 1 h at RT. The reaction mixture was quenched by the addition of 8.8 µL of Na_2_EDTA/NaHCO_3_ solution (20 g/L and 10 g/L, respectively) and diluted with H_2_O:DMSO (1:1, *v/v*) mixture up to 500 µL. The product was purified with RP HPLC and isolated as NH_4_^+^ salt. HPLC method D t_R_ = 10.623 min. HRMS (−) ESI *m/z* found 641.0989, calc. for C_47_H_45_N_14_O_24_P_3_^2−^: 641.0972.

#### 4.5.7. Probe 7 (m^7^Gp_3_G-N1-Cy3)

An aqueous solution of **1** (0.99 mg, 1.12 µmol, 175 mM, 6.44 µL) and DMSO solution of **26** (2.56 mg, 1.47 µmol, 134 mM, 11.0 µL) were mixed together. Then, an aqueous solution of sodium ascorbate (2.22 mg, 11.22 µmol, 5.0 M, 2.24 µL) and an aqueous solution of CuSO_4_·5 H_2_O (0.14 mg, 0.56 µmol, 440 mM, 1.28 µL) were added. The resulting mixture was shaken for 1 h at RT. Reaction was quenched by the addition of 12.0 µL of Na_2_EDTA/NaHCO_3_ solution (20 g/L and 10 g/L, respectively) and diluted with 150 µL of H_2_O:DMSO mixture (1:2, *v/v*). The product was purified with RP HPLC and isolated as NH_4_^+^ salt. HPLC method C t_R_ = 12.469 min. HRMS (−) ESI *m/z* found 1377.4400, calc. for C_57_H_72_N_16_O_19_P_3_^−^: 1377.4372.

#### 4.5.8. Probe 8 (m^7^Gp_3_G-N1-Cy5)

An aqueous solution of **1** (0.57 mg, 0.64 µmol, 1 mM, 3.66 µL) and DMSO solution of **27** (0.64 mg, 1.06 µmol, 100 mM, 10.60 µL) were mixed together. Then, an aqueous solution of sodium ascorbate (2.22 mg, 11.22 µmol, 5.0 M, 2.24 µL) and an aqueous solution of CuSO_4_·5 H_2_O (0.14 mg, 0.56 µmol, 440 mM, 1.28 µL) were added. Reaction was vortexed for 50 min at RT. Reaction was quenched by the addition of 10.5 µL of Na_2_EDTA/NaHCO_3_ solution (20 g/L and 10 g/L, respectively) and diluted with 200 µL of H_2_O:DMSO mixture (1:1, *v/v*). The product was purified with RP HPLC and isolated as NH_4_^+^ salt. HPLC method C t_R_ = 12.966 min. HRMS (−) ESI *m*/*z* found 1403.4553, calc. for C_59_H_74_N_16_O_19_P_3_^−^: 1403.4529.

#### 4.5.9. Probe 9 (m^7^Gp_3_G-N1-Py)

An aqueous solution of **1** (0.63 mg, 0.707 µmol, 150 mM, 4.71 µL) and DMSO solution of **28** (0.38 mg, 0.913 µmol, 101 mM, 7.07 µL) were mixed together. Then, an aqueous solution of sodium ascorbate (1.40 mg, 7.07 µmol, 5.0 M, 1.40 µL) and an aqueous solution of CuSO_4_·5 H_2_O (0.09 mg, 0.35 µmol, 440 mM, 0.80 µL) were added. Reaction was vortexed for 48 h at RT. Reaction was quenched by the addition 6.5 µL of Na_2_EDTA/NaHCO_3_ solution (20 g/L and 10 g/L, respectively) and diluted with 200 µL of H_2_O:DMSO mixture (1:1, *v/v*). The product was purified with RP-HPLC isolated as a NH_4_^+^ salt. HPLC method C t_R_ = 10.924 min. HRMS ESI (−) *m*/*z* Calc. for C_48_H_54_N_14_O_21_P_3_^−^ [M − H]^−^: 1255.2801 found: 1255.2818.

#### 4.5.10. Probe 10 (m^7^Gp_3_G-N1-biotin)

An aqueous solution of **1** (0.80 mg, 0.90 µmol, 175 mM, 5.13 µL) and DMSO solution of **29** (0.41 mg, 1.31 µmol, 146 mM, 9.0 µL) were mixed together. Then, an aqueous solution of sodium ascorbate (1.78 mg, 8.98 µmol, 5.0 M, 1.80 µL) and an aqueous solution of CuSO_4_·5 H_2_O (0.11 mg, 0.45 µmol, 440 mM, 1.02 µL) were added. Reaction was vortexed for 1 h at RT. Reaction was quenched by the addition of 8.5 µL of Na_2_EDTA/NaHCO_3_ solution (20 g/L and 10 g/L, respectively) and diluted with 200 µL of H_2_O:DMSO mixture (1:1, *v/v*). The product was purified with RP-HPLC isolated as a NH_4_^+^ salt. HPLC method C t_R_ = 8.039 min. HRMS (−) ESI *m*/*z* found 1151.2345, calc. for C_36_H_50_N_16_O_20_P_3_S^−^: 1151.2321.

#### 4.5.11. Probe 11 (m^7^GpCH_2_ppG-N1-5FAM)

An aqueous solution of **2** (0.69 mg, 0.78 µmol, 175 mM, 4.47 µL) and DMSO solution of **25** (0.49 mg, 1.17 µmol, 188 mM, 6.24 µL) were mixed together. Then, an aqueous solution of sodium ascorbate (1.54 mg, 7.8 µmol, 5.0 M, 1.56 µL) and an aqueous solution of CuSO_4_·5 H_2_O (0.10 mg, 0.39 µmol, 440 mM, 0.89 µL) were added. Reaction was vortexed overnight at RT. Reaction was quenched by the addition 7.3 µL of Na_2_EDTA/NaHCO_3_ solution (20 g/L and 10 g/L, respectively) and diluted with 200 µL of H_2_O:DMSO mixture (1:1, *v/v*). The product was purified with RP-HPLC isolated as a NH_4_^+^ salt. HPLC method D t_R_ = 10.412 min. HRMS (−) ESI *m*/*z* found 640.1091, calc. for C_48_H_47_N_14_O_23_P_3_^2−^: 640.1076.

#### 4.5.12. Probe 12 (m^7^GpNHppG-N1-5FAM)

An aqueous solution of **3** (0.84 mg, 0.94 µmol, 175 mM, 5.36 µL) and DMSO solution of **25** (0.59 mg, 1.41 µmol, 188 mM, 7.48 µL) were mixed together. Then, an aqueous solution of CuSO4·5 H^2^O (0.12 mg, 0.47 µmol, 440 mM, 1.07 µL) and an aqueous solution of sodium ascorbate (1.86 mg, 9.38 µmol, 5.0 M, 1.88 µL) were added. Reaction was vortexed overnight at RT. Reaction was quenched by the addition of 8.2 µL of Na_2_EDTA/NaHCO_3_ solution (20 g/L and 10 g/L, respectively) and diluted with water/DMSO H_2_O:DMSO mixture (1:1, *v/v*). The product was purified with RP-HPLC isolated as a NH_4_^+^ salt. HPLC method D t_R_ = 10.155 min. HRMS (−) ESI *m*/*z* found 1282.2221, calc. for C_47_H_47_N_15_O_23_P_3_^−^: 1282.2182, HRMS (−) ESI *m*/*z* found 640.6076, calc. for C_47_H_46_N_15_O_23_P_3_^2−^: 640.6052.

#### 4.5.13. Probe 13 (m^7^GppCH_2_pG-N1-5FAM)

An aqueous solution of **4** (0.46 mg, 0.52 µmol, 150 mM, 3.46 µL) and DMSO solution of **25** (0.32 mg, 0.777 µmol, 150 mM, 5.18 µL) were mixed together. Then, an aqueous solution of sodium ascorbate (1.03 mg, 5.20 µmol, 5.0 M, 1.04 µL) and an aqueous solution of CuSO_4_·5 H_2_O (0.76 mg, 0.30 µmol, 440 mM, 0.69 µL) were added. Reaction was vortexed for 30 min at RT. Reaction was quenched by the addition 5.5 µL of Na_2_EDTA/NaHCO_3_ solution (20 g/L and 10 g/L, respectively) and diluted with 200 µL of H_2_O:DMSO mixture (1:1, *v/v*). The product was purified with RP-HPLC isolated as a NH_4_^+^ salt. HPLC method D t_R_ = 10.649 min. HRMS (−) ESI *m*/*z* found 640.1089, calc. for C_48_H_47_N_14_O_23_P_3_^2−^: 640.1076.

#### 4.5.14. Probe 14 (m^7^GppNHpG-N1-5FAM)

An aqueous solution of **10** (0.66 mg, 0.74 µmol, 150 mM, 4.94 µL) and DMSO solution of **25** (0.46 mg, 1.113 µmol, 150 mM, 7.42 µL) were mixed together. Then, an aqueous solution of sodium ascorbate (1.47 mg, 7.42 µmol, 5.0 M, 1.48 µL) and an aqueous solution of CuSO_4_·5 H_2_O (0.09 mg, 0.37 µmol, 440 mM, 0.84 µL) were added. The resulting mixture was vortexed for 2.5 h at RT. Reaction was quenched by the addition 6.9 µL of Na_2_EDTA/NaHCO_3_ solution (20 g/L and 10 g/L, respectively) and diluted with 200 µL of H_2_O:DMSO mixture (1:1, *v/v*). The product was purified with RP-HPLC. HPLC method D t_R_ = 10.712 min. HRMS (−) ESI *m*/*z* found 640.6068, calc. for C_47_H_46_N_15_O_23_P_3_^2−^: 640.6052.

#### 4.5.15. N1-propargylguanosine (**16**)

Compound **16** was prepared according to Hienzsch et al. [27] with minor modifications. Guanosine (1.99 g, 7.03 mmol) (**15**) was placed in a round-bottom flask and suspended in anhydrous DMF (70 mL) under an inert argon atmosphere, then sodium hydride (60% dispersion in mineral oil, 379 mg, 9.475 mmol, 1.34 equivalent) and tetrabutylammonium iodide (TBAI, 560 mg, 1.74 mmol, 0.25 equivalent) were added, and suspension continued to stir for approx. 30 min at RT followed by the addition of propargyl bromide (1.0 mL, 9.4 mmol, 1.33 equivalent). Suspension turned from whitish to yellowish upon addition. The mixture was left to stir overnight at RT. After 20 h, the mixture was clear, orange-red. When the TLC plate showed completion of the reaction, the solvent was evaporated to obtain oily residue. To the obtained oily residue, CH_2_Cl_2_ was added and the precipitate was dissolved in H_2_O:MeOH. Aqueous fractions were purified with Revelerys ^®^ Prep (Buchi) in CH_2_Cl_2_:MeOH gradient (up to 30% (*v/v*) of MeOH). Yield (1.60 g, 4.98 mmol, 71%). R_f_ = 0.7 (CH_2_Cl_2_:MeOH, 1:1, *v/v*). ^1^H NMR (DMSO-*d*_6_, 400 MHz) δ 7.98 (1H, s), 7.21 (2H, s), 5.71 (1H, d, *J* = 6.1 Hz), 5.41 (1H, d, *J* = 6.0 Hz), 5.13 (1H, d, *J* = 4.6 Hz), 5.01 (1H, t, *J* = 5.5 Hz), 4.80 (2H, t, *J* = 4.1, 2.5 Hz), 4.42 (1H, q, *J* = 5.9, 4.9 Hz), 4.10 (1H, q, *J* = 4.9, 3.5 Hz), 3.87 (1H, q, *J* = 4.3, 3.5 Hz), 3.62 (1H, dt, *J* = 11.8, 5.5, 4.3 Hz), 3.53 (1H, dt, *J* = 11.8, 5.5, 4.3 Hz), 3.22 (1H, t, *J* = 2.5 Hz). ^13^C NMR (DMSO-*d*_6_, 101 MHz) δ 157.06, 154.71, 151.26, 137.58, 116.95, 87.48, 86.70, 80.22, 75.37, 75.15, 71.89, 62.87, 31.73, 27.00. HRMS ESI (+) *m*/*z* Calc. for C_13_H_16_N_5_O_5_^+^ [M + H]^+^: 322.1146 found: 322.1146.

#### 4.5.16. N1-Propargylguanosine-5′-*O*-monophosphate (propargyl-N1-GMP) (**17**)

Compound **16** (217.5 mg, 0.678 mmol) was suspended in trimethyl phosphate (6.8 mL) and lutidine (470.3 µL, 435.0 mg, 4.07 mmol, 6 equivalent) was added and the reaction mixture was stirred at 0 °C for approx. 20 min. Then, freshly distilled phosphoryl chloride (189.4 µL, 311.6 mg, 2.03 mmol, 3.0 equivalent) was added and the reaction was stirred at 0 °C until the starting material disappeared, as determined by RP-HPLC. Reaction was quenched by the addition of water (70 mL) and pH was neutralized with solid NaHCO_3_. The crude product was purified using ion exchange chromatography on DEAE-Sephadex (0.0–0.7 M TEAB buffer gradient) and isolated as a TEAH^+^ salt (4395 mODU, 0.364 mmol, 54%). HPLC method A: t_R_ = 5.998 min. ^1^H NMR (400 MHz, deuterium oxide) δ 8.14 (1H, s), 5.92 (1H, d, *J* = 6.1 Hz), 4.86 (2H, d, *J* = 2.3 Hz), 4.78 (1H, m, overlapped with HDO), 4.49 (1H, dd, *J* = 5.2, 3.5 Hz), 4.32 (1H, qd, *J* = 3.5, 1.5 Hz), 4.10–3.99 (2H, m), 2.73 (1H, t, *J* = 2.3 Hz), ^31^P NMR (162 MHz, deuterium oxide) δ 2.04 (1P, t, *J* = 4.9 Hz). HRMS ESI (−) *m*/*z* Calc. for C_13_H_15_N_5_O_8_P^−^ [M − H]^−^:400.06583 found: 400.06630.

#### 4.5.17. N1-Propargylguanosine 5′-*O*-monophosphate P-imidazolide (propargyl-N1-GMP-Im) (**18**)

Compound **18** was prepared according to the Mukaiyama and Hashimoto procedure [29]. Compound **17** (1.0 g, 2.5 mmol, 1 equivalent, TEAH^+^ salt), imidazole (1.70 g, 25.00 mmol, 10 equivalent), and 2,2′-dithiodipyridine (1.65 g, 7.5 mmol, 3 equivalent) were suspended in dry DMF (~1.5 mL/100 mg of nucleotide) before adding triethylamine (757.00 mg, 1.04 mL, 7.5 mmol, 3 equivalent) and triphenylphosphine (1.96 g, 7.5 mmol, 3 equivalent). The mixture was stirred for 24 h at RT. Then, the product was precipitated by the addition of a solution of anhydrous NaClO_4_ (1.22 g, 10 mmol, 4 equivalent) in dry acetone (15 mL). The precipitate was filtered off, washed repeatedly with cold, dry acetone, and dried under vacuum over P_4_O_10_ (1.07 g, 95%). HPLC method A t_R_ = 8.579 min. ^1^H NMR (400 MHz, deuterium oxide) δ 7.88 (1H, s), 7.81 (1H, t, *J* = 1.3 Hz), 7.10 (1H, q, *J* = 1.3 Hz), 6.88 (1H, m), 5.83 (1H, d, *J* = 5.3 Hz), 4.87 (2H, t, *J* = 2.2 Hz), 4.84 (1H, t, *J* = 5.3 Hz), 4.44 (1H, dd, *J* = 5.3, 4.1 Hz), 4.25 (1H, qd, *J* = 4.1, 1.8 Hz), 4.11 (2H, dd, *J* = 5.5, 4.1 Hz), 2.76 (1H, t, *J* = 2.2 Hz). ^31^P NMR (162 MHz, deuterium oxide) δ -8.00 (1P, qd, *J* = 5.5, 2.3 Hz). HRMS ESI (−) *m*/*z* Calc. for C_16_H_17_N_7_O_7_P^−^ [M − H]^−^: 450.0932 found: 450.0933.

#### 4.5.18. N1-Propargylguanosine 5′-*O*-(1,2-methylenediphosphate) (propargyl-N1-GpCH_2_p) (**22**)

Compound **16** (100 mg, 0.31 mmol) was suspended in trimethyl phosphate (1.5 mL) and stirred at 0 °C for approx. 15 min. Then methylene bis(phosphonic dichloride) (CH_2_(POCl_2_)_2_, 3 equivalent, 0.93 mmol, 230 mg) was added and the reaction was stirred at 0 °C until the starting material disappeared as determined by RP-HPLC (usually 4–5 h). Then, the reaction was quenched by the addition of 0.7 M TEAB (pH 7) or 1 M NaHCO_3_ until neutral pH was reached. Crude product was purified by DEAE-Sephadex and isolated as TEAH^+^ salts (111.70 mg, 1705 mOD, 75%). HPLC method A t_R_ = 4.801 min. ^1^H NMR (deuterium oxide, 400 MHz) δ 8.13 (1H, s), 5.93 (1H, d, *J =* 6.1 Hz), 4.88 (2H, d, *J =* 2.4 Hz), 4.83 (1H, dd, *J =* 6.1, 5.2 Hz), 4.54 (1H, dd, *J =* 5.2, 3.5 Hz), 4.34 (1H, qd, *J =* 3.7, 0.8 Hz), 4.16 (2H, dd, *J =* 5.6, 4.0 Hz), 2.73 (1H, t, *J =* 2.4 Hz), 2.17 (2H, t, *J =* 19.8 Hz). ^31^P NMR (deuterium oxide, 162 MHz) δ 18.74–18.09 (1P, m), 14.53 (1P, td, *J =* 19.9, 9.6 Hz). HRMS ESI (−) *m*/*z* Calc. for C_14_H_18_N_5_O_10_P_2_^−^ [M − H]^−^: 478.0529 found: 478.0535.

#### 4.5.19. N1-Propargylguanosine 5′-*O*-(1,2-imidodiphosphate) (propargyl-N1-GpNHp) (**23**)

Compound **16** (100 mg, 0.31 mmol) was suspended in trimethyl phosphate (1.5 mL) and stirred at 0 °C for approx. 15 min. Then, dichlorophosphorylphosphorimidoyl trichloride (Cl_3_PNP(O)Cl_2_, 3 equivalent, 0.93 mmol, 248 mg, 450 µL) was added under vigorous stirring. Reaction was stirred at 0 °C until the starting material disappeared as determined by RP-HPLC (usually 2–3 h). Then, the reaction was stopped by the addition of 0.7 M TEAB (pH 7) or 1 M NaHCO_3_ until neutral pH was reached. The crude product was purified on DEAE Sephadex and isolated as TEAH^+^ salt (96.8 mg, 1425 mOD, 63%). HPLC method A t_R_ = 4.412 min. ^1^H NMR (deuterium oxide, 400 MHz) δ 8.14 (1H, s), 5.92 (1H, d, *J =* 6.1 Hz), 4.87 (2H, dd, *J =* 2.6, 1.1 Hz), 4.82 (2H, t, *J =* 6.1, 5.3 Hz), 4.56 (1H, dd, *J =* 5.3, 3.5 Hz), 4.35 (1H, qd, *J =* 3.5, 1.7 Hz), 4.14 (2H, dd, *J =* 5.5, 3.6 Hz), 3.20 (17H, q, *J =* 7.3 Hz), 2.73 (1H, t, *J =* 2.5 Hz), 1.27 (27H, t, *J =* 7.3 Hz), ^31^P NMR (deuterium oxide, 162 MHz) δ 0.98 (1P, q, *J* = 5.5 Hz), −0.59 (1P, d, *J* = 5.5 Hz). HRMS ESI (−) *m*/*z* Calc. for C_13_H_17_N_6_O_10_P_2_^−^: 479.0481 found: 479.0489.

### 4.6. Determination of the eIF4E–Cap Complex Dissociation Constants K_D_

A previously described pyrene fluorescence intensity binding method was used to determine the dissociation constants of the nonfluorescent ligands binding to the eIF4E protein in a competitive binding experiment [16]. The experiments were performed in 96-well, black, non-binding assay plates with point fluorescence measurements (*λ_exc_* = 345 nm; *λ_em_* = 378 nm). Each well contained a buffer (50 mM Hepes/KOH pH = 7.2 containing 100 mM KCl and 0.5 mM EDTA), 10 nM of pyrene-labeled 7-methylguanosine pentaphosphate probe, ligand **1**–**5** or m^7^Gp_3_G (half-log dilutions of C_lig_ from 100 μM to 0.003 μM), and 75 nM of eIF4E protein. The reagents and added protein were pre-incubated for 15 min at 30 °C and stirred at 300 rpm. Measurements were performed in three different temperatures: At 20, 30, and 37 °C. A previously derived Equation (1) [35] was used to calculate the inhibition (dissociation) constant *K*_I_ of a competitive ligand according to the dependence of the recorded fluorescence intensity on the inhibitor concentration.

(1) $$K_{I}=\frac{IC_{50}-\left[P_{t}\right]+{\left[PL\right]}_{50}\left(\frac{K_{D}}{{\left[L\right]}_{50}}+1\right)}{\frac{{\left[L\right]}_{50}}{K_{D}}+\frac{{\left[P\right]}_{0}}{K_{D}}+1}$$

where *IC*_50_ is the inhibitor concentration required to replace 50% of the fluorescent probe from the protein binding site, [*P_t_*] is the total protein concentration, [*PL*]_50_ is the protein–probe complex concentration at 50% inhibition, [*L*]_50_ is the free probe concentration at 50% inhibition, and [*P*]_0_ is the free protein concentration without inhibition.

In order to determine IC_50_ value, we used Origin^®^ 2017 (OriginLab Corp., Northampton, MA, USA) software to fit the curve described by the derived Equation (2) to the measured fluorescence intensity values for various inhibitor concentrations.

(2) $$F=F_{0}\frac{F_{max}-F_{0}}{1+10^{\left(log\left(IC_{50}\right)-log\left(C_{lig.}\right)\right)p}}$$

where *C_lig._* is the concentration of non-fluorescent ligand.

The plots were created in GraphPad Prism v. 7.00 (GraphPad Software, La Jolla, CA, USA).

### 4.7. Susceptibility to DcpS Hydrolysis

Enzymatic reactions with human DcpS were carried out in 50 mM Tris/HCl, 200 mM KCl, 0.5 mM EDTA, pH = 7.6 buffer at 30 °C for 30 nM hDcpS and 30 µM of a cap analog studied. As a control, m^7^Gp_3_G was used. Cap analogs concentration was determined spectrophotometrically (absorption coefficient used in calculations is equal to 22,600 mL/mmol/cm in 0.1 M phosphate buffer pH = 7.0). For **1** and m^7^Gp_3_G, 3 independent experiments were performed. Aliquots were terminated by heat inactivation for 3 min at 95 °C. Samples were analyzed with RP-HPLC using method E.

### 4.8. UV-Vis and Fluorescence Measurements

Absorption spectra were recorded with a Cary 100 UV-Vis (Agilent Technologies, Santa Clara, CA, USA) and a Dual Cell Peltier holder spectrophotometer at 25 °C. Absorption spectra were recorded in 0.1 M NaOH for probes containing fluorescein **(6, 11**–**14)** or 50 mM Tris/HCl, 200 mM KCl, 0.5 mM EDTA, pH = 7.6 for all others (**7**–**9**). Emission and excitation spectra were recorded with a Cary Eclipse (Agilent Technologies, Santa Clara, CA, USA) equipped with a xenon lamp under thermostatic conditions in a quartz cuvette (10 mm × 4 mm) in 50 mM Tris/HCl, 200 mM KCl, 0.5 mM EDTA, pH = 7.6 at 25 °C.

### 4.9. hDcpS and SVPDE Hydrolysis Monitoring

Human DcpS was expressed and purified as described previously [16]. Enzyme was stored at −80 °C at 10 µM concentration (monomer). Enzyme hDcpS was preincubated at 30 °C for 15 min before usage. Enzymatic reactions with hDcpS enzyme were conducted using 30 nM hDcpS (dimer) and 100 nM compound in 50 mM Tris/HCl, 200 mM KCl, 0.5 mM EDTA, pH = 7.6 buffer at 30 °C. Reaction progress was monitored by recording emission spectra, upon excitation at the wavelength characteristic for a fluorophore studied, using Cary Eclipse (Agilent Technologies, Santa Clara, CA, USA) equipped with a xenon lamp in a quartz cuvette (10 mm × 4 mm).

PDE-I from *Crotalus adamanteus* (EC 3.1.4.1) venom was purchased as a lyophilized solid from Sigma-Aldrich Corp. (St. Louis, MO, USA). The PDE-I was dissolved in a storage buffer (110 mM Tris/HCl pH 8.9 buffer containing 110 mM NaCl, 15 mM MgCl_2_, and 50% glycerol) to prepare a 1 mg/mL solution and then stored at −20 °C. Before the assay, the enzyme was diluted to 100 µg/mL with 50 mM Tris/HCl, pH = 8.0 buffer. Enzymatic reactions with PDE-I were conducted for 100 ng/mL and 100 nM compound in 50 mM Tris/HCl, 5 mM MgCl_2_, pH = 8.0 buffer at 30 °C. Reaction progress was monitored by recording the emission spectra at the excitation wavelength of a fluorophore in a Cary Eclipse (Agilent Technologies, Santa Clara, CA, USA) equipped with xenon lamp using a quartz cuvette 10 × 4 mm.

### 4.10. Microscale Thermophoresis Direct Binding Affinity for meIF4E and hDcps

Probes **6**, **11**, and **12** were dissolved in MST buffer (50 mM HEPES/KOH, 100 mM KCl, 0.5 mM EDTA, pH = 7.2, 0.2% Tween 20) to obtain a 50 nM stock solution. Murine eIF4E (meIF4E) was dissolved in MST buffer in a 16 point, 1:1 dilution series in a 10.0 µM to 0.30 nM concentration range. Equal volumes (10 µL) of meIF4E and probe were combined to obtain the following assay concentrations: 25 nM probe and 5.0 µM to 0.15 nM meIF4E for the assay.

Probes **11** and **12** were dissolved in MST buffer (50 mM HEPES/KOH, 100 mM KCl, 0.5 mM EDTA, pH = 7.2, 0.2% Tween 20) to obtain a 50 nM stock solution. In study of a probe **11**, human DcpS (hDcpS) was dissolved in MST buffer in a 16 point 1:1 dilution series in a 2.0 µM to 0.061 nM concentration range. Equal volumes (10 µL) of hDcpS and the probe were mixed together to obtain the following assay concentrations: 25 nM probe and 1.0 µM to 0.030 nM hDcpS. In study of a probe **12**, hDcpS was dissolved in MST buffer in a 16 point 2:1 dilution series in a 700 nM to 1.60 nM concentration range. Equal volumes (10 µL) of hDcpS and the probe were mixed together to obtain the following assay concentrations: 25 nM probe and 350 nM to 0.80 nM hDcpS.

For both assays, samples were loaded into standard Monolith™ NT.115 Series Capillares (NanoTemper Technologies, Cambridge, MA, USA). MST measurements were performed on Monolith NT.115 instrument (NanoTemper Technologies, Cambridge, MA, USA) at 25 °C. Measurements parameters were set as follows: 40% LED Blue power, Medium MST power. Data were obtained for three independently pipetted measurements and analyzed in PALMIST v. 1.4.4 [36] using a 1:1 binding model to calculate *K*_D_, using the signal from an MST-on time of 20 s (for eIF4E assay: Cold region start, −3 s; hot region start, 0.5 s; for DcpS assay: Cold region start, −3 s; hot region start 19 s), confidence intervals were calculated with error surface projection (ESP) method. Plots presented were generated using GUSSI v. 1.4.2 [37].

## Figures and Tables

**Table 1:** IC_50_ and *K*_D_ values determined at 30 °C for the binding affinity for murine eIF4E. The IC_50_ values were determined from the data shown in Figure 2. The *K*_D_ values were calculated from the IC_50_ values using a previously reported equation [33].

Compound	IC_50_ (µM)	*K*_D_ (nM)
**1**	3.57 ± 0.25	305 ± 45
**2**	6.37 ± 0.73	548 ± 95
**3**	3.48 ± 0.22	297 ± 43
**4**	6.04 ± 0.46	520 ± 78
**5**	2.16 ± 0.20	182 ± 29
m^7^Gp_3_G	3.46 ± 0.30	294 ± 46

**Table 2:** *K*_D_ values of the binding affinities for murine eIF4E and human DcpS determined by MST. *K*_D_ values were calculated from triplicate experiments.

Compound	meIF4E		hDcpS	
	*K*_D_ [nM]	Confidence Interval	*K*_D_ [nM]	Confidence Interval
**6**	50	(43, 58)	n.d.	n.d.
**11**	201	(184, 220)	38	(28, 51)
**12**	53	(46, 62)	14	(7, 24)

## Supplementary Materials

The supplementary materials are available online.
