# Supplementary material for: N1-Propargylguanosine Modified mRNA Cap Analogs: Synthesis, Reactivity, and Applications to the Study of Cap-Binding Proteins
Source: Molecules. 2019 May 17;24(10):1899. doi: 10.3390/molecules24101899 (PMC6572376; doi:10.3390/molecules24101899)

N1-propargylguanosine modified mRNA cap analogs: synthesis, reactivity, and applications to the study of cap-binding proteins

Michal Kopcial[1,2,3], Blazej A. Wojtczak[2], Renata Kasprzyk[1,2,3], Joanna Kowalska[3] and Jacek Jemielity[2*]

[1] College of Inter-Faculty Individual Studies in Mathematics and Natural Sciences, University of Warsaw
[2] Centre of New Technologies, University of Warsaw
[3] Faculty of Physics, University of Warsaw
* Correspondence: j.jemielity@cent.uw.edu.pl; Tel.: +48 22 55 43 774, Centre of New Technologies, University of Warsaw, S. Banacha 2c, 02-097, Warsaw

Supporting Information (SI2)

Tables 2
Table S1. Murine eIF4E binding affinity assay IC50 and KD values. 2
Table S2. Half-life for m^7Gp_3G and compound **4** 2
Figures 3
Figure S1. Murine eIF4E binding affinity assay 3
Figure S2. Absorption, emission and excitation spectra of probes 16a-d, 17a-20a 4
Figure S3. Probe **16a-d** hydrolysis by hDcpS enzyme monitored by emission spectroscopy 5
Figure S4. Probe **16a-d** hydrolysis by PDE-I enzyme monitored by emission spectroscopy 6
Figure S5. Probe binding affinity for meIF4E measured by microscale thermophoresis 7
Figure S6. Probes affinity binding with hDcpS measured by microscale thermophoresis 7
Compound characterization 8
m^7Gp_3G-N1-propargyl **(1)** 8
m^7GpCH_2ppG-N1-pr **(2)** 9
$m^7GpNHppG$-N1-propargyl **(3)** 11
m^7GppCH_2pG-N1-propargyl **(4)** 13
$m^7GppNHpG$-N1-propargyl **(5)** 15
m^7Gp_3G-N1-5FAM **(6)** 17
m^7Gp_3G-N1-Cy3 **(7)** 19
m^7Gp_3G-N1-Cy5 **(8)** 21
m^7Gp_3G-N1-Py **(9)** 23
m^7Gp_3G-N1-biotin **(10)** 25

m^7GpCH_2ppG-N1-5FAM **(11)** 26

m^7GpNHppG-N1-5FAM **(12)** 28

m^7GppCH_2pG-N1-5FAM **(13)** 30

m^7GppNHpG-N1-5FAM **(14)** 32

propargyl-N1-Guo **(16)** 34

propargyl-N1-GMP **(17)** 35

propargyl-N1-GMP-Im **(18)** 37

propargyl-N1-$GpCH_2p$ **(22)** 39

propargyl-N1-GpNHp **(23)** 40

Tables

Table S1. Murine eIF4E binding affinity assay IC_{50} and K_D values.

Compound	IC_{50} (µM)		
	20°C	30°C	37°C
**1**	2.92 ± 0.19	3.57 ± 0.25	3.68 ± 0.70
**2**	6.57 ± 0.49	6.37 ± 0.73	8.20 ± 0.79
**3**	3.06 ± 0.16	3.48 ± 0.22	3.75 ± 0.89
**4**	5.51 ± 0.33	6.04 ± 0.46	8.10 ± 0.61
**5**	1.85 ± 0.15	2.16 ± 0.20	3.21 ± 0.33
m^7Gp_3G	3.04 ± 0.26	3.46 ± 0.30	3.31 ± 0.87
Compound	K_D (µM)		
	20°C	30°C	37°C
**1**	0.268 ± 0.040	0.305 ± 0.045	0.371 ± 0.082
**2**	0.610 ± 0.094	0.548 ± 0.095	0.836 ± 0.121
**3**	0.281 ± 0.041	0.297 ± 0.043	0.379 ± 0.100
**4**	0.511 ± 0.075	0.520 ± 0.078	0.826 ± 0.109
**5**	0.168 ± 0.026	0.182 ± 0.029	0.324 ± 0.048
m^7Gp_3G	0.279 ± 0.045	0.294 ± 0.046	0.334 ± 0.096

Table S2. Half-life for m^7Gp_3G and compound **4**

Compound	m^7Gp_3G	**1**
Half-life [min]	5.703	15.75

Figures

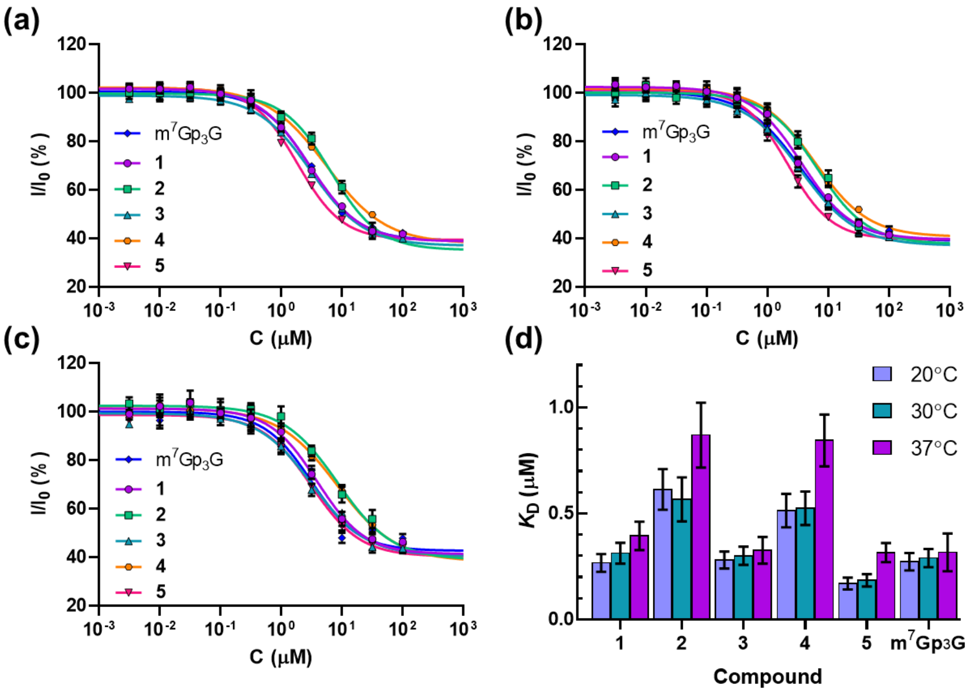

Figure S1. Murine eIF4E binding affinity assay

Competitive binding of five eIF4E ligands **1-5** and m^{7}Gp$_3$G as a control at three different temperatures (**(a)** 20°C, **(b)** 30°C and **(c)** 37°C), using previously described pyrene fluorescence intensity binding assay [1]; **(d)** comparison of calculated K_D values. Data shown are average values ± SD of 3 independent experiments.

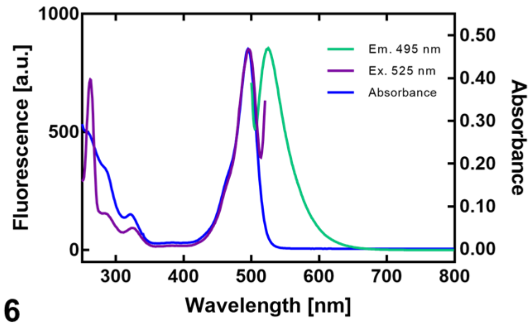

6

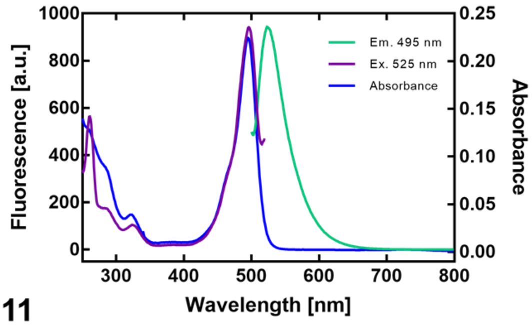

11

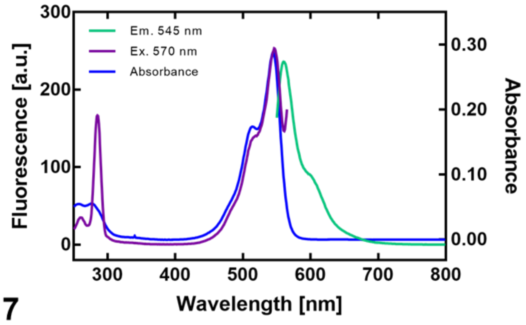

7

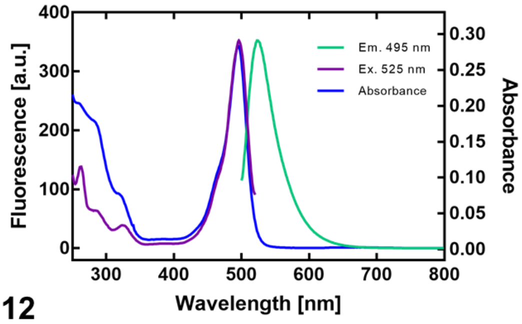

12

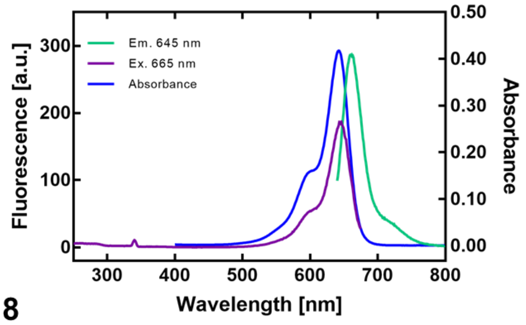

8

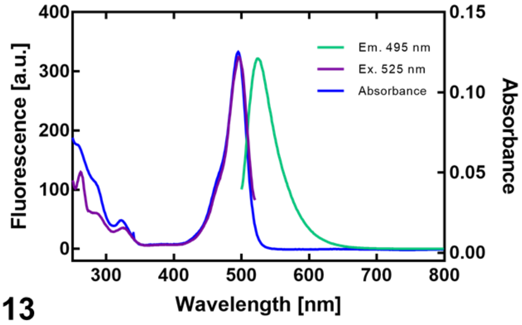

13

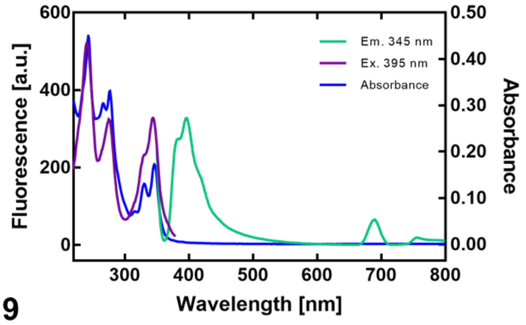

9

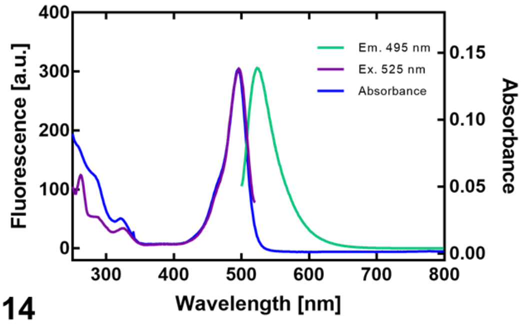

14

Figure S2. Absorption, emission and excitation spectra of probes 16a-d, 17a-20a.

Absorption spectra were recorded in 0.1 M NaOH for probes containing fluorescein (**16a-20a**) or 50 mM Tris/HCl, 200 mM KCl, 0.5 mM EDTA, pH=7.6 for all others (**16b-d**). Emission and excitation

spectra were recorded in 50 mM Tris/HCl, 200 mM KCl, 0.5 mM EDTA, pH=7.6 for 100 nM compound (except 200 nM for **16d**).

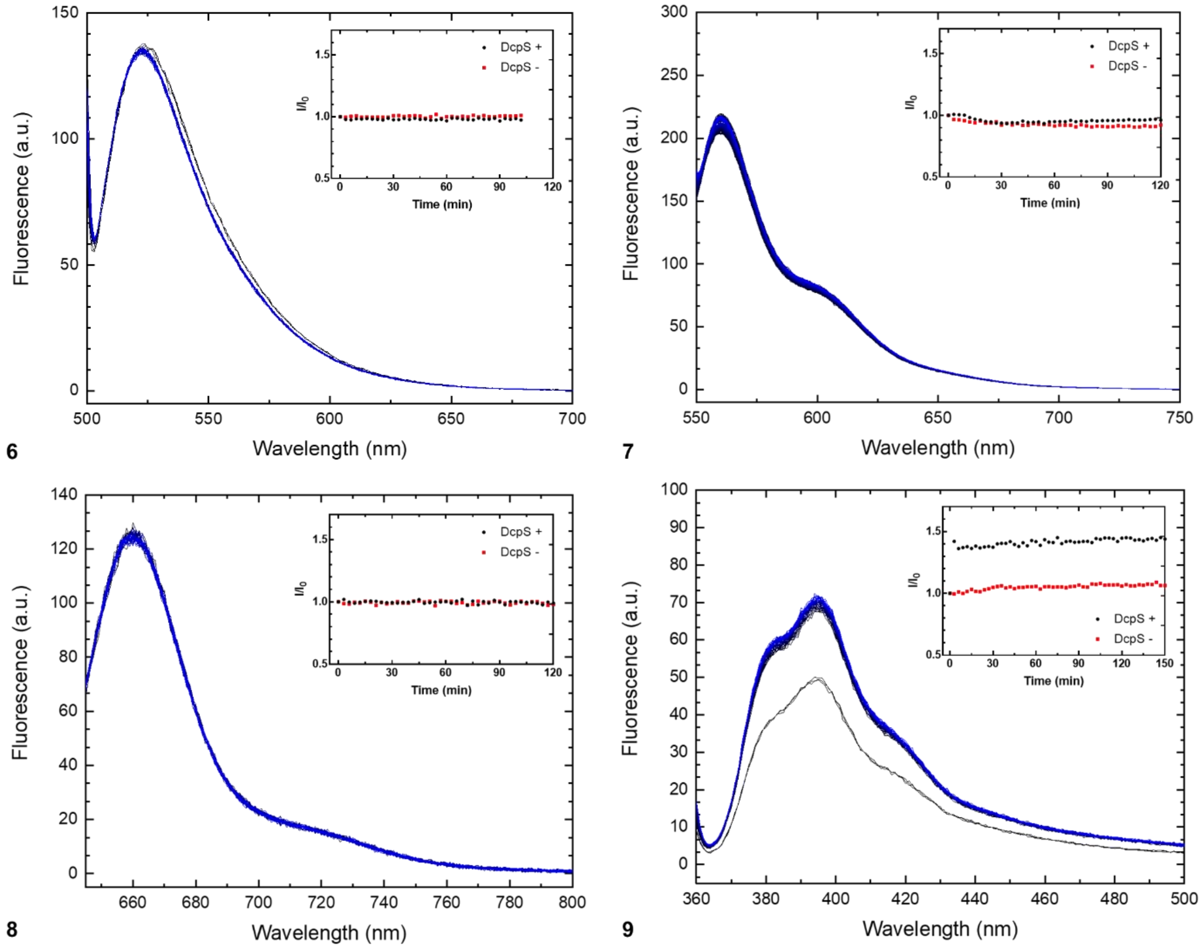

Figure S3. Probe **16a-d** hydrolysis by hDcpS enzyme monitored by emission spectroscopy

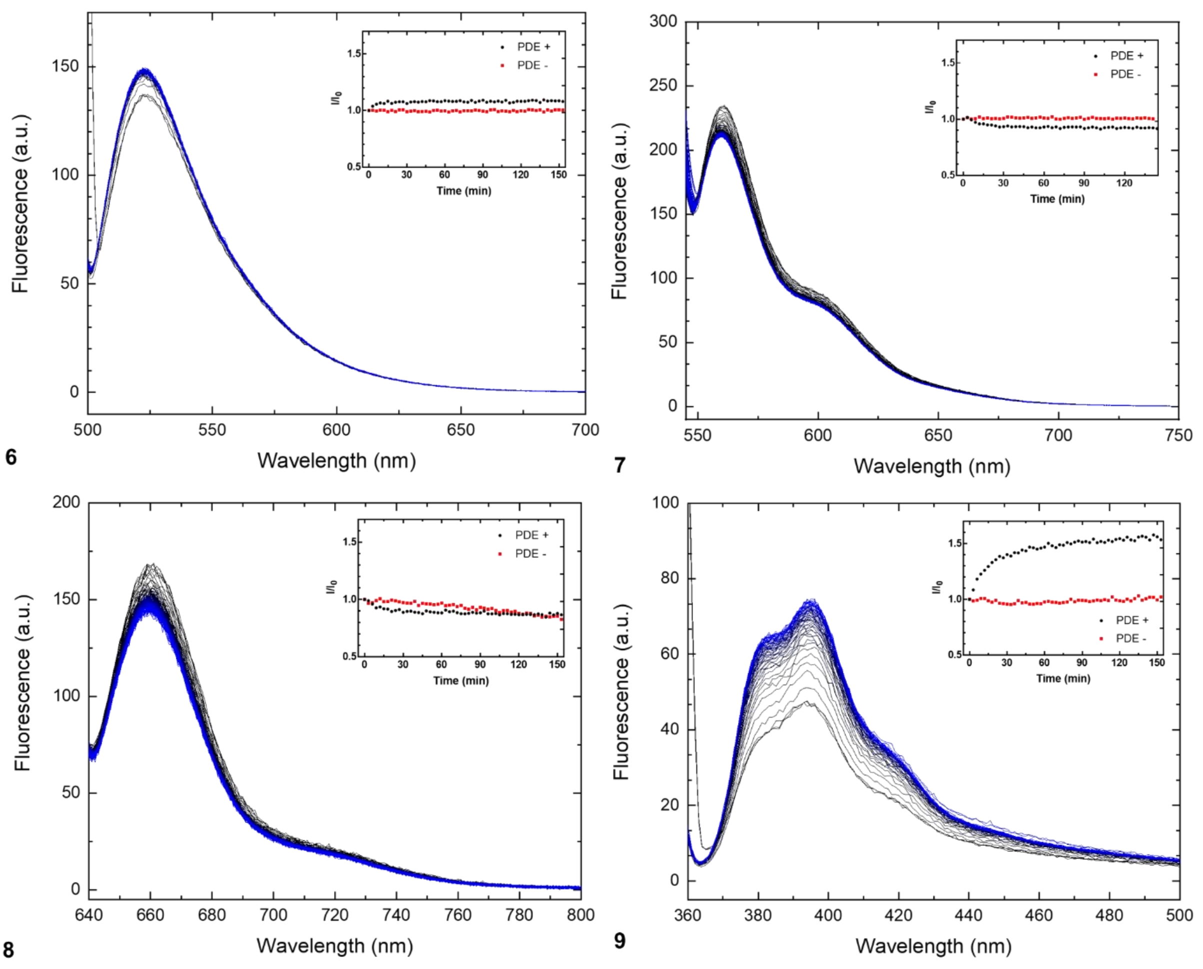

Figure S4. Probe **16a-d** hydrolysis by PDE-I enzyme monitored by emission spectroscopy

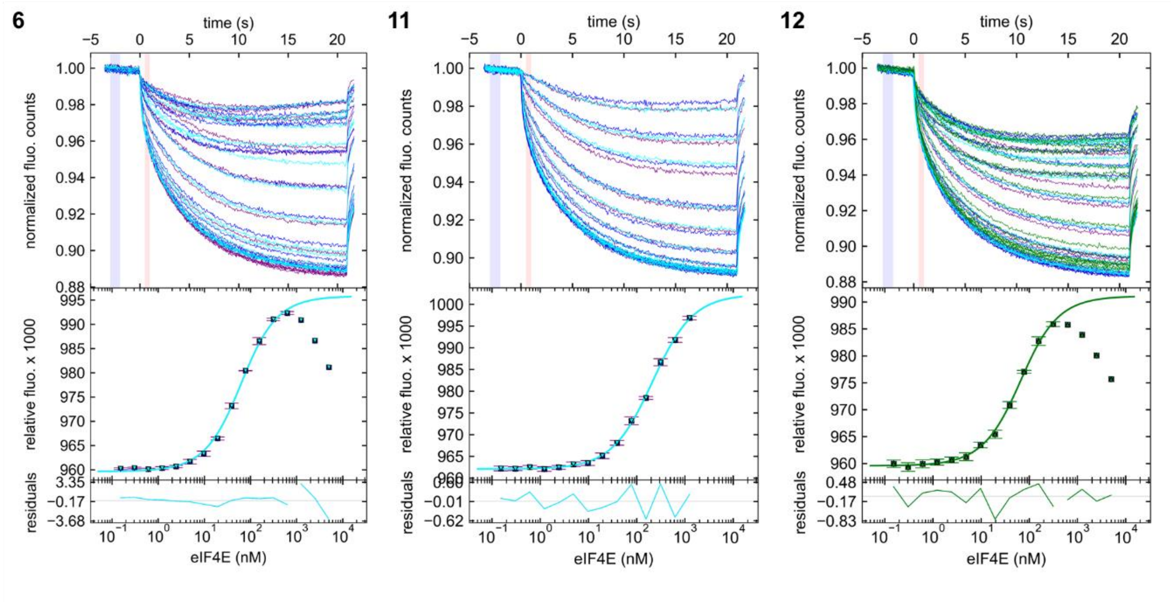

Figure S5. Probe binding affinity for meIF4E measured by microscale thermophoresis

Binding affinities of compounds **6, 11** and **12** for meIF4E determined by MST measurements. Three independent measurements were conducted (except for **12**, n=4), error bars represent standard deviation.

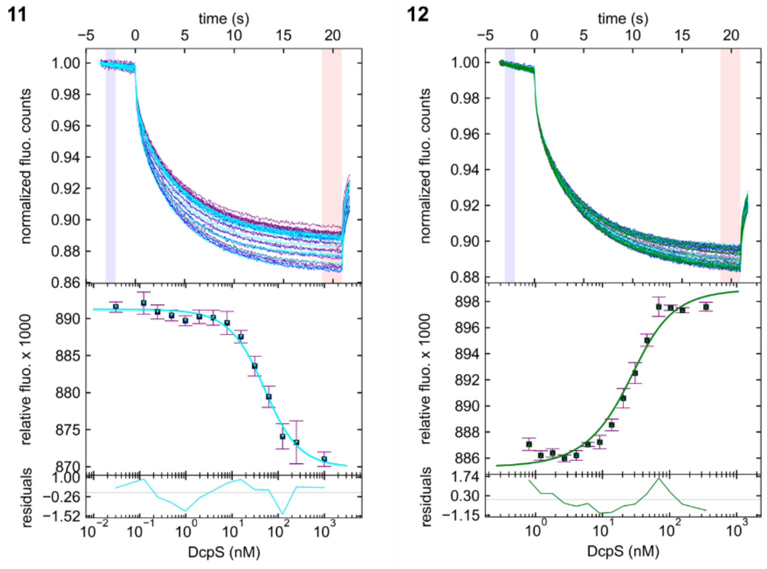

Figure S6. Probes affinity binding with hDcpS measured by microscale thermophoresis

Binding affinity of compounds **11** and **12** for hDcpS determined by MST measurements. Three independent measurements were conducted (except four for **12**, n=4), error bars represent standard deviation.

Compound characterization

<table>
<tr><th colspan="2">m7Gp3G-N1-propargyl (1)</th></tr>
<tr><td>Structure</td><td>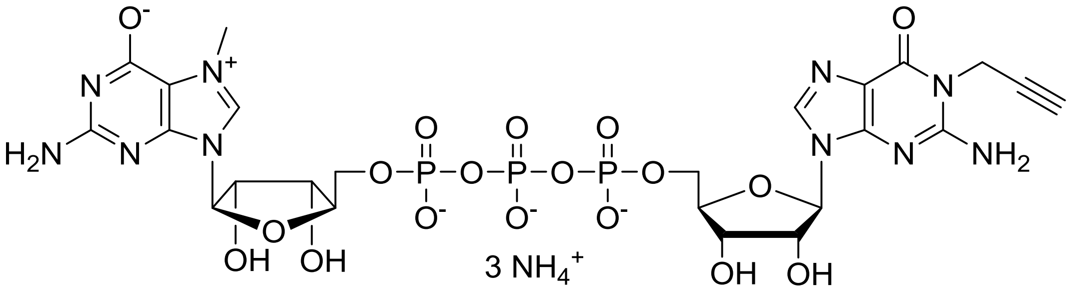
</td></tr>
<tr><td>HPLC of purified product (method A)</td><td>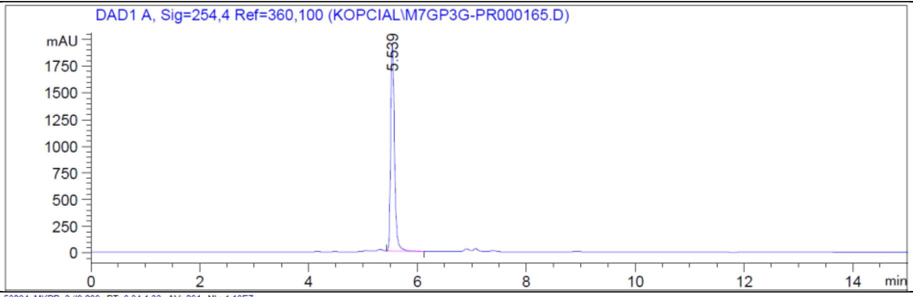
</td></tr>
<tr><td>HRMS</td><td>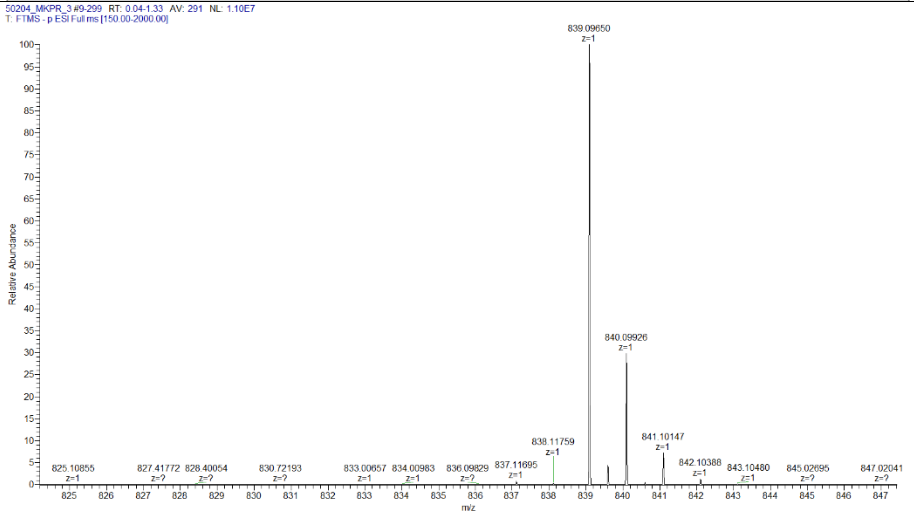
</td></tr>
</table>

^{1}H NMR (400 MHz, D_2O)

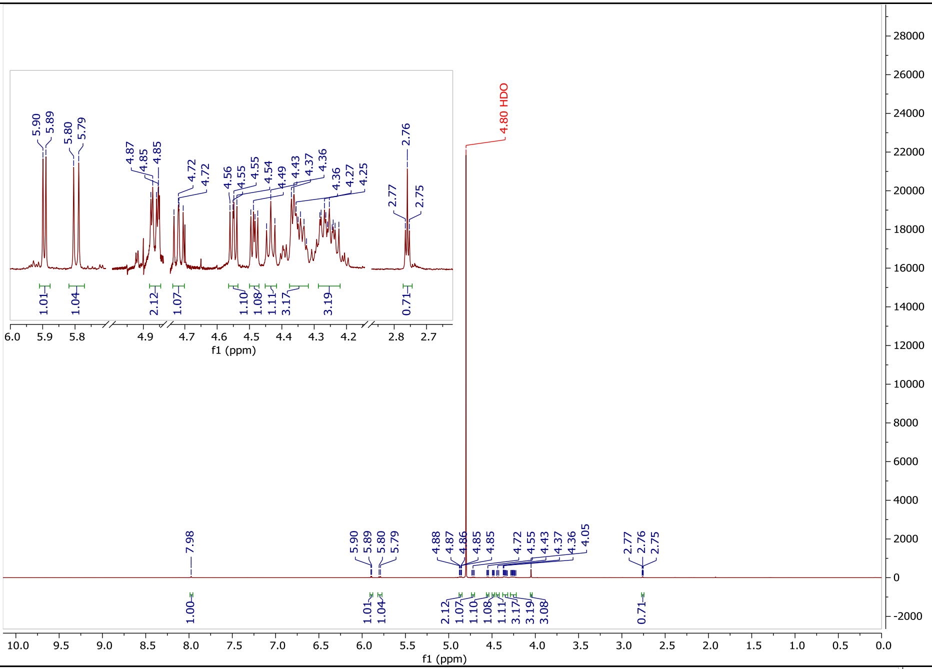

^{31}P NMR (162 MHz, D_2O)

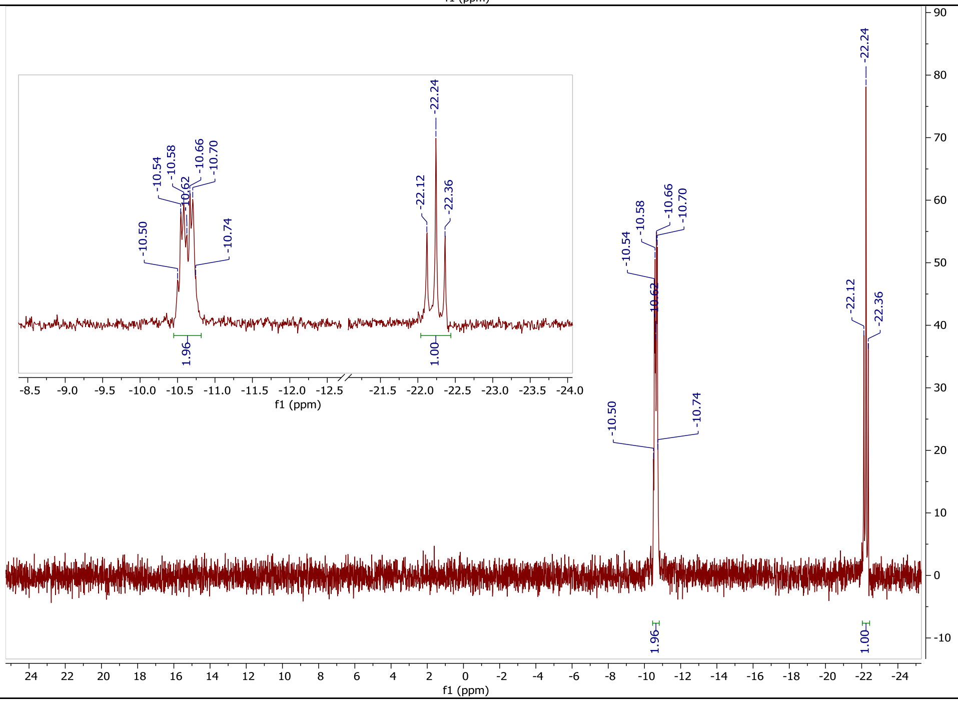

m^{7}GpCH$_2$ppG-N1-pr **(2)**

Structure

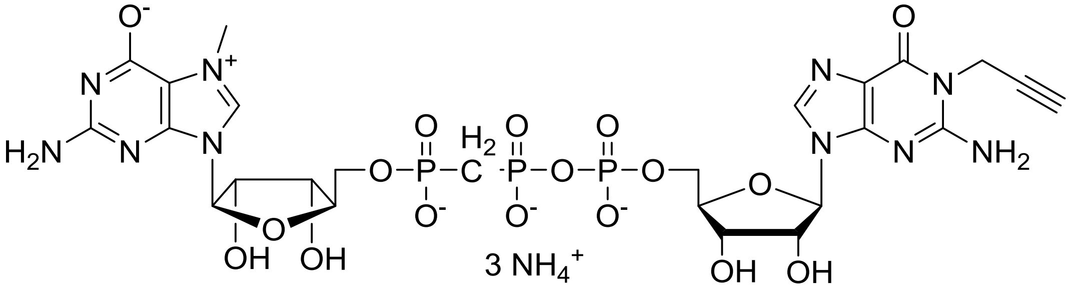

HPLC of purified product (method A)

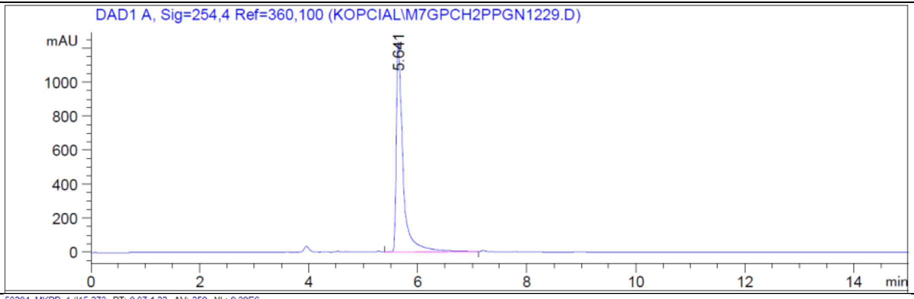

HRMS

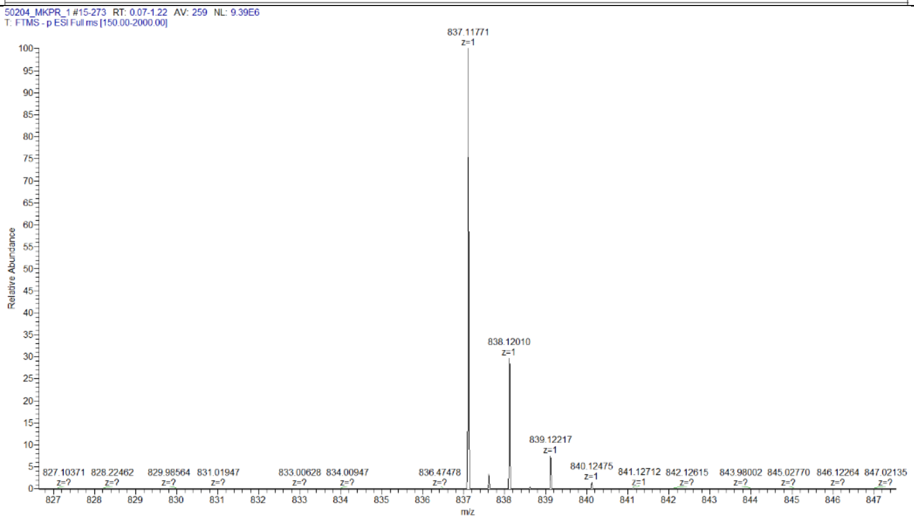

^1H NMR (400 MHz, D_2O)

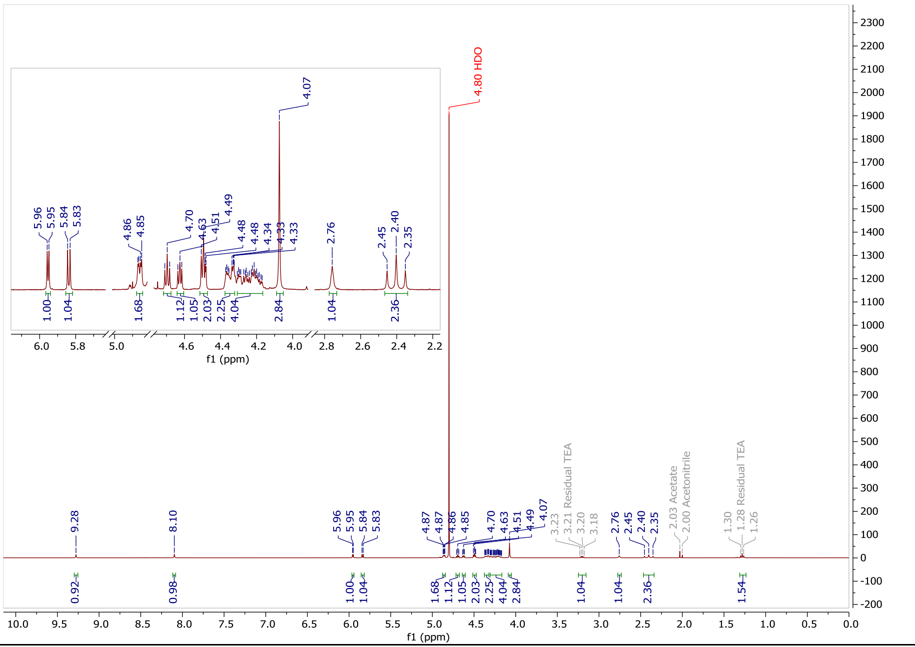

^{31}P NMR (162 MHz, D_2O)	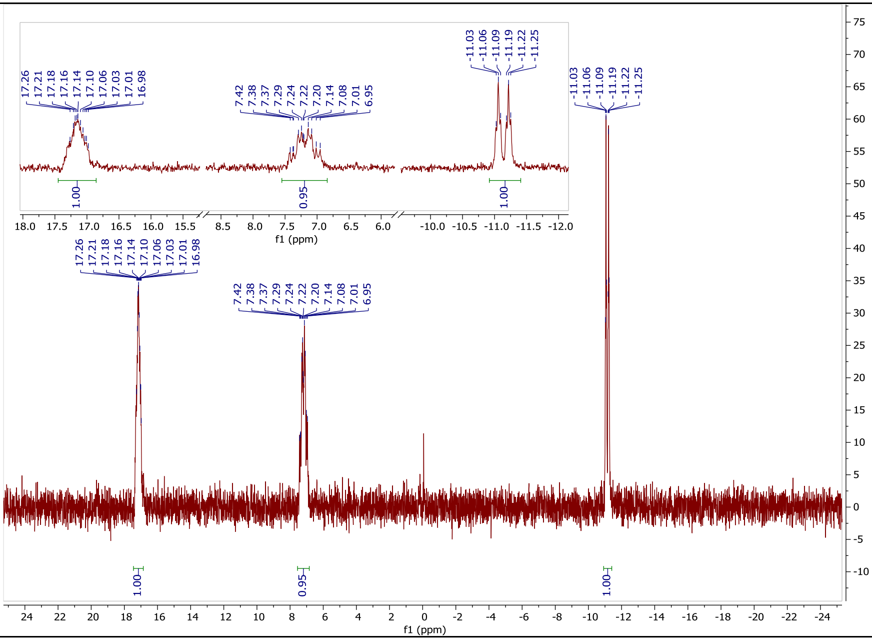

m^{7}GpNHppG-N1-propargyl **(3)**	
Structure	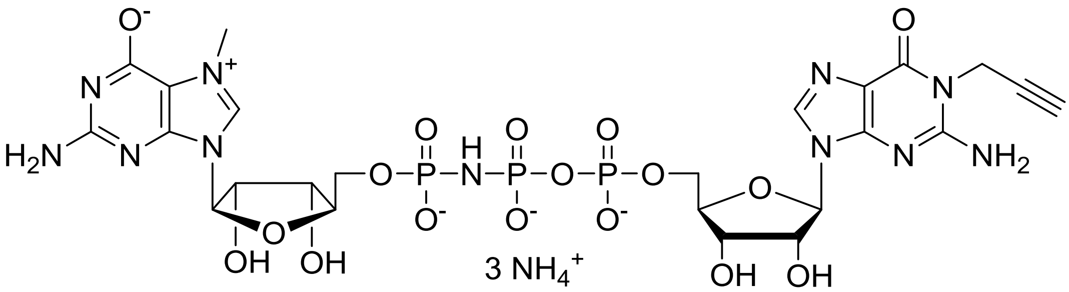
HPLC of purified product (method A)	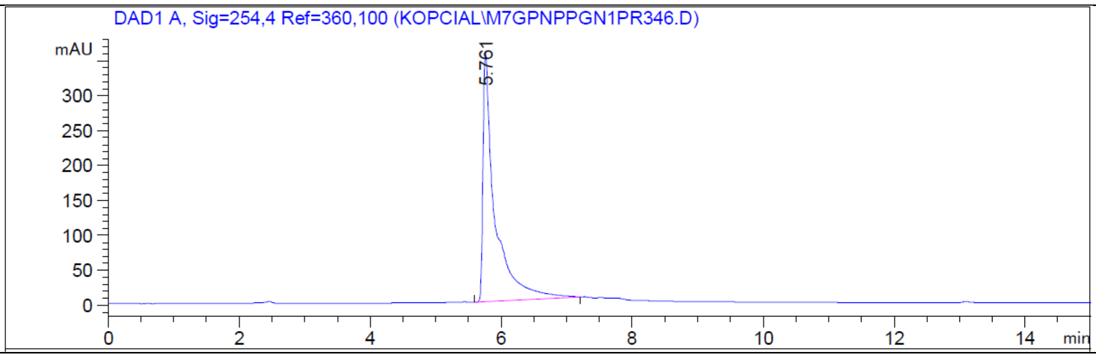

HRMS

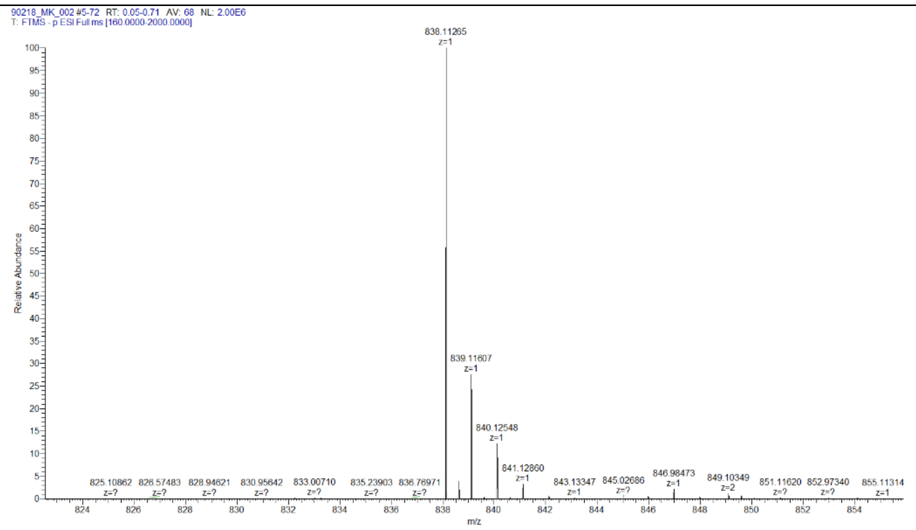

^{1}H NMR (400 MHz, D_2O)

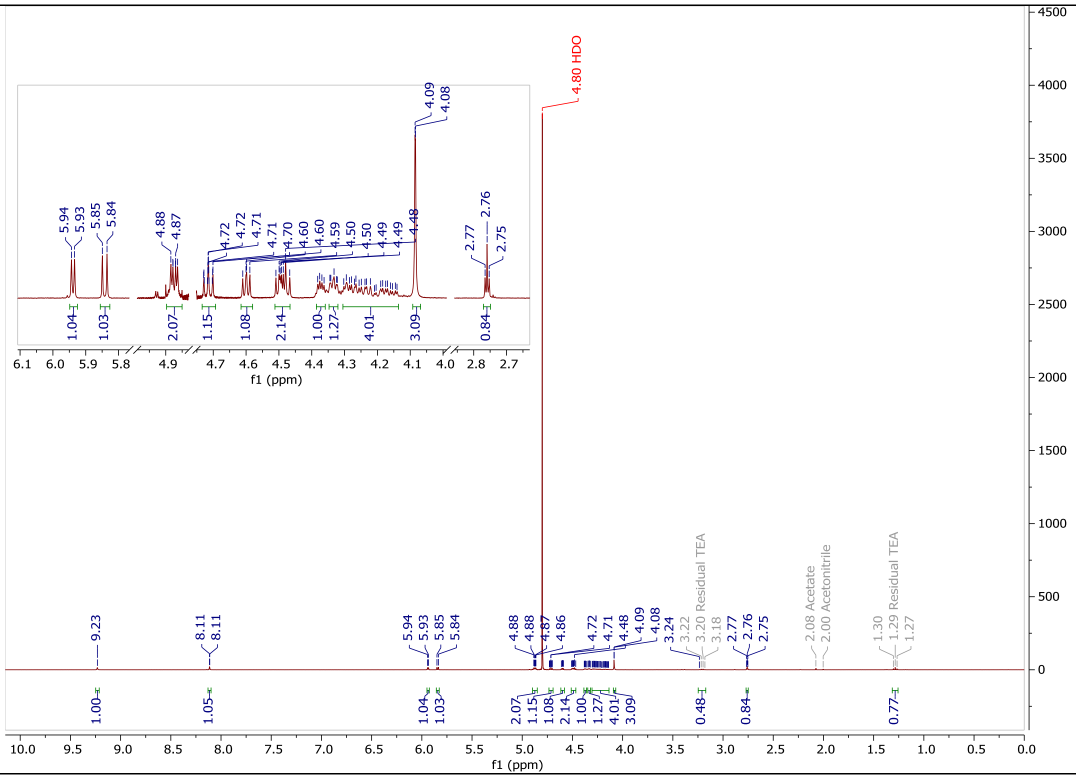

^{31}P NMR (162 MHz, D_2O)	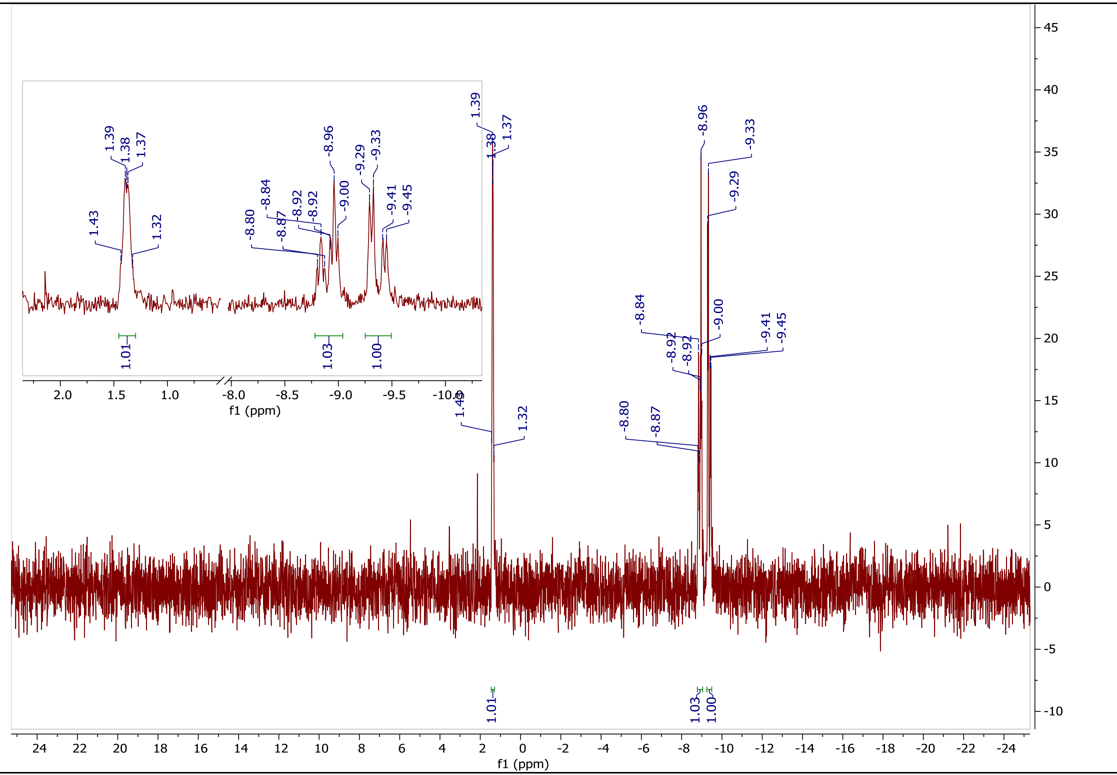

m^{7}GppCH$_2$pG-N1-propargyl **(4)**	
Structure	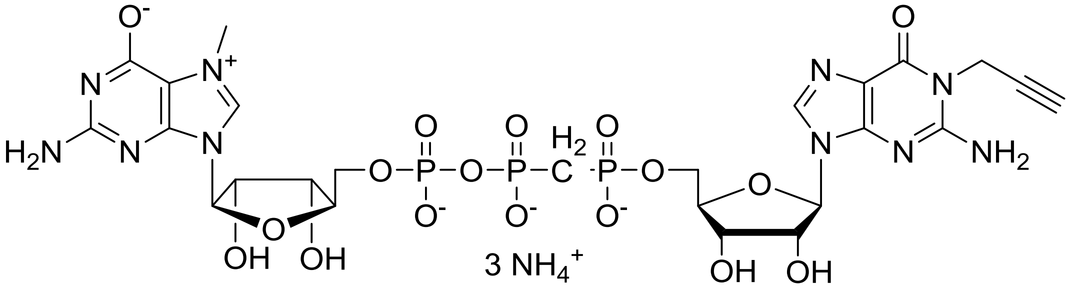
HPLC of purified product (method A)	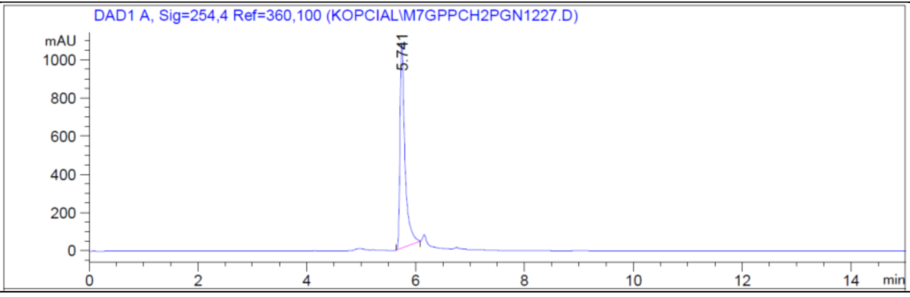

HRMS	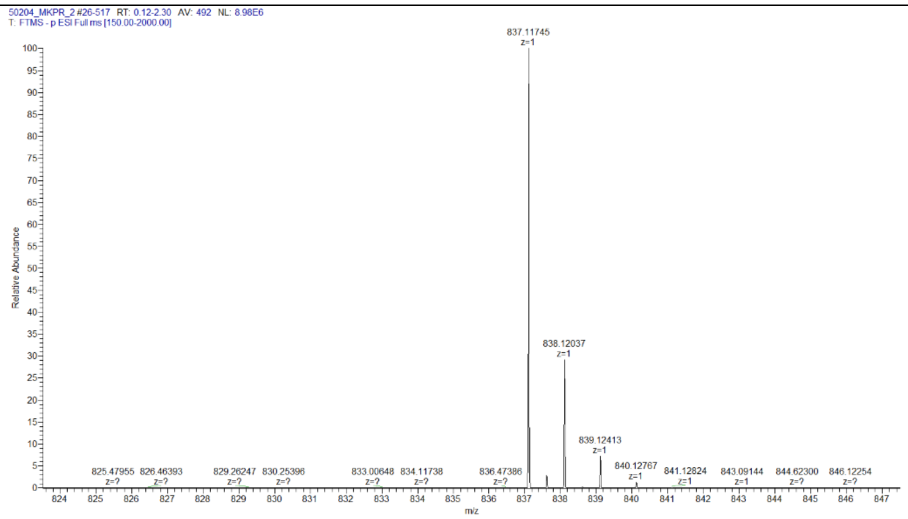
^1H NMR (400 MHz, D_2O)	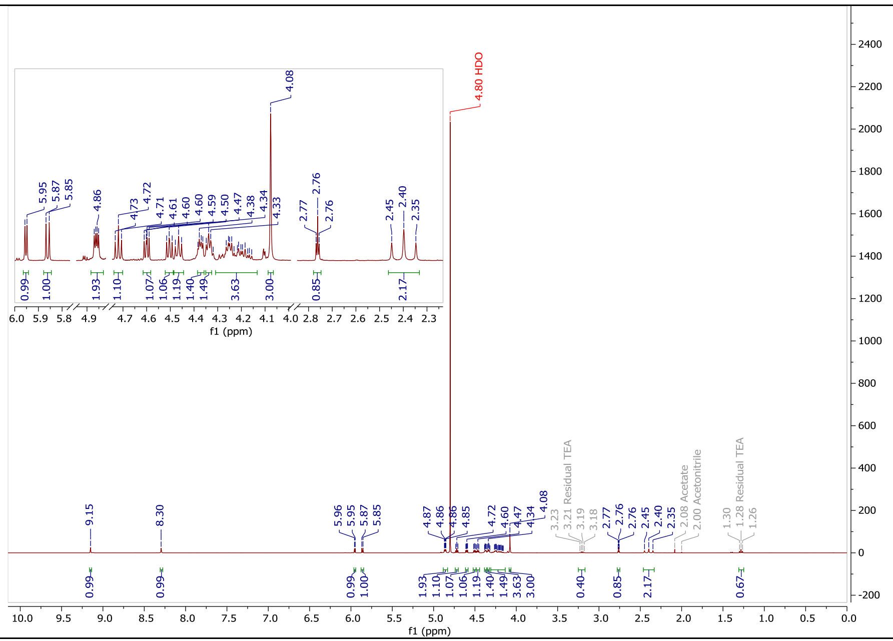

^{31}P NMR (162 MHz, D$_2$O)

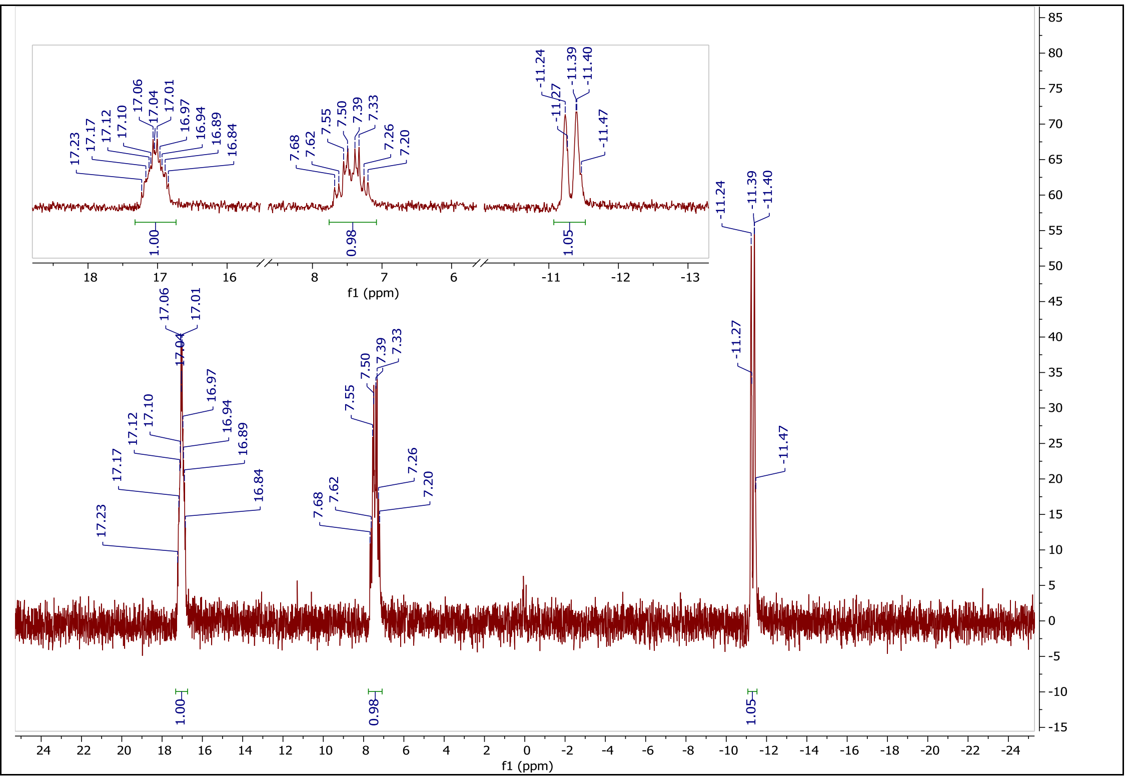

m^{7}GppNHpG-N1-propargyl **(5)**

Structure

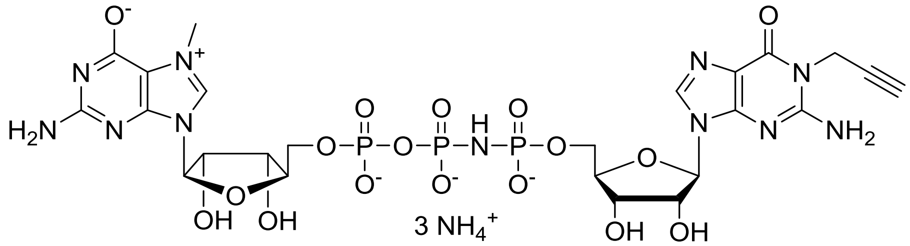

HPLC of purified product (method A)

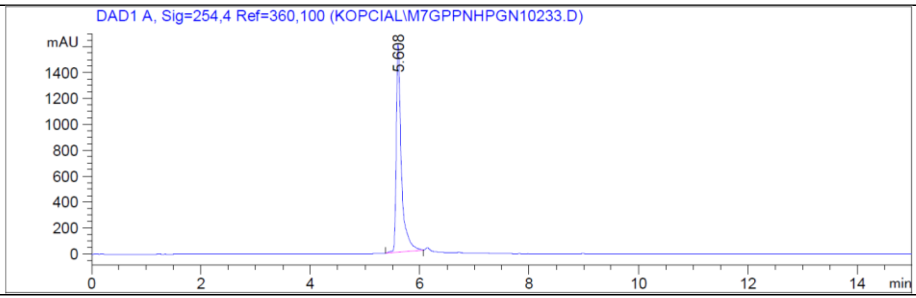

HRMS

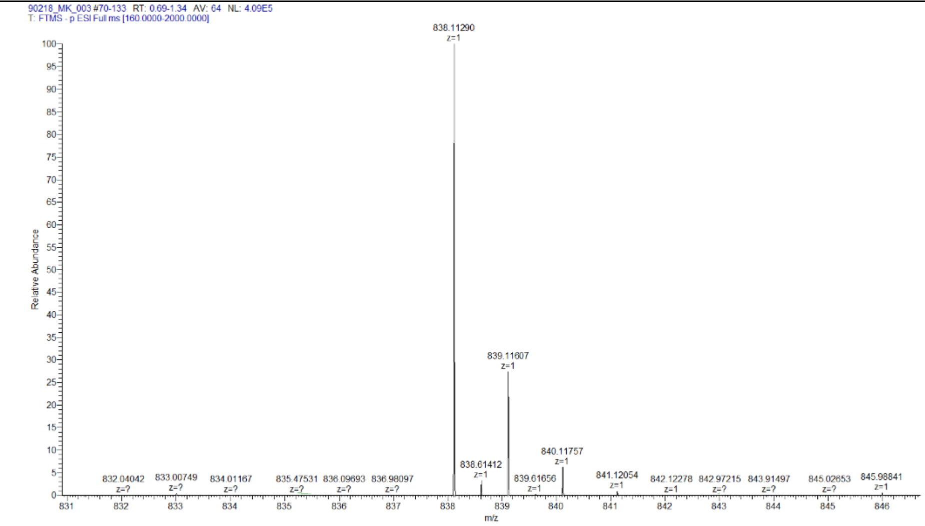

^{1}H NMR (400 MHz, D_2O)

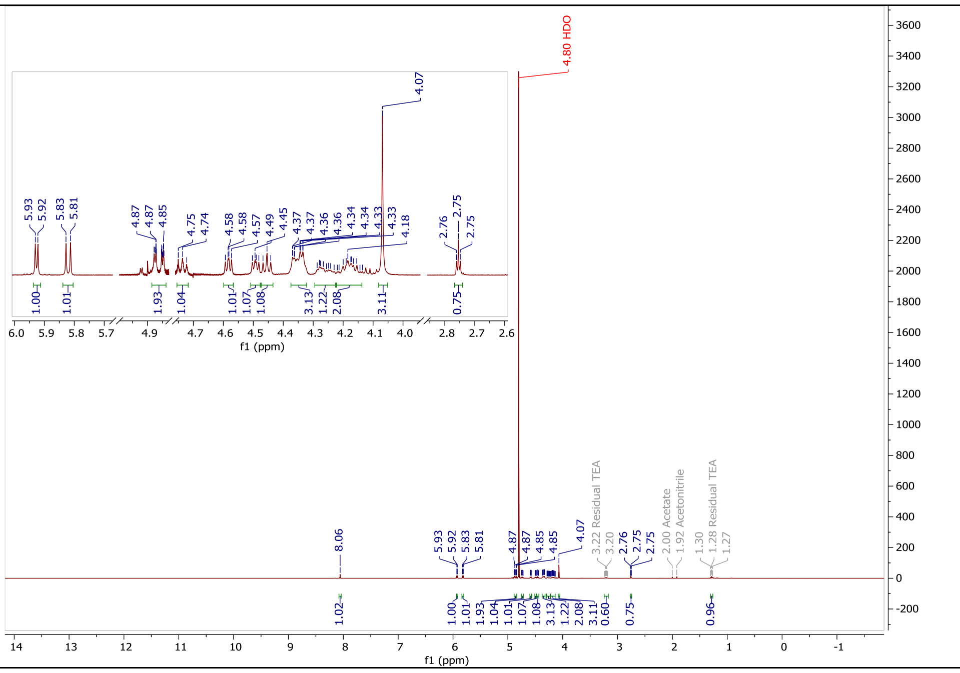

^{31}P NMR (162 MHz, D_2O)	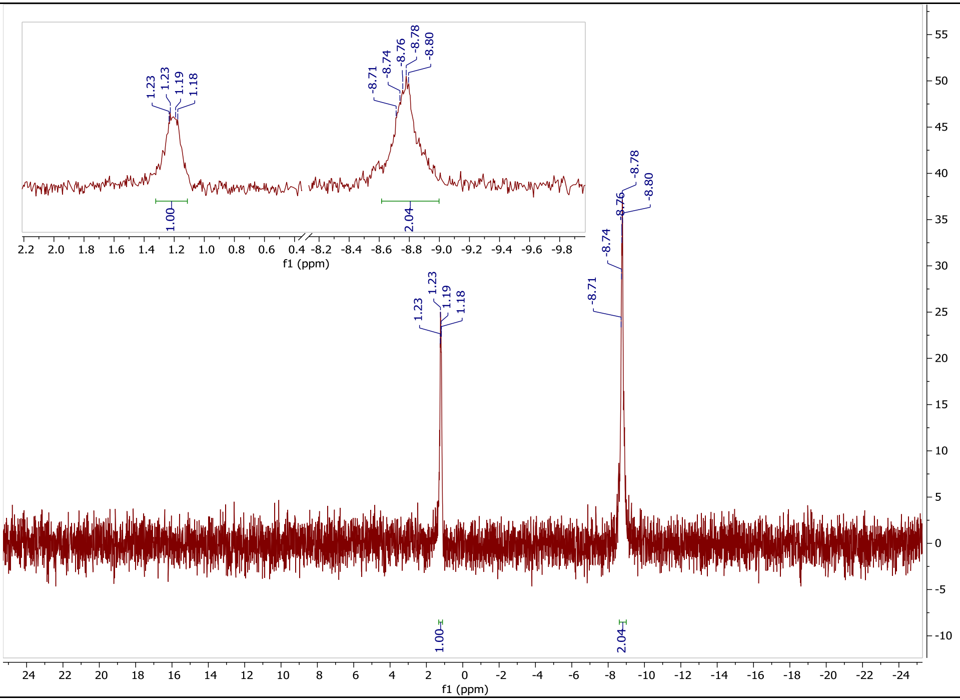

m^{7}Gp$_{3}$G-N1-5FAM **(6)**	
Structure	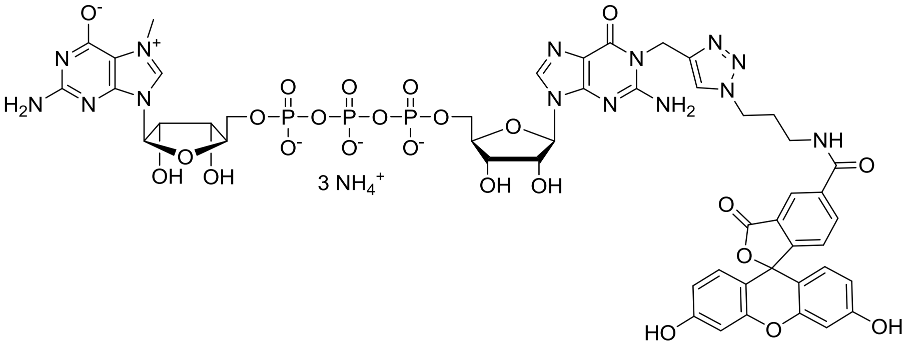

Reaction HPLC (Method C)

t = 0

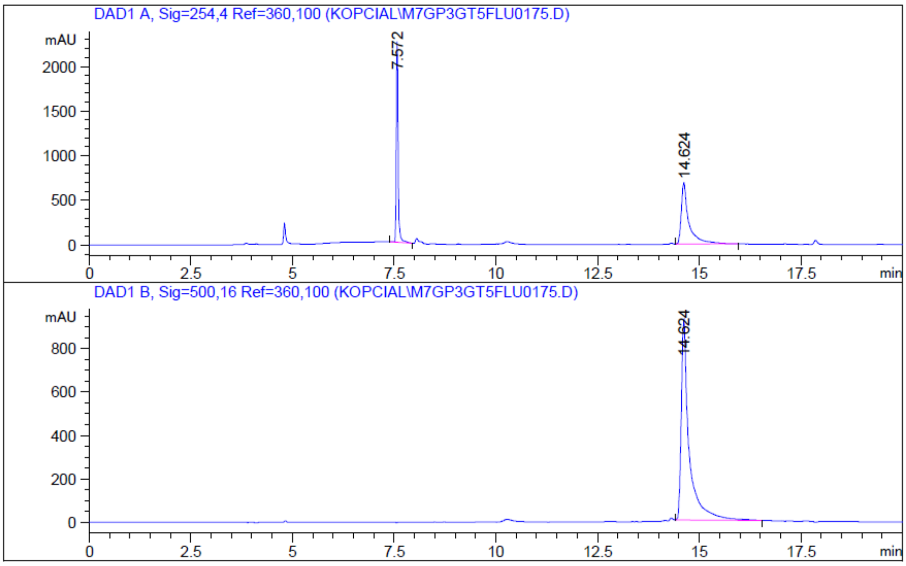

t = 1 h

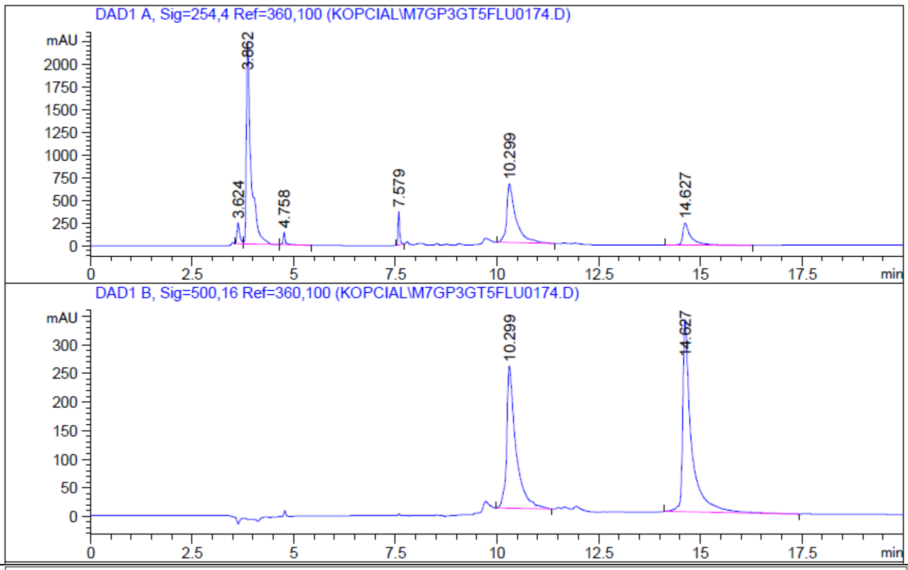

HPLC of purified product (method C)

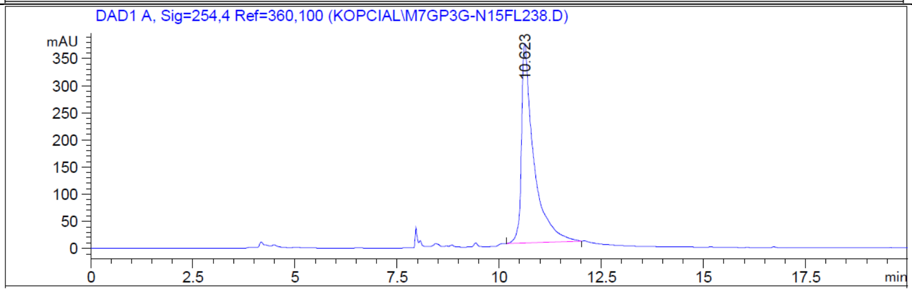

HR MS	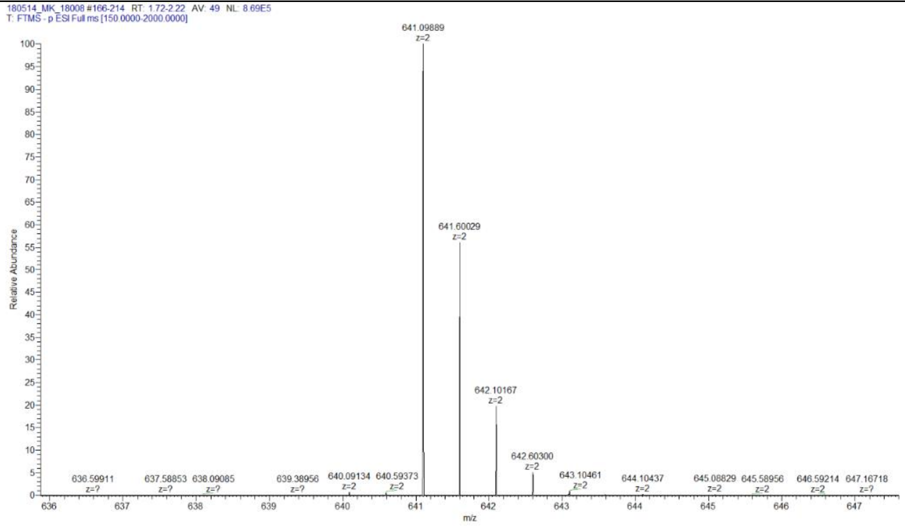

m^{7}Gp$_{3}$G-N1-Cy3 **(7)**	
Structure	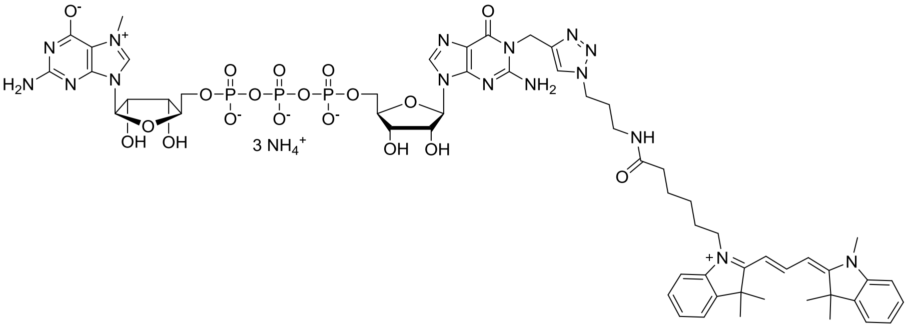

Reaction HPLC (Method C)

t = 0

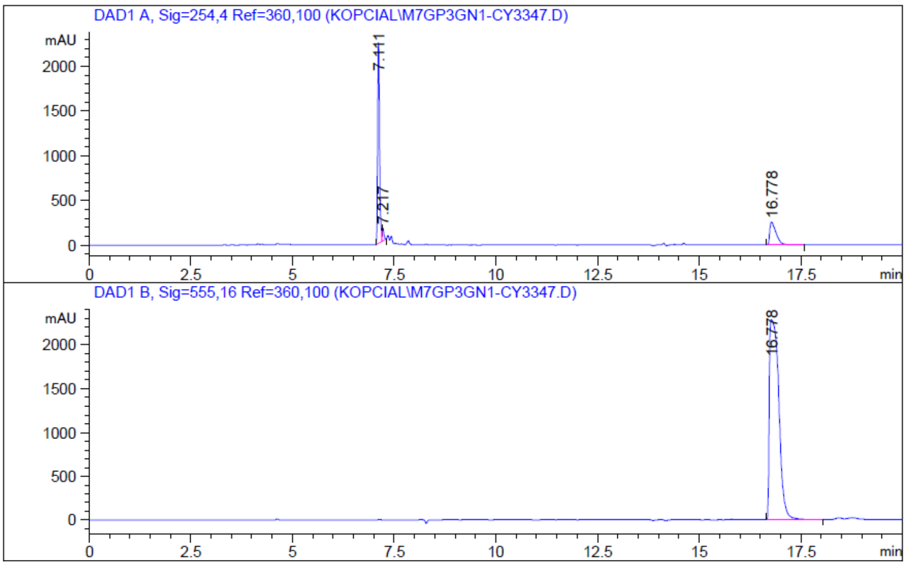

t = 1 h

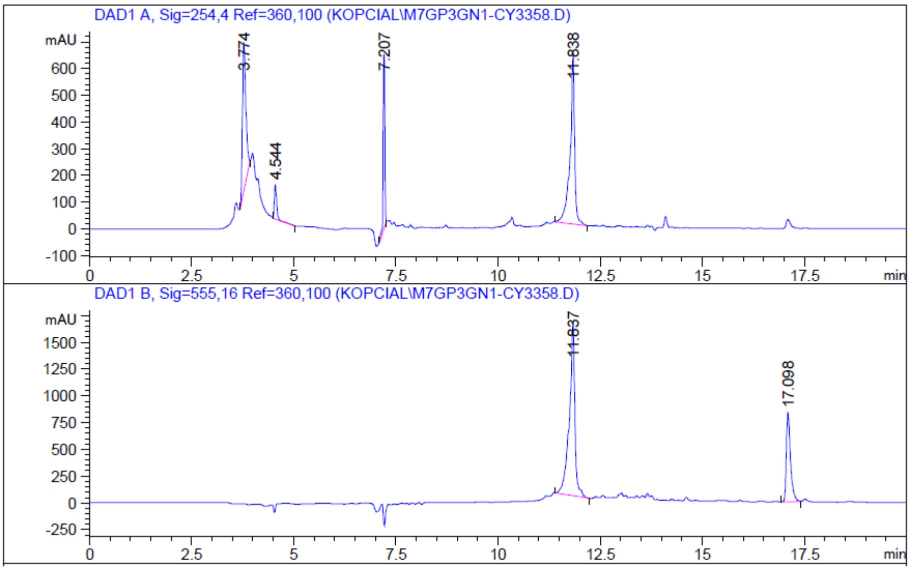

HPLC of purified product (method C)

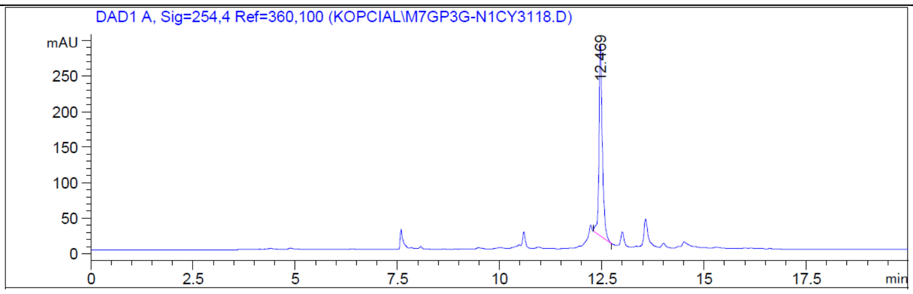

HR MS	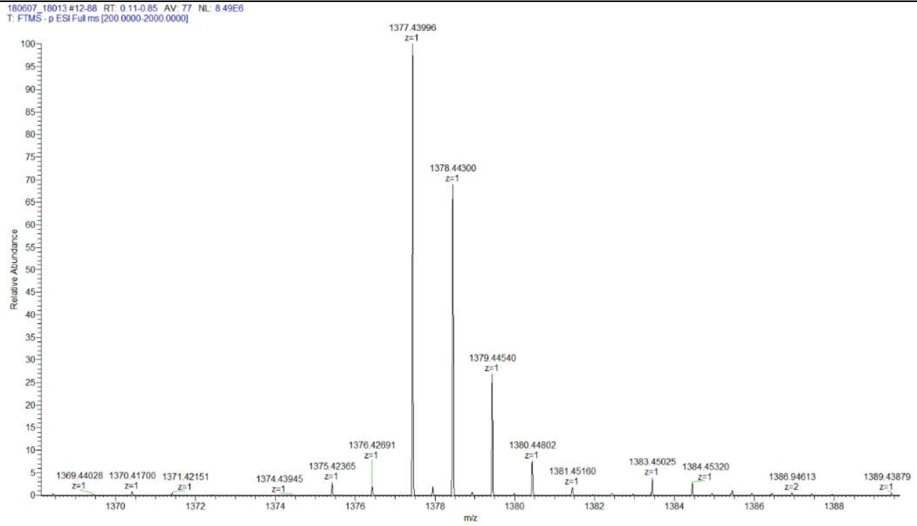

m^{7}Gp$_{3}$G-N1-Cy5 **(8)**	
Structure	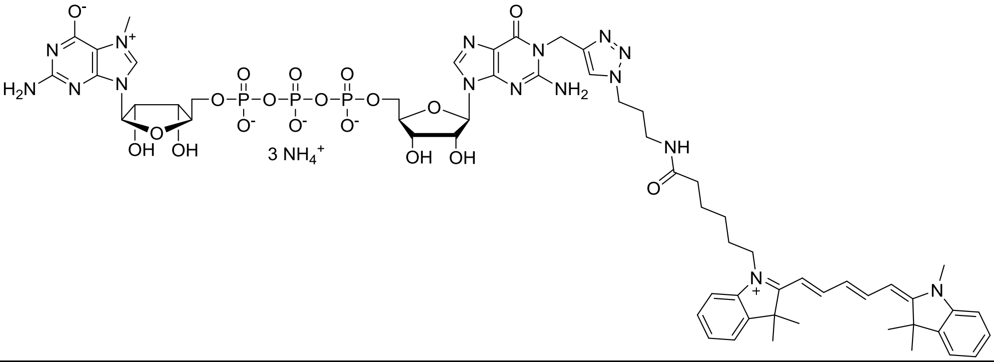

<table>
<tr>
<td>Reaction HPLC (Method C)</td>
<td>
t = 0

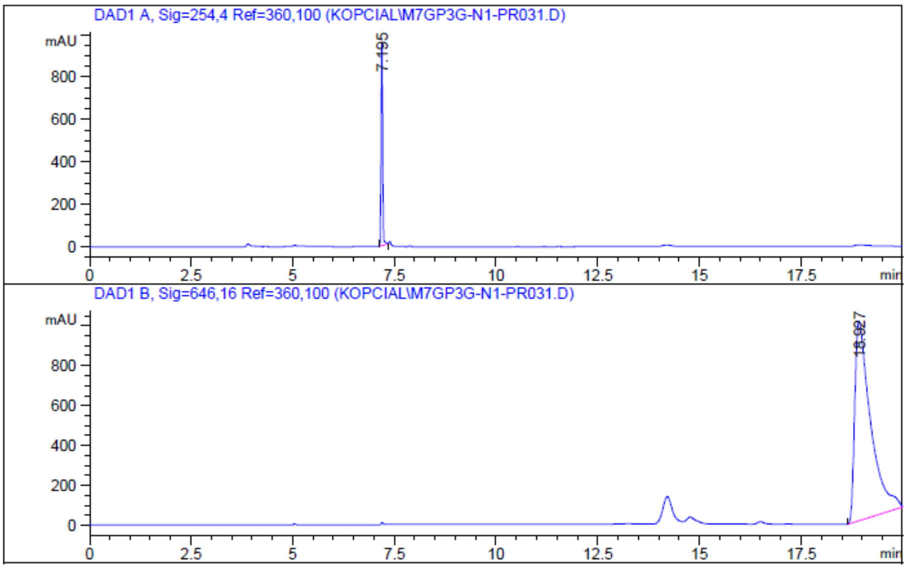

t = 50 min

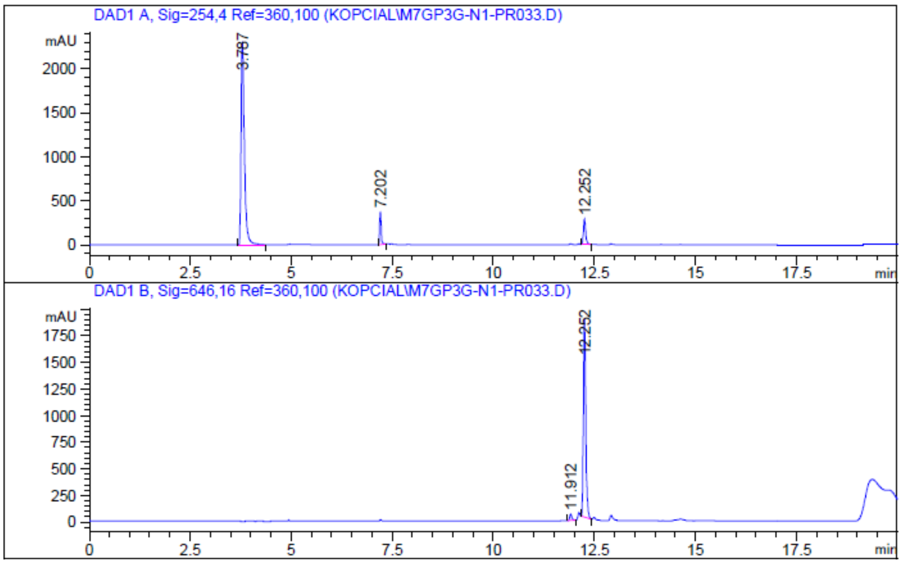

</td>
</tr>
<tr>
<td>HPLC of purified product (method C)</td>
<td>
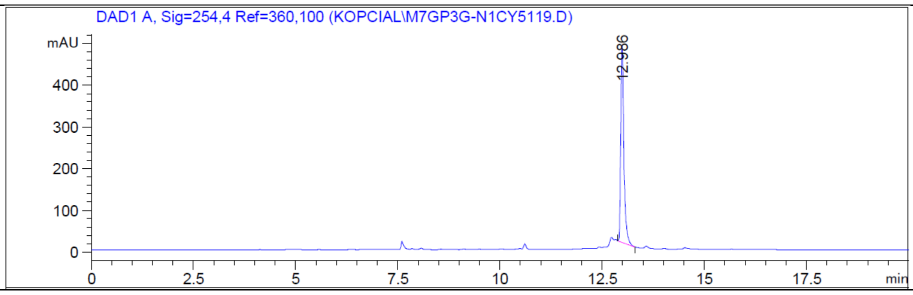

</td>
</tr>
</table>

HR MS	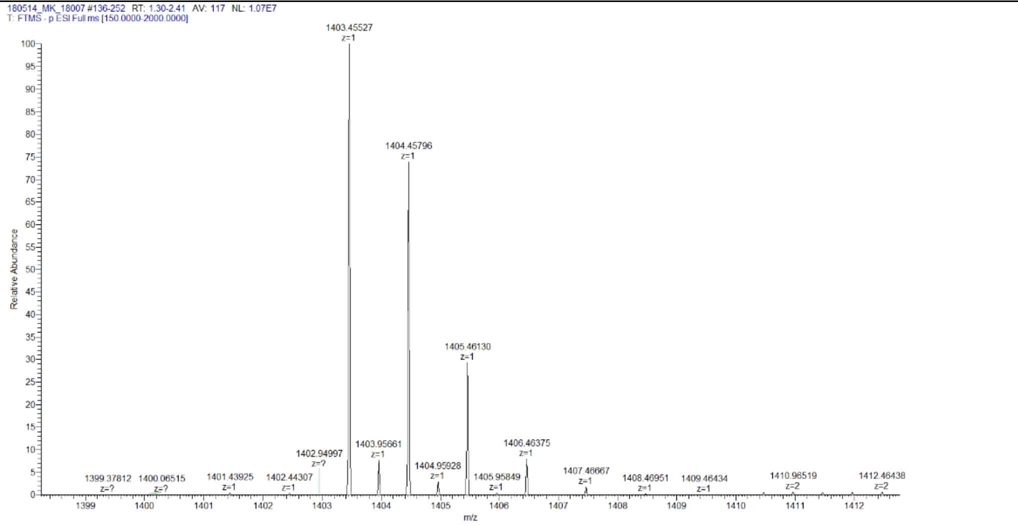

m⁷Gp₃G-N1-Py **(9)**	
Structure	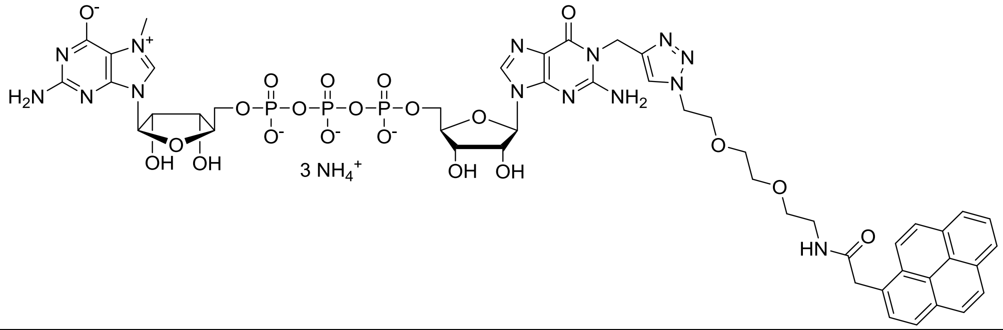

Reaction HPLC (Method C)	t = 0<br>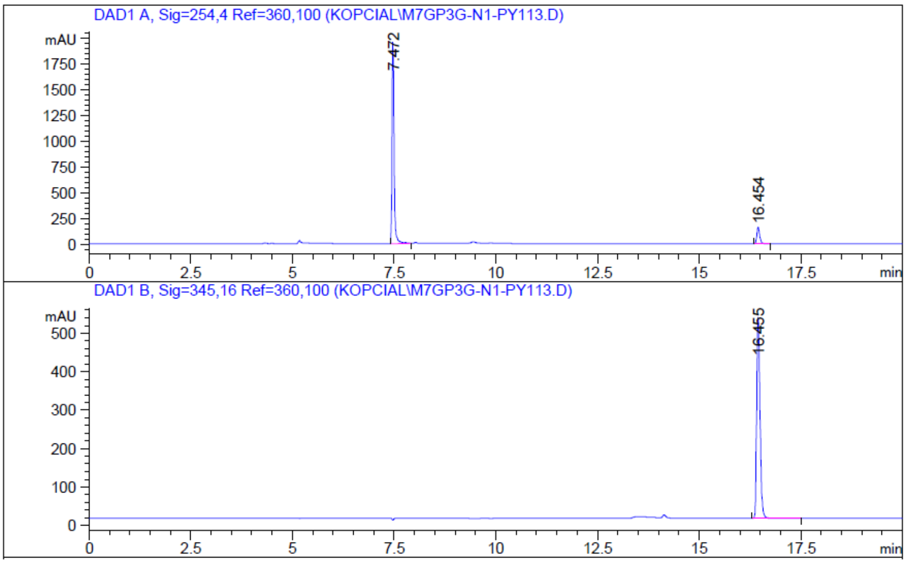<br>t = 48 h<br>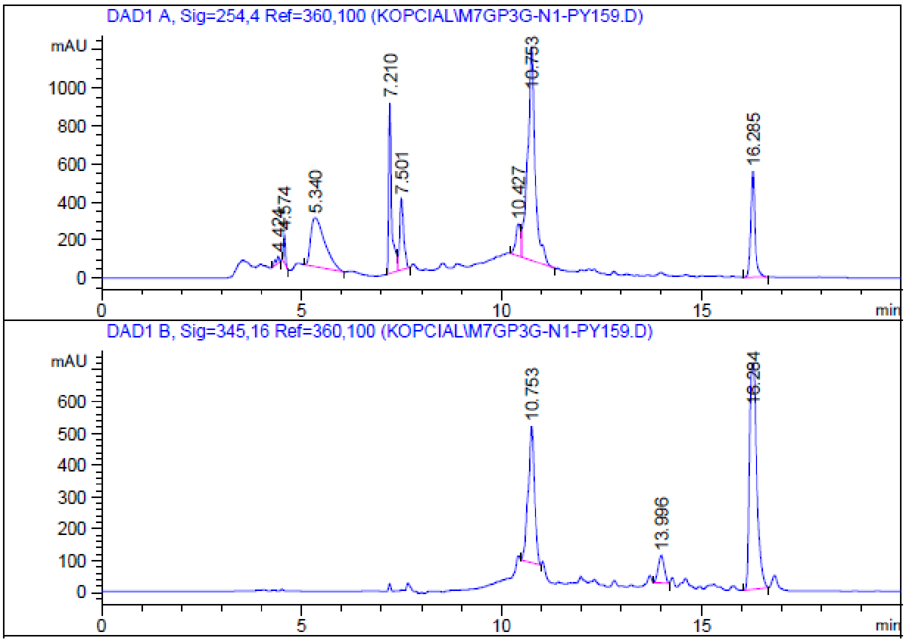
HPLC of purified product (method C)	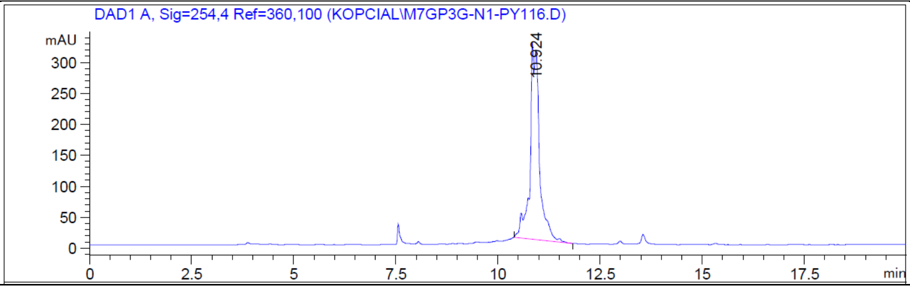

HR MS

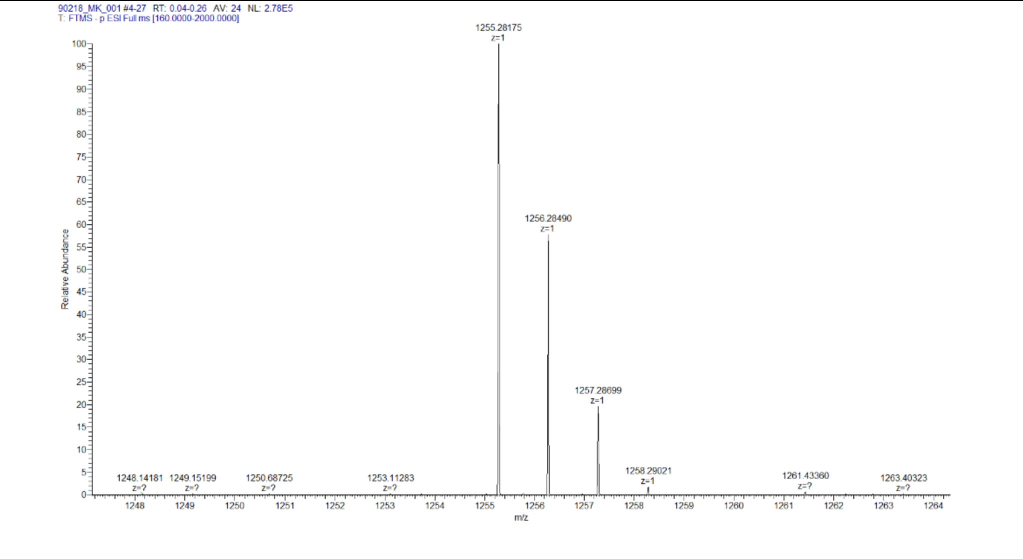

m^7Gp_3G-N1-biotin **(10)**

Structure

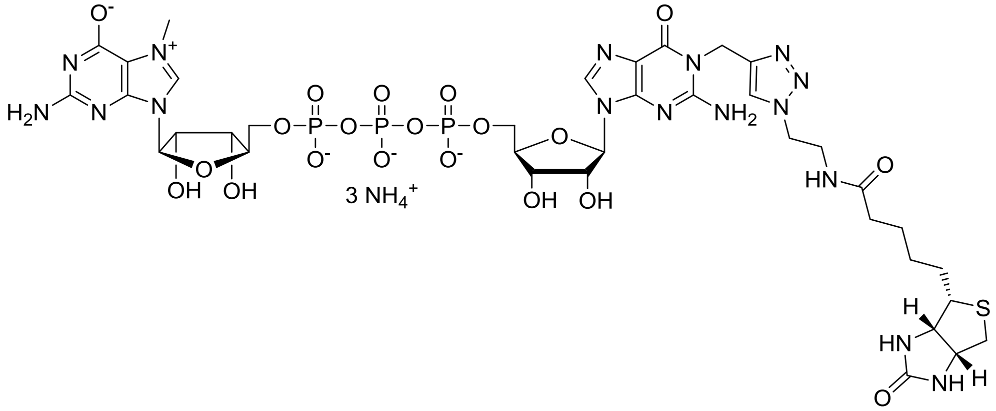

Reaction HPLC (Method C)

t = 0

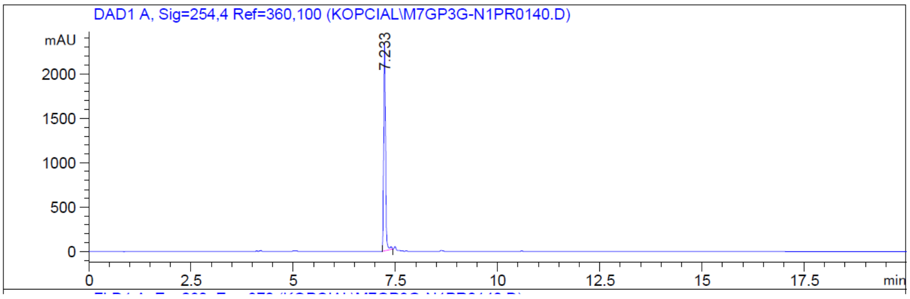

t = 1 h

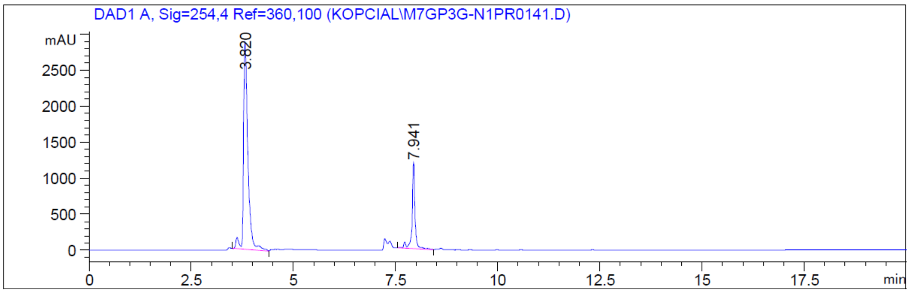

HPLC of purified product (method C)	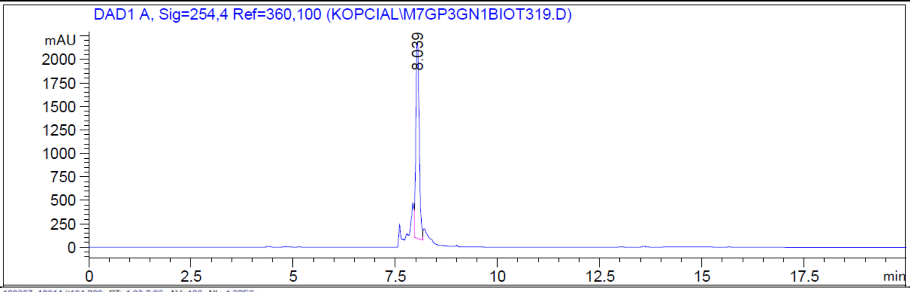
HR MS	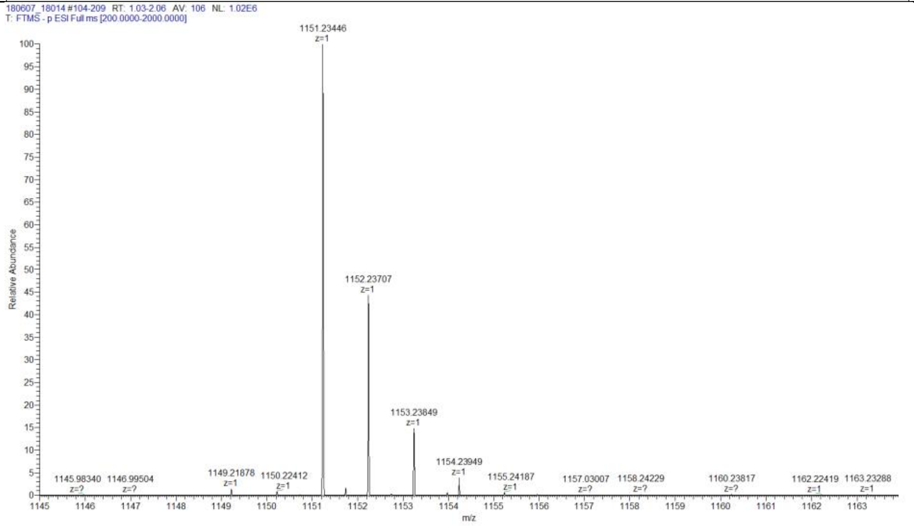

m^{7}GpCH$_2$ppG-N1-5FAM **(11)**	
Structure	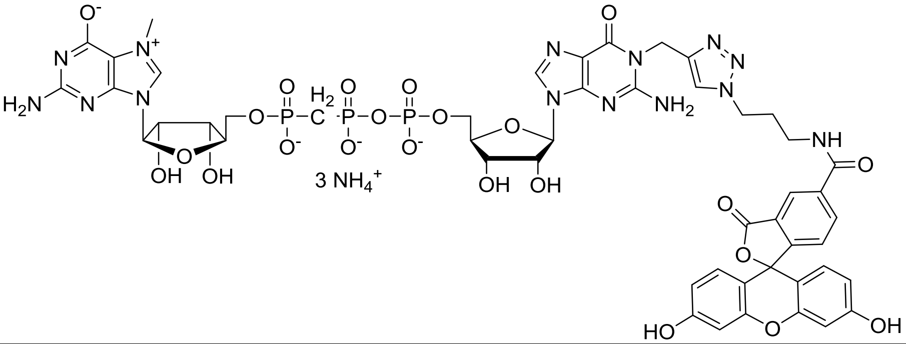

Reaction HPLC (Method D)	
	t = 0

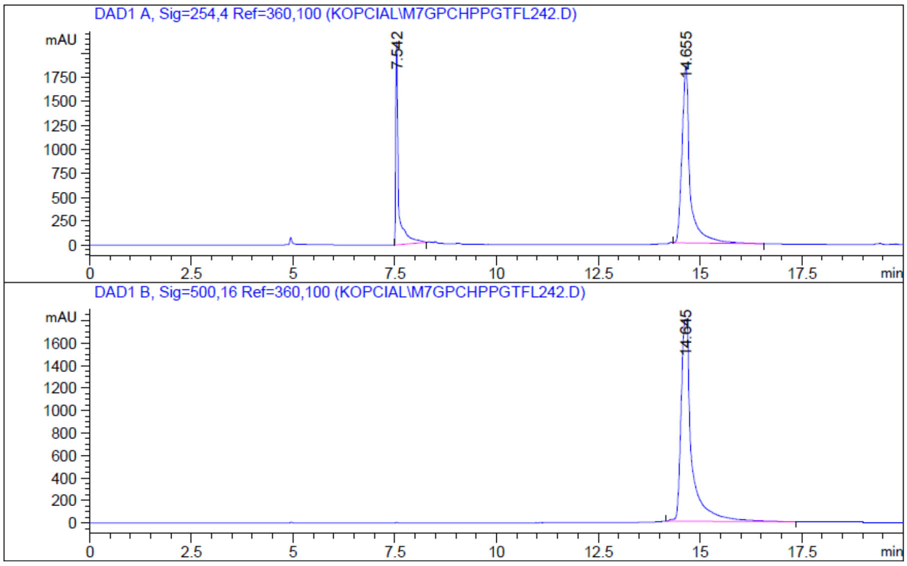

t = 1 h

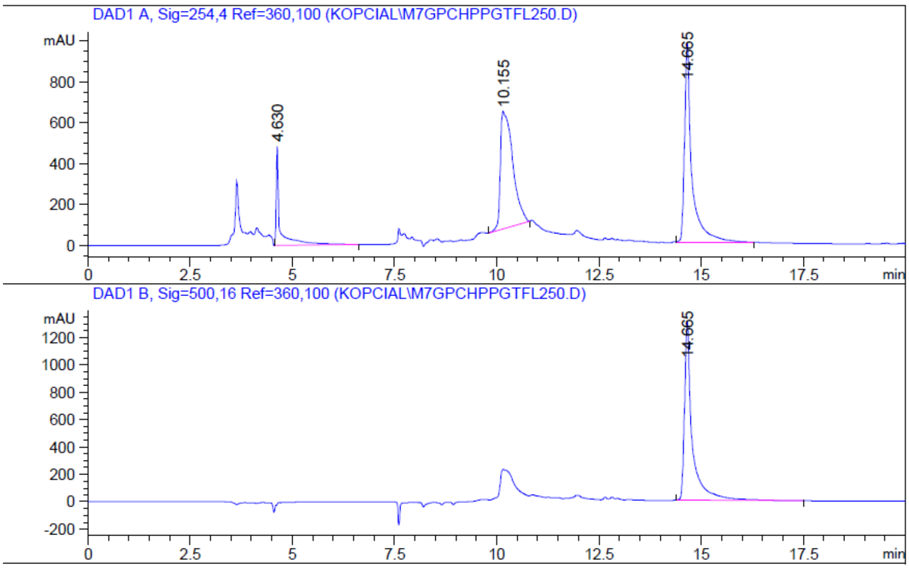

HPLC of purified product (method C)

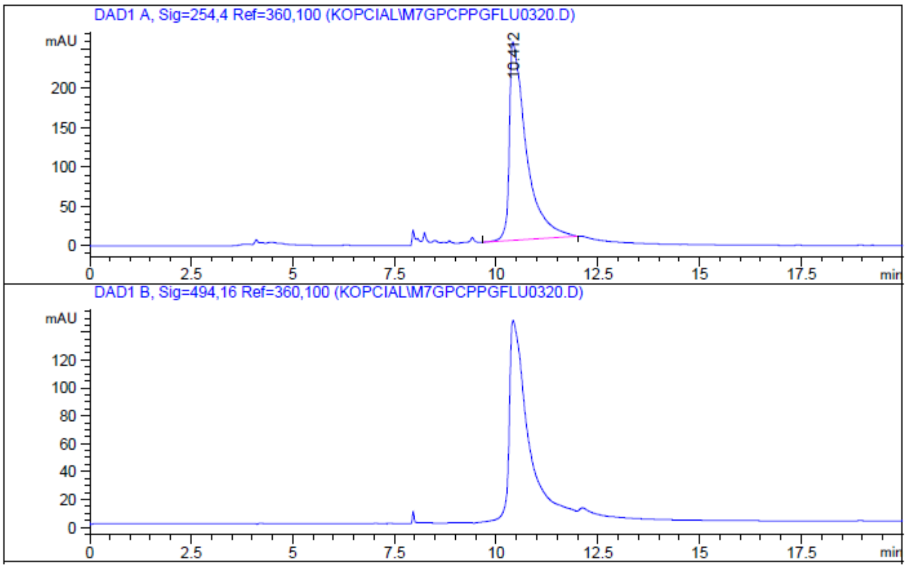

HR MS

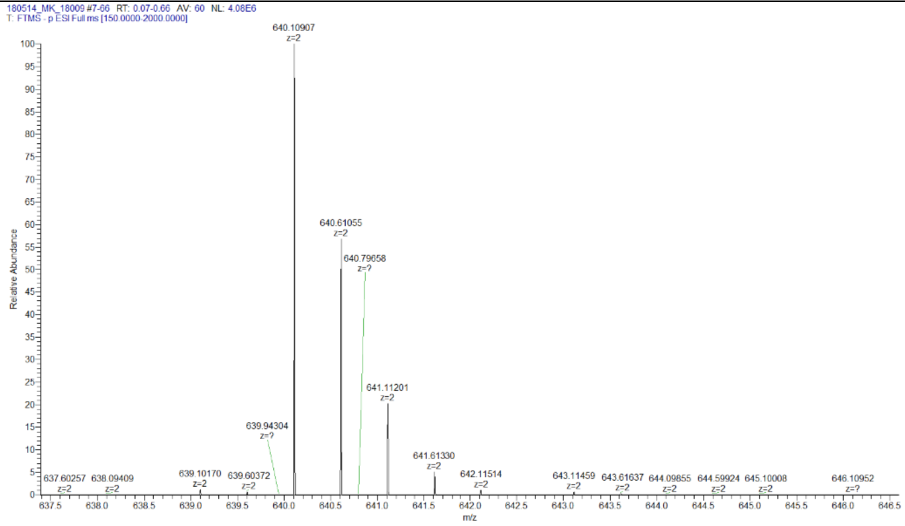

m^{7}GpNHppG-N1-5FAM **(12)**

Structure

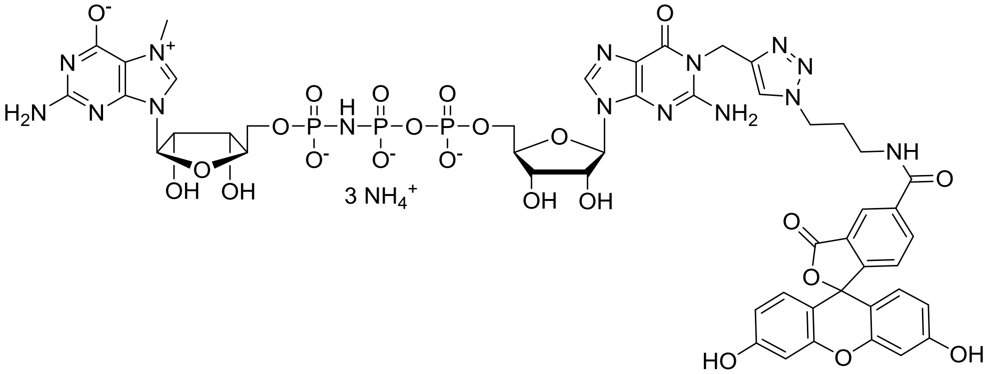

Reaction HPLC (Method D)	t = 0 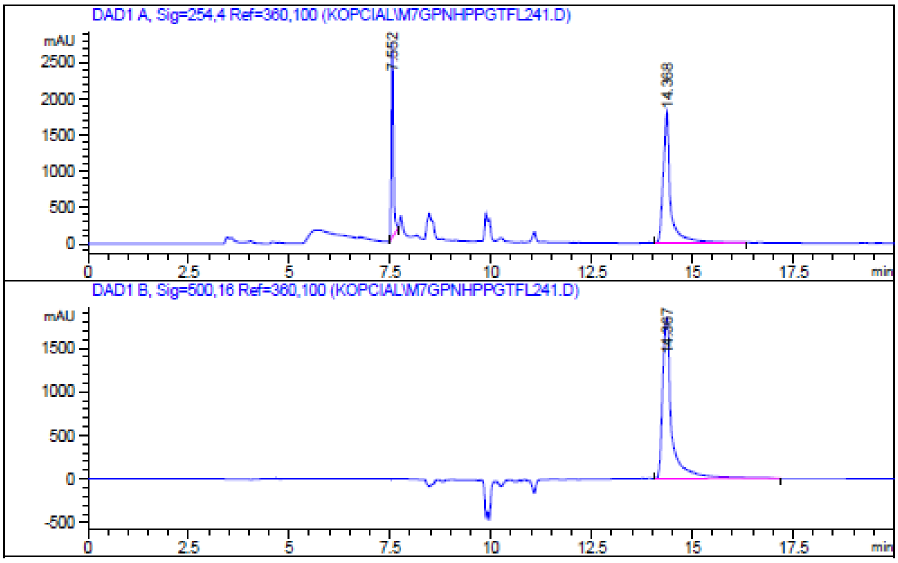 t = 1 h 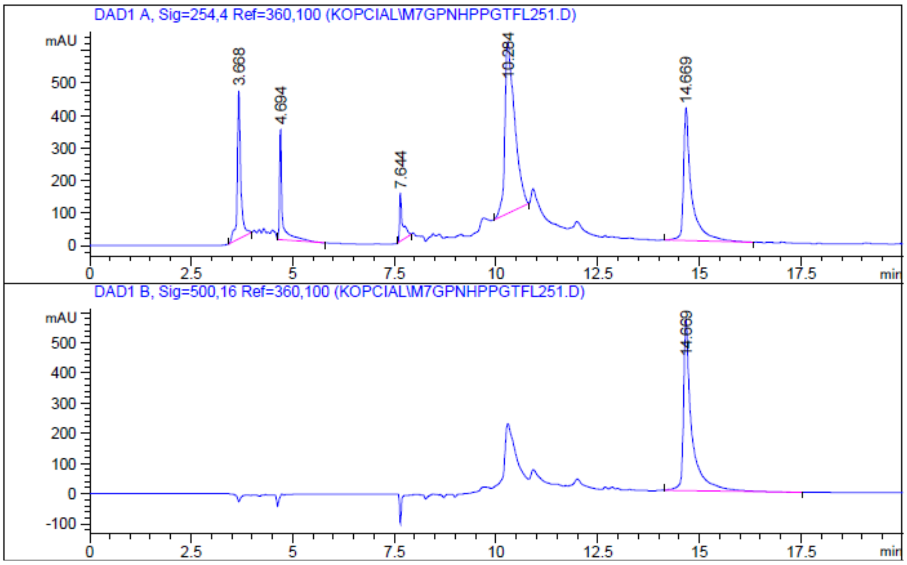
HPLC of purified product (method C)	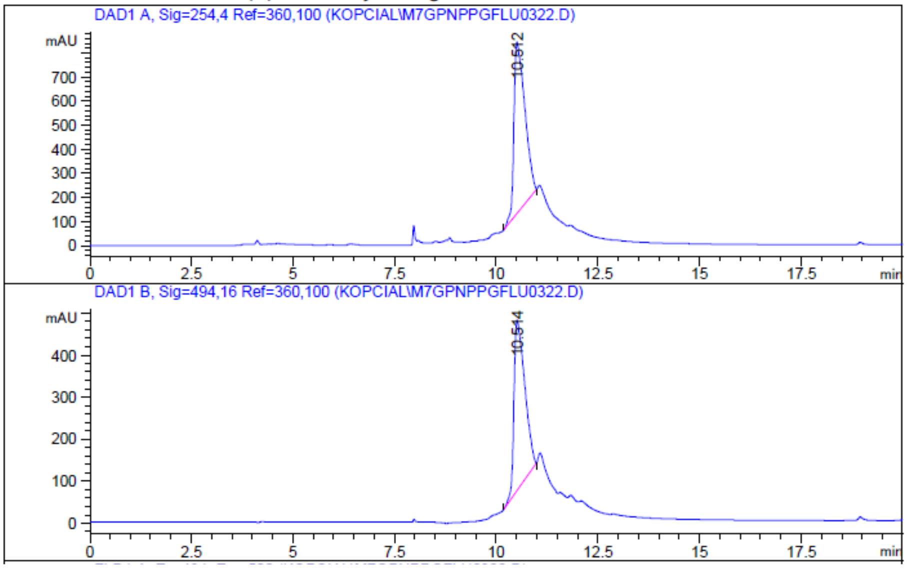

HR MS	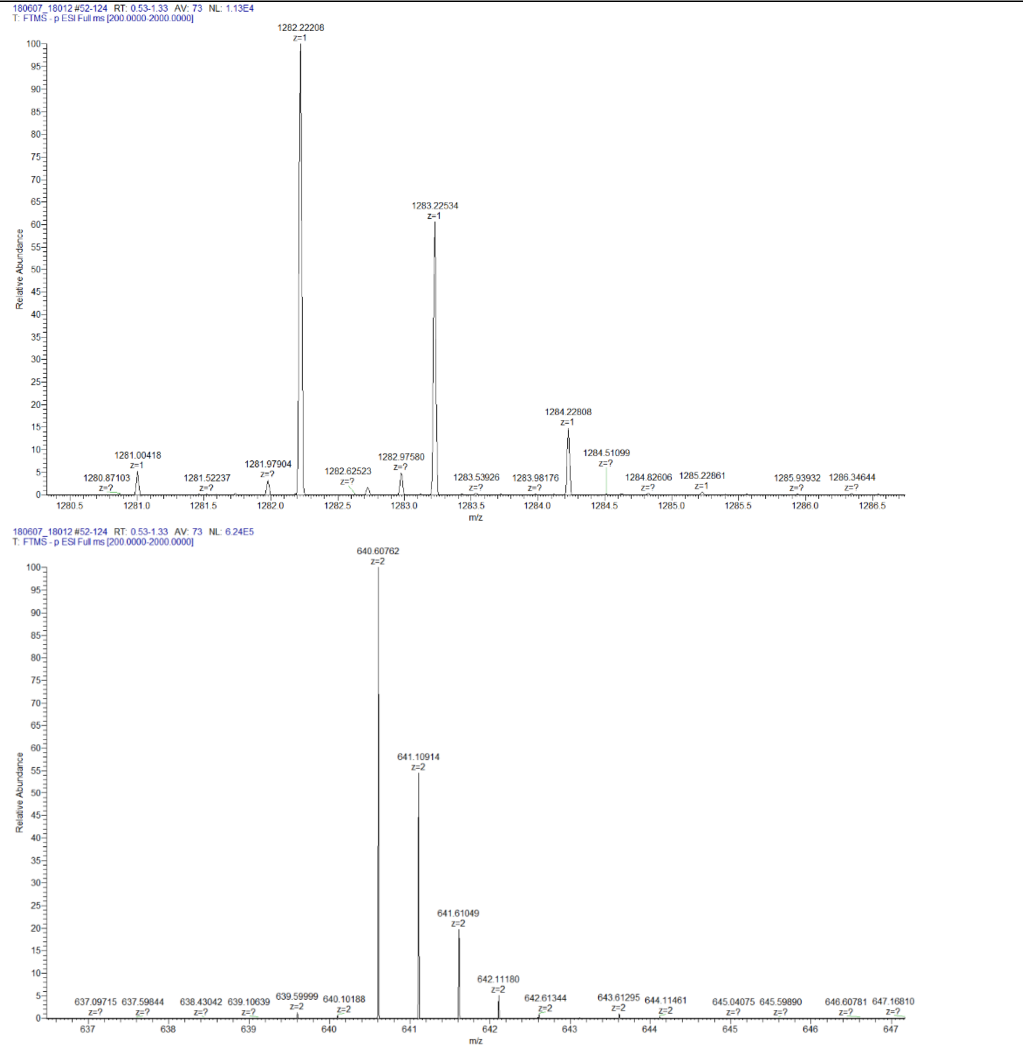

m^{7}GppCH$_2$pG-N1-5FAM **(13)**	
Structure	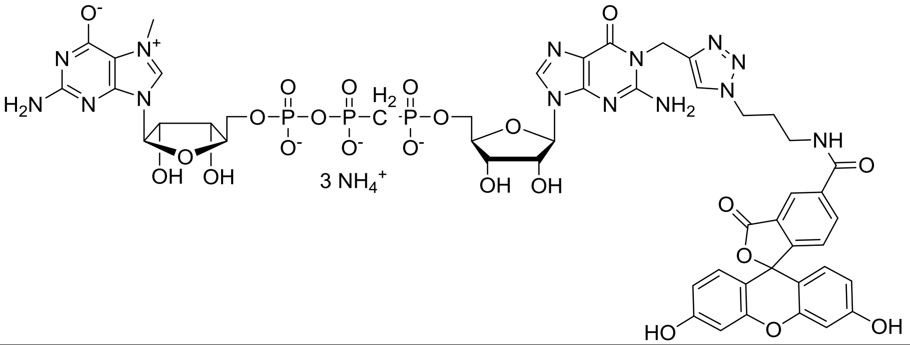

Reaction HPLC (Method C)

t = 0

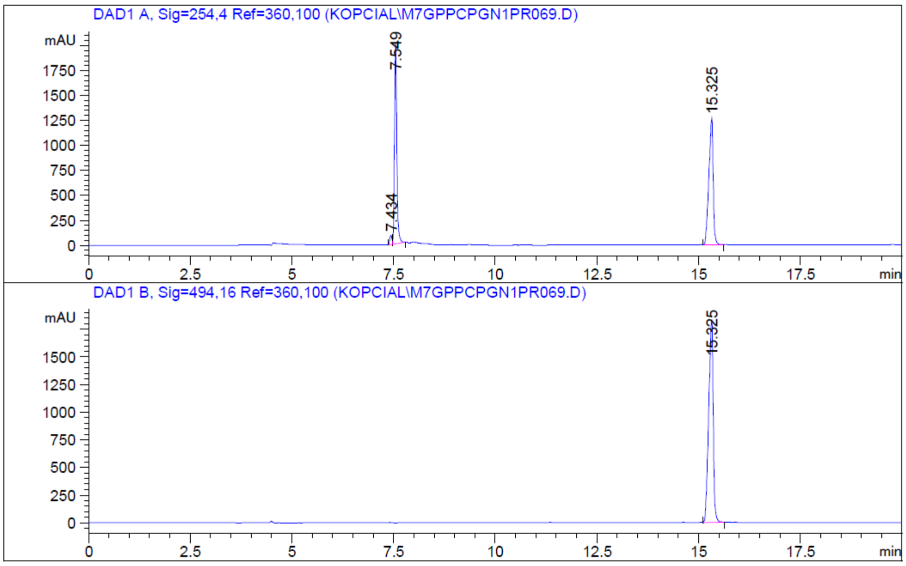

t = 30 min

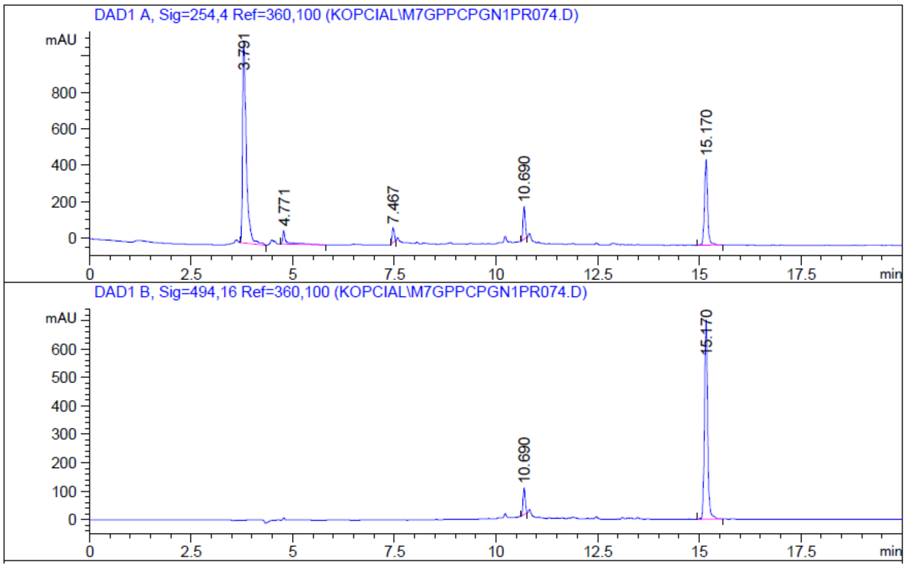

HPLC of purified product (method C)

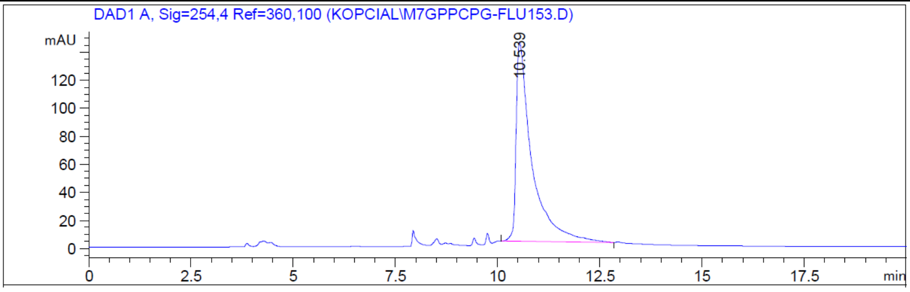

HR MS	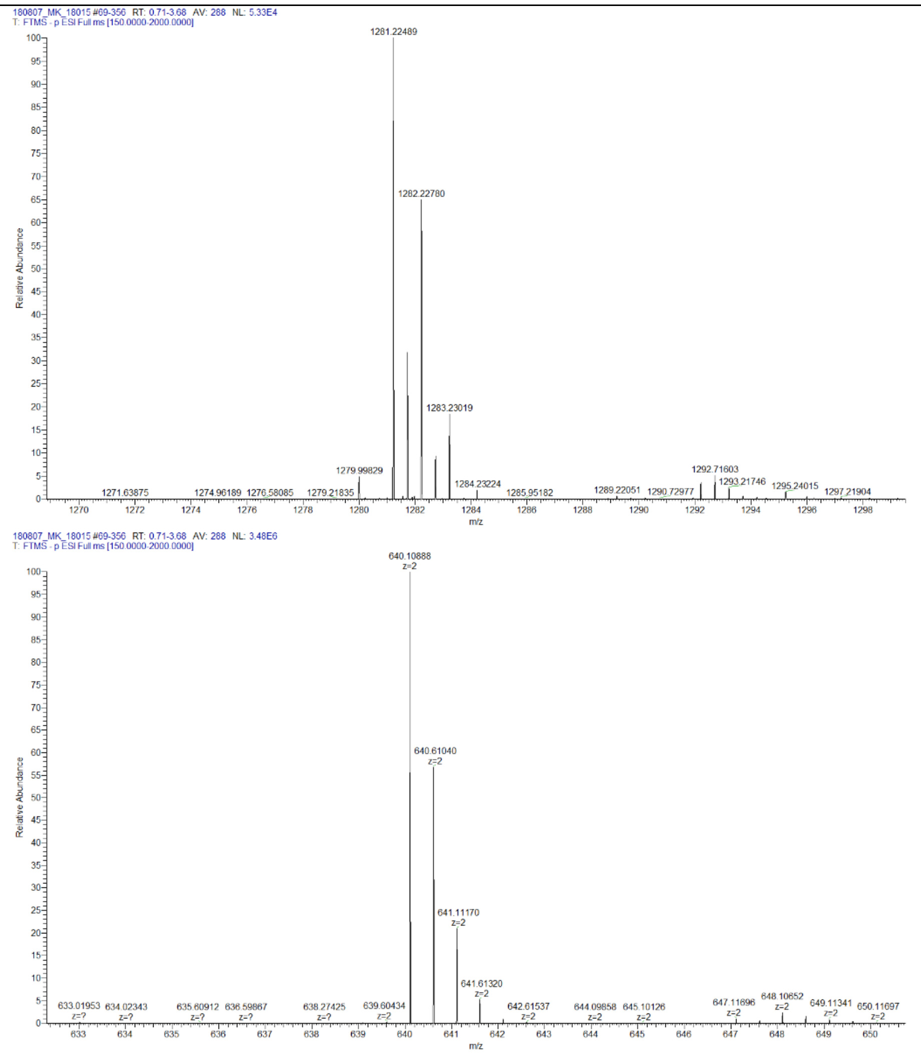

m[7]GppNHpG-N1-5FAM **(14)**	
Structure	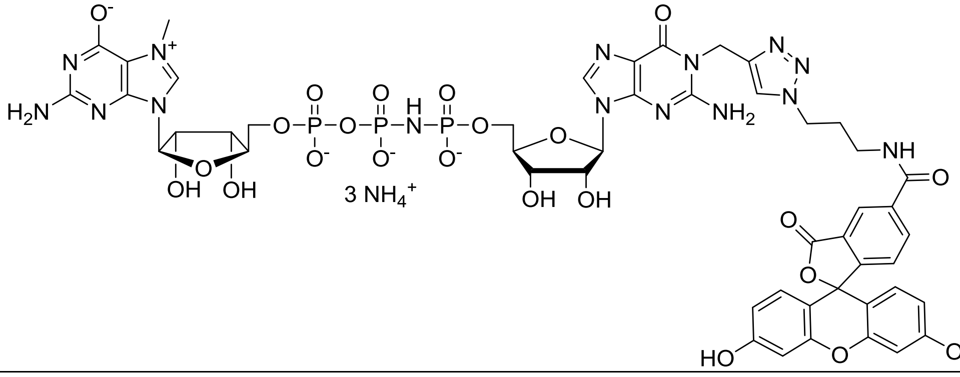

Reaction HPLC (Method D)

t = 0

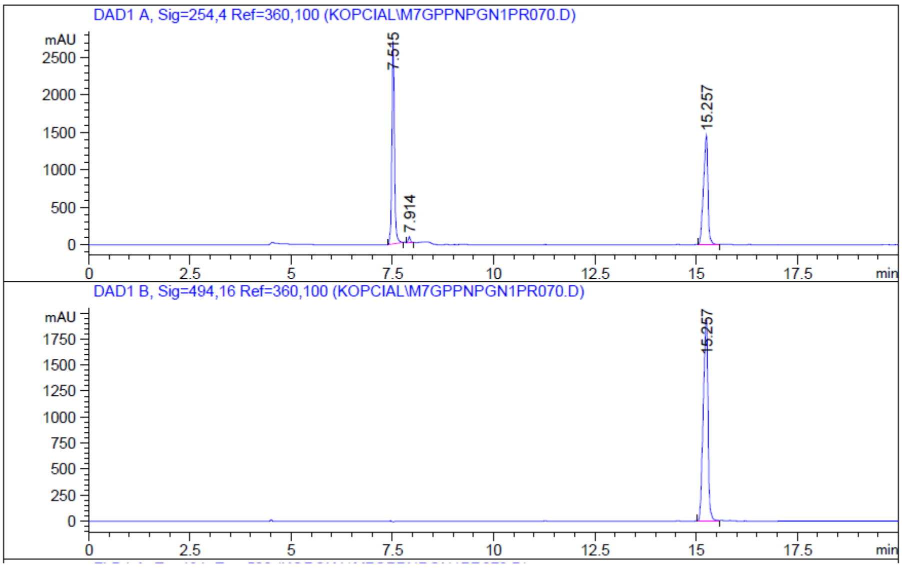

t = 1 h

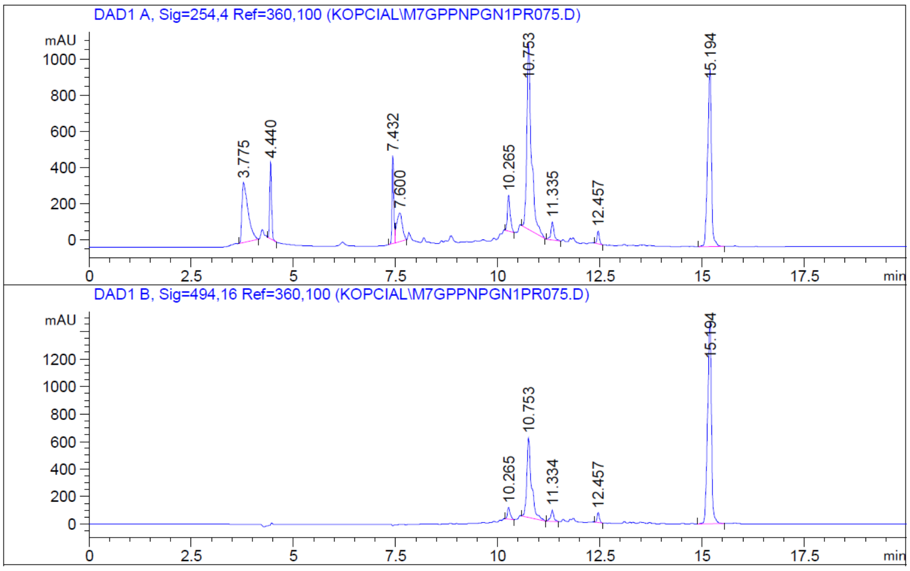

HPLC of purified product (method D)

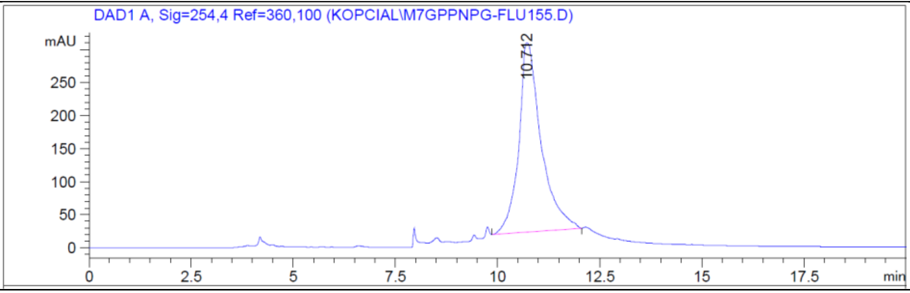

HR MS

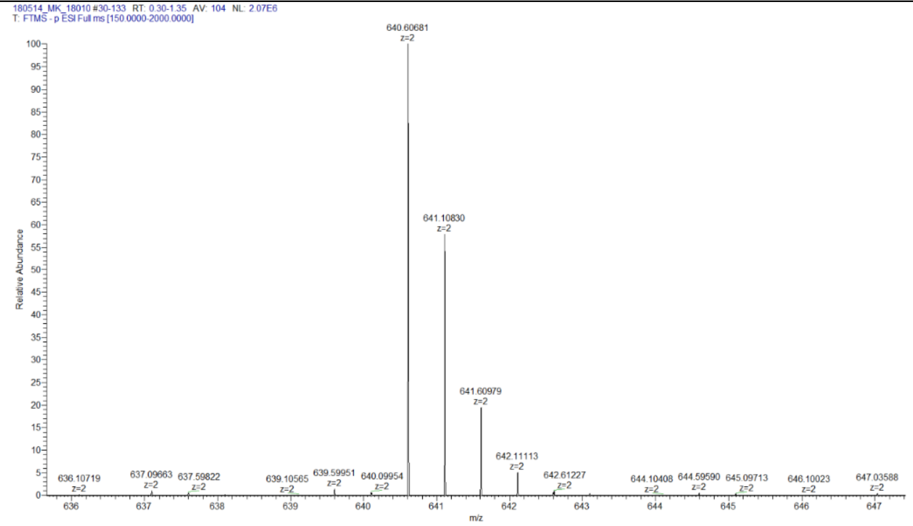

propargyl-N1-Guo **(16)**

Structure

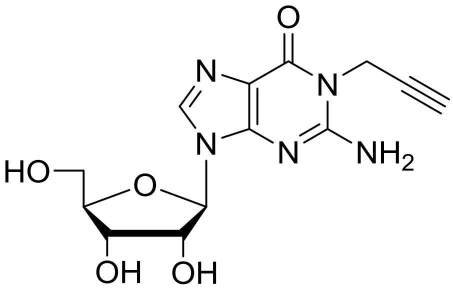

HRMS

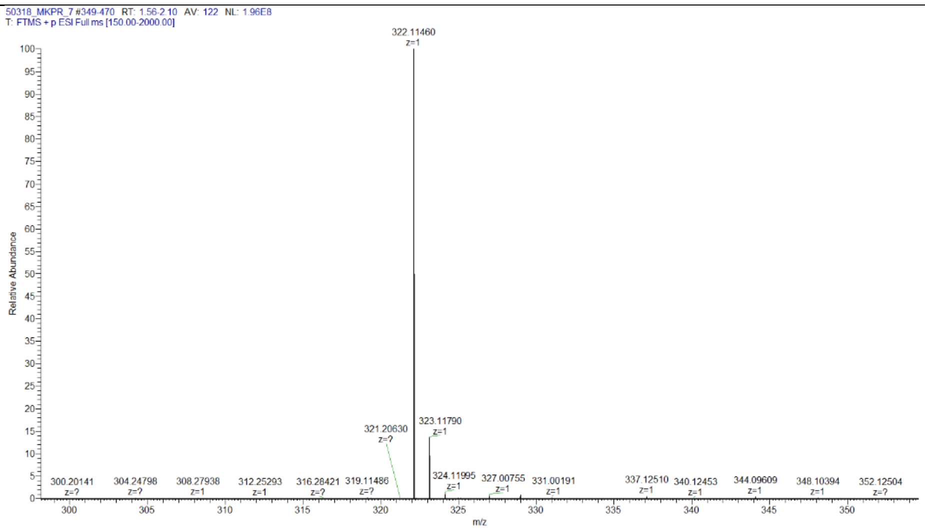

^{1}H NMR (400 MHz, DMSO-d_6)

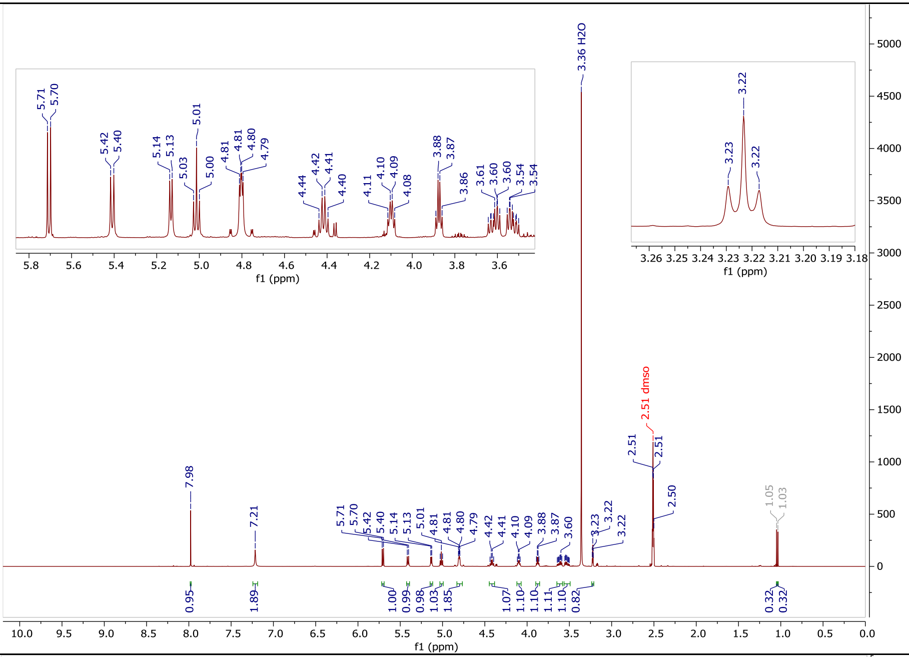

^{13}C NMR (162 MHz, D_2O)

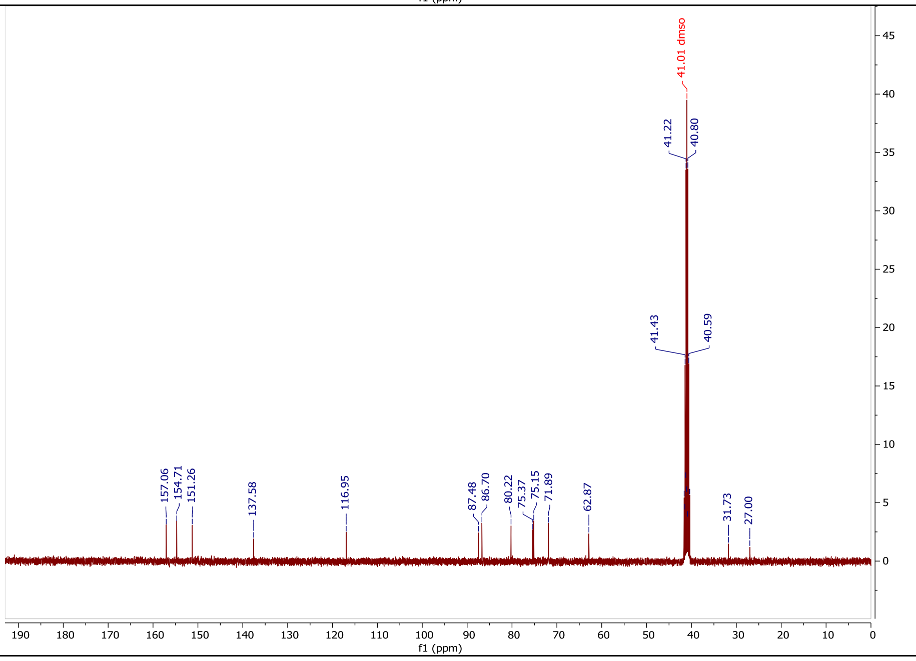

propargyl-N1-GMP **(17)**

Structure

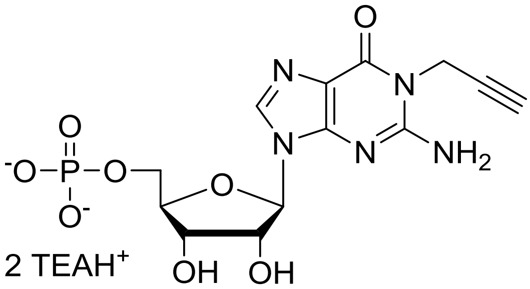

HPLC of purified product (method A)	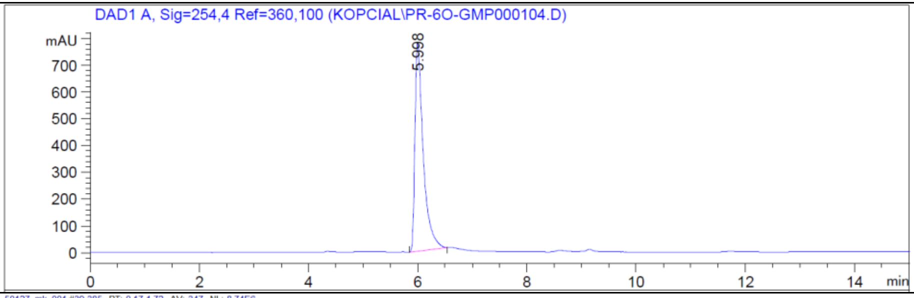
HRMS	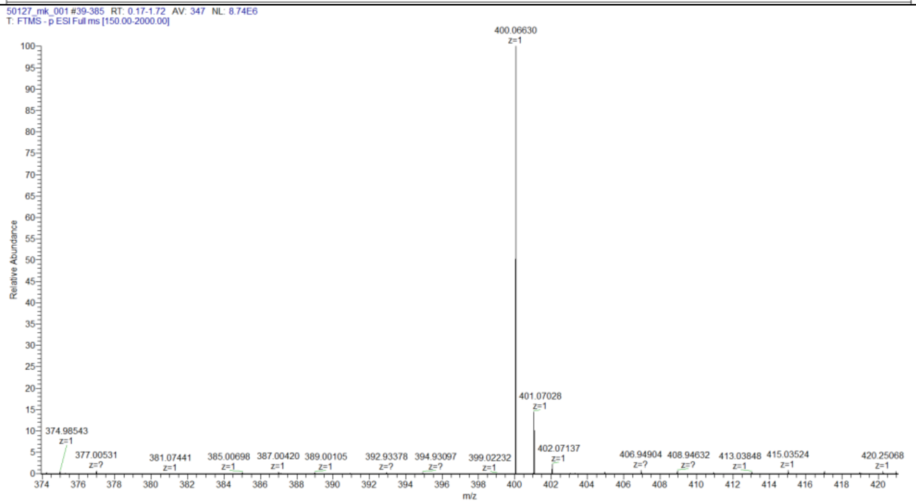
^1H NMR (400 MHz, D_2O)	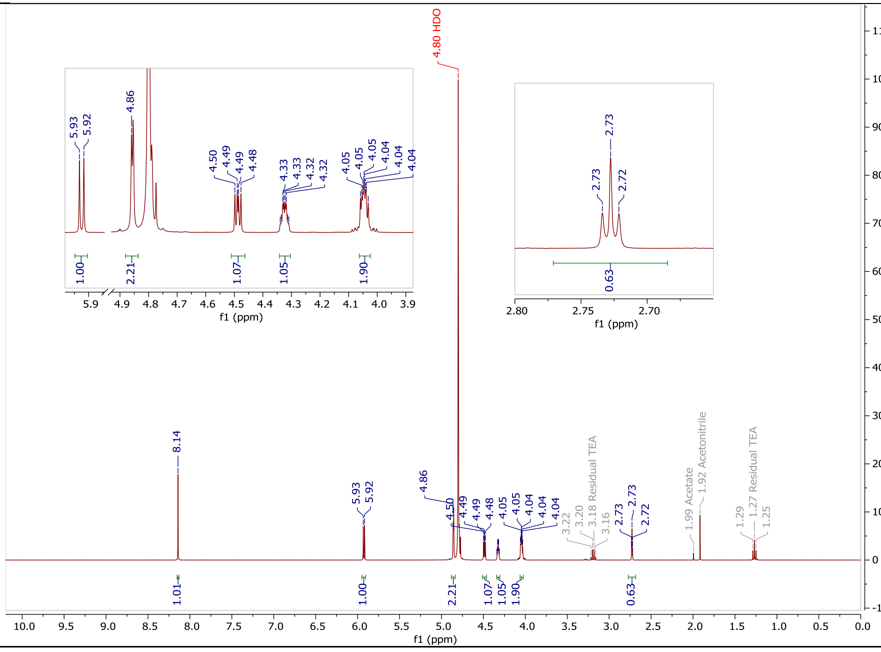

^{31}P NMR (162 MHz, D_2O)	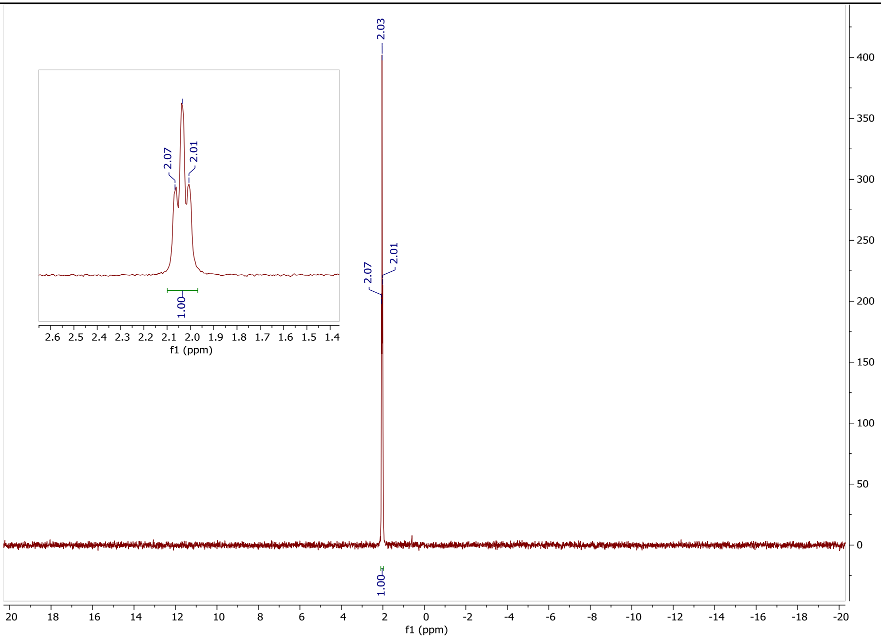

propargyl-N1-GMP-Im **(18)**

Structure	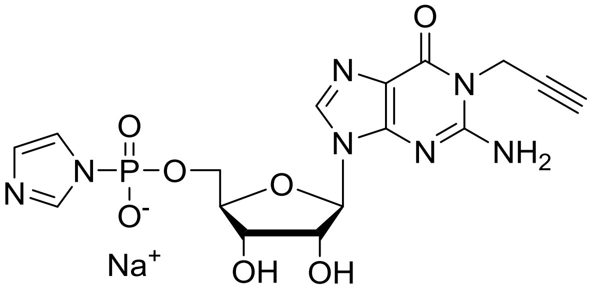
HPLC of purified product (method A)	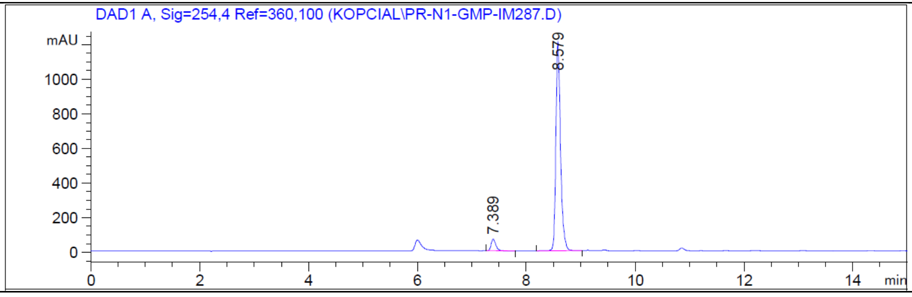

HRMS	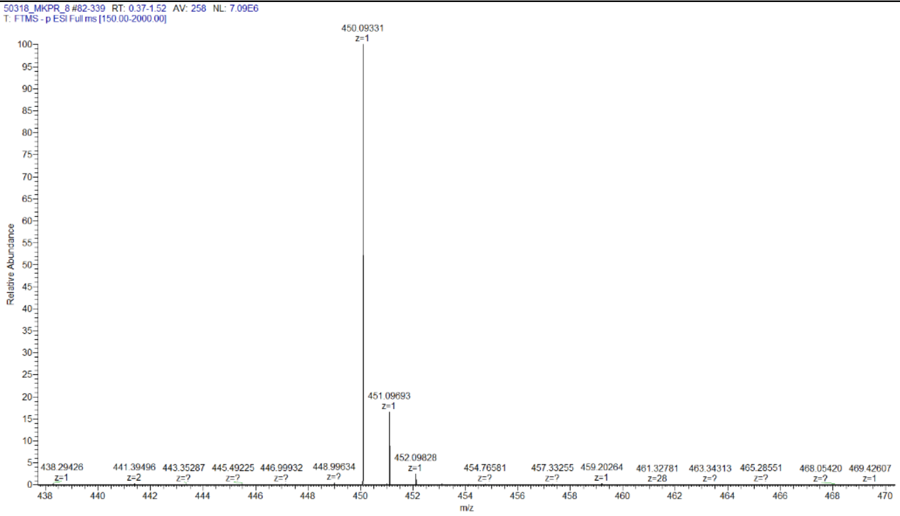
^{1}H NMR (400 MHz, D_2O)	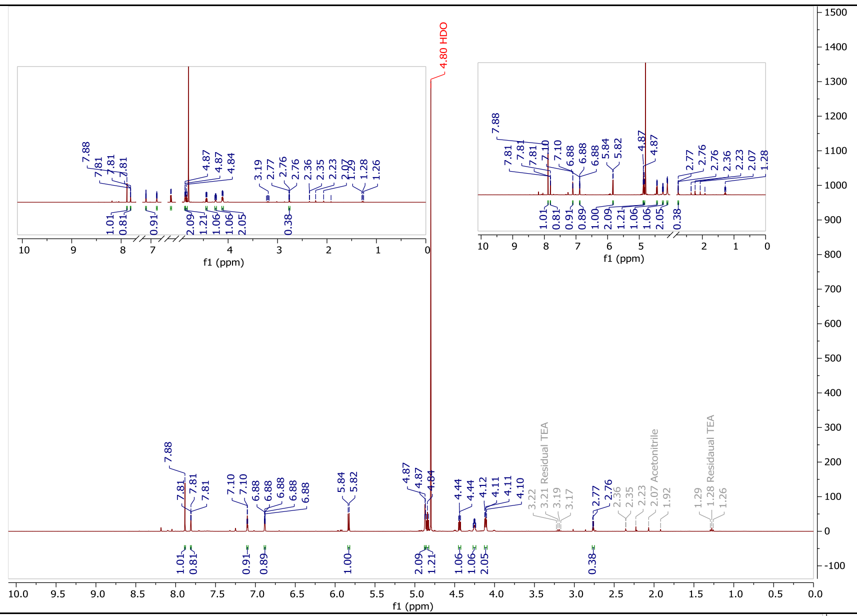
^{31}P NMR (162 MHz, D_2O)	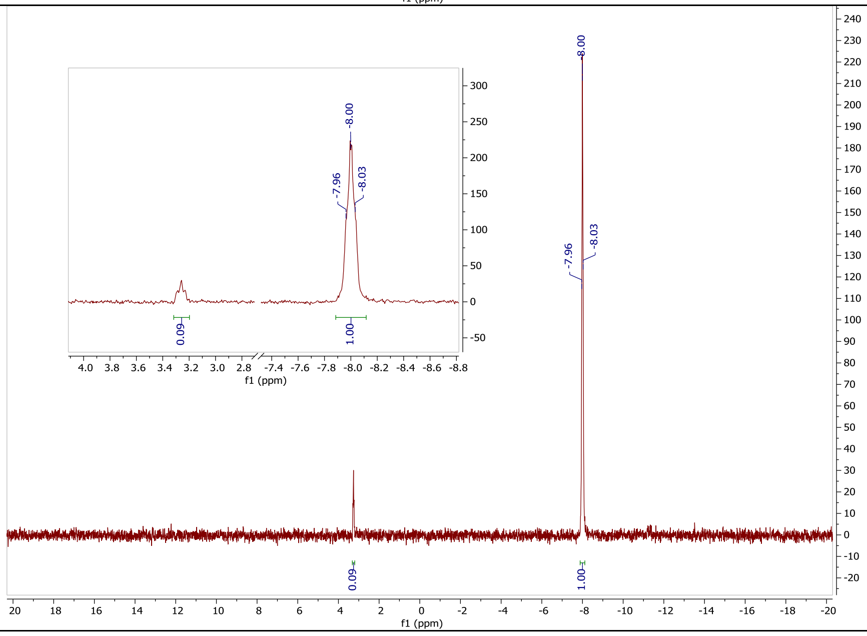

propargyl-N1-GpCH$_2$p **(22)**

Structure

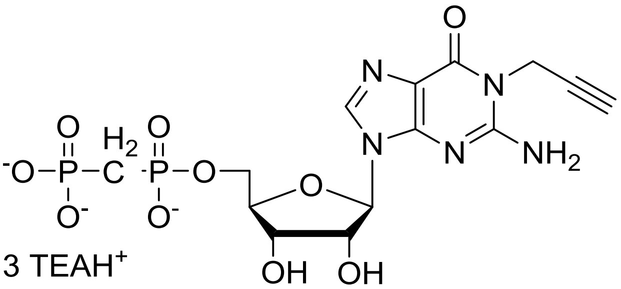

HPLC of purified product (method A)

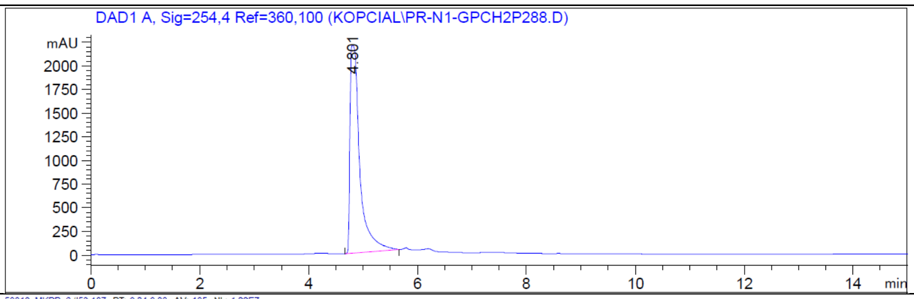

HRMS

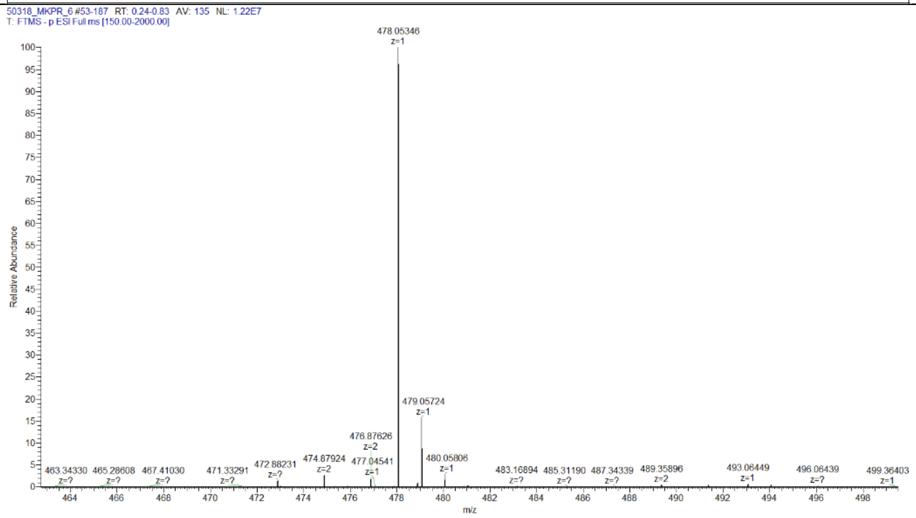

1H NMR (400 MHz, D$_2$O)

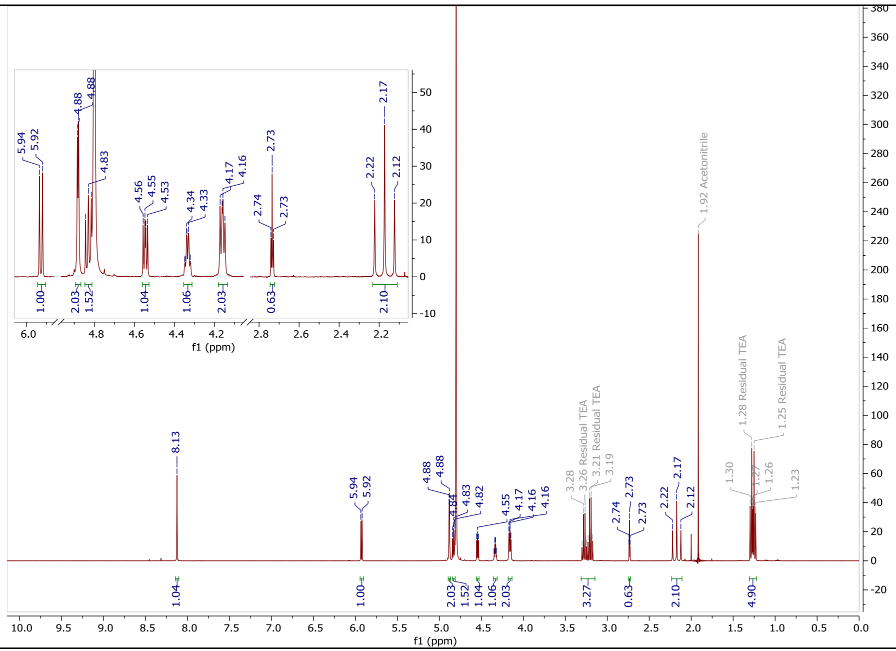

^{31}P NMR (162 MHz, D_2O)	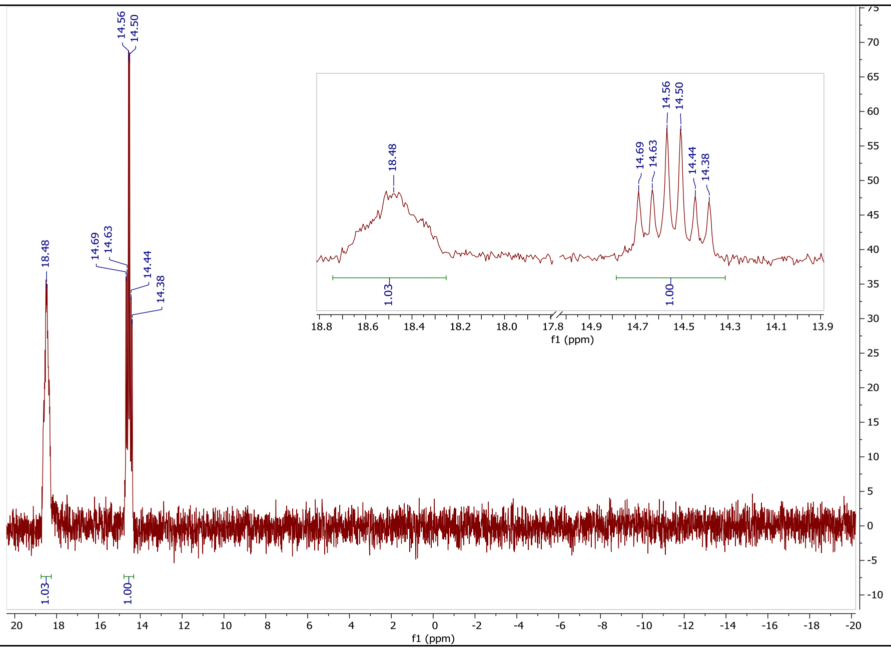

propargyl-N1-GpNHp **(23)**	
Structure	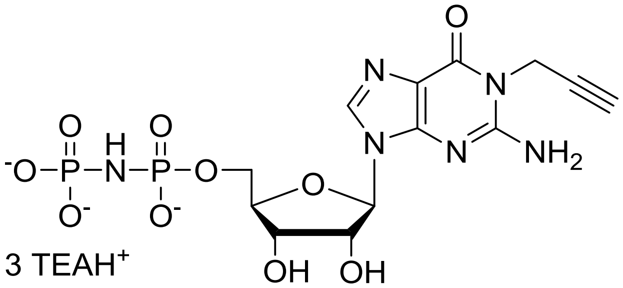
HPLC of purified product (method A)	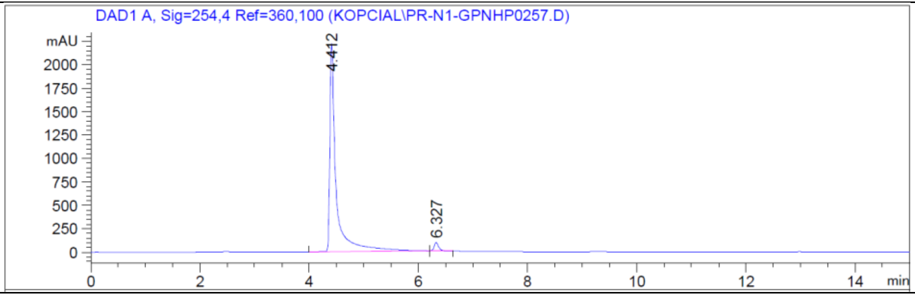

HRMS	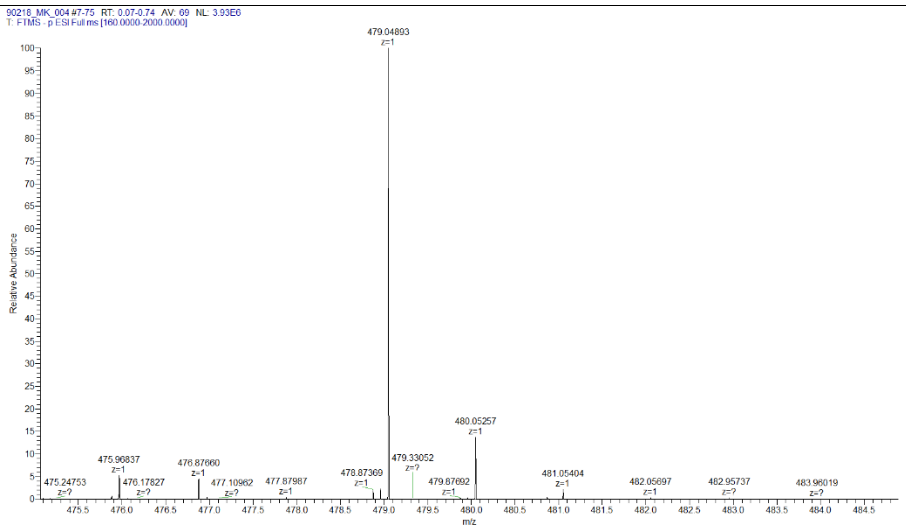
^1H NMR (400 MHz, D_2O)	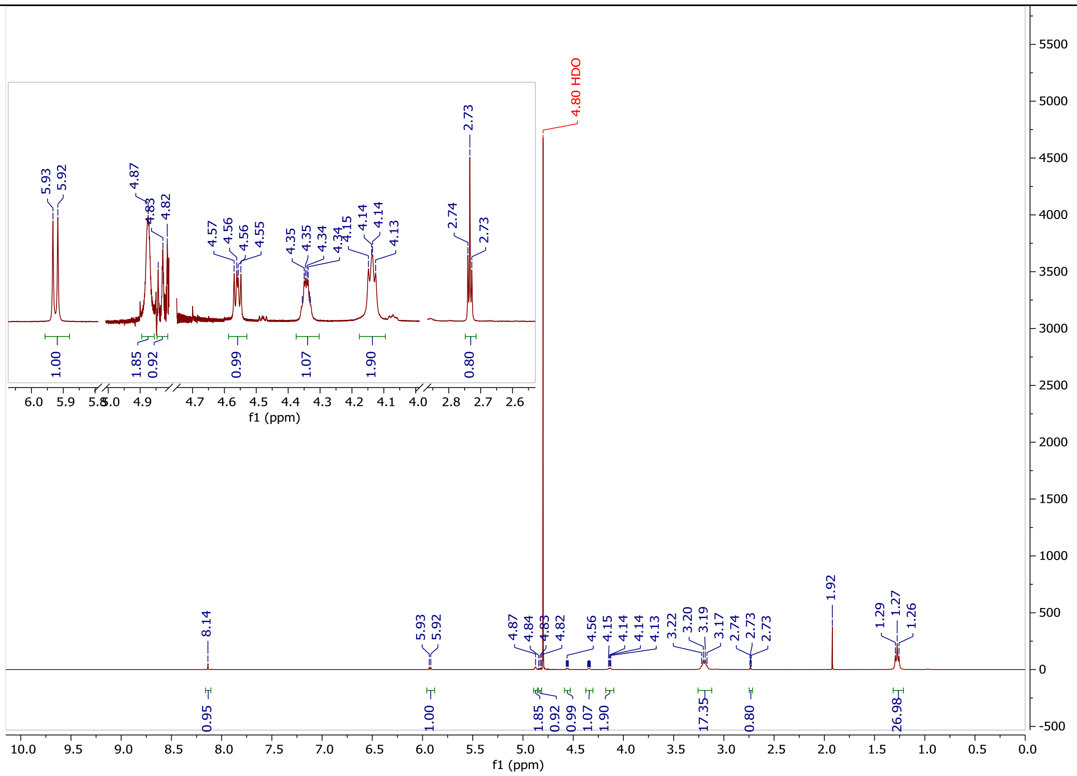

^{31}P NMR (162 MHz, D_2O)

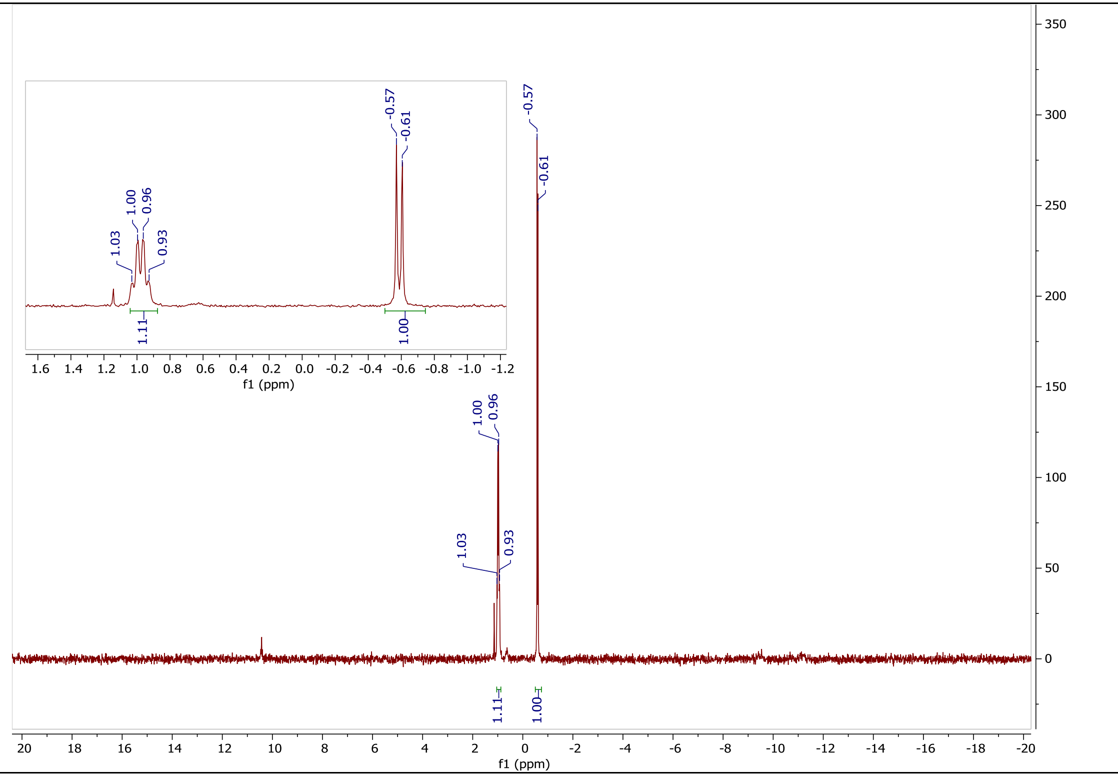

Supplement: Supplementary file 1 [file molecules-24-01899-s001.pdf]
